# Mapping Lexical Dialect Variation in British English Using Twitter

**DOI:** 10.3389/frai.2019.00011

**Published:** 2019-07-12

**Authors:** Jack Grieve, Chris Montgomery, Andrea Nini, Akira Murakami, Diansheng Guo

**Affiliations:** ^1^Department of English Language and Linguistics, University of Birmingham, Birmingham, United Kingdom; ^2^School of English, University of Sheffield, Sheffield, United Kingdom; ^3^Department of Linguistics and English Language, University of Manchester, Manchester, United Kingdom; ^4^Department of Geography, University of South Carolina, Columbia, SC, United States

**Keywords:** dialectology, social media, Twitter, British English, big data, lexical variation, spatial analysis, sociolinguistics

## Abstract

There is a growing trend in regional dialectology to analyse large corpora of social media data, but it is unclear if the results of these studies can be generalized to language as a whole. To assess the generalizability of Twitter dialect maps, this paper presents the first systematic comparison of regional lexical variation in Twitter corpora and traditional survey data. We compare the regional patterns found in 139 lexical dialect maps based on a 1.8 billion word corpus of geolocated UK Twitter data and the BBC Voices dialect survey. A spatial analysis of these 139 map pairs finds a broad alignment between these two data sources, offering evidence that both approaches to data collection allow for the same basic underlying regional patterns to be identified. We argue that these results license the use of Twitter corpora for general inquiries into regional lexical variation and change.

## Introduction

Regional dialectology has traditionally been based on data elicited through surveys and interviews, but in recent years there has been growing interest in mapping linguistic variation through the analysis of very large corpora of natural language collected online. Such corpus-based approaches to the study of language variation and change are becoming increasingly common across sociolinguistics (Nguyen et al., 2016), but have been adopted most enthusiastically in dialectology, where traditional forms of data collection are so onerous. Dialect surveys typically require fieldworkers to interview many informants from across a region and are thus some of the most expensive and complex endeavors in linguistics. As a result, there have only been a handful of surveys completed in the UK and the US in over a century of research. These studies have been immensely informative and influential, shaping our understanding of the mechanisms of language variation and change and giving rise to the modern field of sociolinguistics, but they have not allowed regional dialect variation to be fully understood, especially above the levels of phonetics and phonology. As was recently lamented in the popular press (Sheidlower, 2018), this shift from dialectology as a social science to a data science has led to a less personal form of scholarship, but it has nevertheless reinvigorated the field, democratizing dialectology by allowing anyone to analyse regional linguistic variation on a large scale.

The main challenge associated with corpus-based dialectology is sampling natural language in sufficient quantities from across a region of interest to permit meaningful analyses to be conducted. The rise of corpus-based dialectology has only become possible with the rise of computer-mediated communication, which deposits massive amounts of regionalized language data online every day. Aside from early studies based on corpora of letters to the editor downloaded from newspaper websites (e.g., Grieve, 2009), this research has been almost entirely based on Twitter, which facilitates the collection of large amounts of geolocated data. Research on regional lexical variation on American Twitter has been especially active (e.g., Eisenstein et al., 2012, 2014; Cook et al., 2014; Doyle, 2014; Jones, 2015; Huang et al., 2016; Kulkarni et al., 2016; Grieve et al., 2018). For example, Huang et al. (2016) found that regional dialect patterns on American Twitter largely align with traditional dialect regions, based on an analysis of lexical alternations, while Grieve et al. (2018) identified five main regional patterns of lexical innovation through an analysis of the relative frequencies of emerging words. Twitter has also been used to study more specific varieties of American English. For example, Jones (2015) analyzed regional variation in African American Twitter, finding that African American dialect regions reflect the pathways taken by African Americans as they migrated north during the Great Migration. There has been considerably less Twitter-based dialectology for British English. Most notably, Bailey (2015, 2016) compiled a corpus of UK Twitter and mapped a selection of lexical and phonetic variables, while Shoemark et al. (2017) looked at a Twitter corpus to see if users were more likely to use Scottish forms when tweeting on Scottish topics. In addition, Durham (2016) used a corpus of Welsh English Twitter to examine attitudes toward accents in Wales, and Willis et al. (2018) have begun to map grammatical variation in the UK.

Research in corpus-based dialectology has grown dramatically in recent years, but there are still a number of basic questions that have yet to be fully addressed. Perhaps the most important of these is whether the maps of individual features generated through the analysis of Twitter corpora correspond to the maps generated through the analysis of traditional survey data. Some studies have begun to investigate this issue. For example, Cook et al. (2014) found that lexical Twitter maps often match the maps in the *Dictionary of American Regional English* and *Urban Dictionary* (see also Rahimi et al., 2017), while Doyle (2014) found that Twitter maps are similar to the maps from the *Atlas of North American English* and the *Harvard Dialect Survey*. Similarly, Bailey (2015, 2016) found a general alignment for a selection of features for British English. While these studies have shown that Twitter maps can align with traditional dialect maps, the comparisons have been limited—based on some combination of a small number of hand selected forms, restricted comparison data (e.g., dictionary entries), small or problematically sampled Twitter corpora (e.g., compiled by searching for individual words), and informal approaches to map comparison.

A feature-by-feature comparison of Twitter maps and survey maps is needed because it is unclear to what extent Twitter maps reflect general patterns of regional linguistic variation. The careful analysis of a large and representative Twitter corpus is sufficient to map regional patterns on Twitter, but it is also important to know if such maps generalize past this variety, as this would license the use of Twitter data for general investigations of regional linguistic variation and change, as well as for a wide range of applications. The primary goal of this study is therefore to compare lexical dialect maps based on Twitter corpora and survey data so as to assess the degree to which these two approaches to data collection yield comparable results. We do not assume that the results of surveys generalize; rather, we believe that alignment between these two very different sources of dialect data would be strong evidence that both approaches to data collection allow for more general patterns of regional dialect variation to be mapped. A secondary goal of this study is to test how consistent dialect patterns are across different communicative contexts. Corpus-based dialectology has shown that regional variation pervades language, even in the written standard (Grieve, 2016), but we do not know how stable regional variation is on the level of individual linguistic features. To address these gaps in our understanding of regional linguistic variation, this paper presents the first systematic comparison of lexical dialect maps based on surveys and Twitter corpora. Specifically, we report the results of a spatial comparison of the maps for 139 lexical variants based on a multi-billion-word corpus of geocoded British Twitter data and the BBC Voices dialect survey.

## British Dialectology

Interest in regional dialect variation in Great Britain is longstanding, with the earliest recorded comments on accent dating back to the fifteenth and sixteenth centuries (Trevisa, 1495). The study of regional variation in lexis grew in popularity during the late eighteenth and early nineteenth centuries, with dialect glossaries being compiled across the country, especially in Yorkshire and the North, in order to preserve local lexis, which was assumed to be going extinct. Most notably, Wright's (1898) *English Dialect Dictionary*, which drew on many of these glossaries, detailed lexical variation across the British Isles, especially England. The earliest systematic studies of accents in England also began around this time (see Maguire, 2012).

It was not until the *Survey of English Dialects* (SED) (Orton, 1962), however, that a full survey of dialect variation across England was attempted. Data was collected between 1950 and 1961 in 313 primarily rural locations using a 1,322 question survey, which included 730 lexical questions. Respondents, typically older males who had lived most of their lives in that location, were interviewed face-to-face by a fieldworker. The rest of the UK was covered separately. Scotland and Northern Ireland, along with the far north of England, were mapped by *The Linguistic Survey of Scotland*, which began collecting data in 1952 through a postal questionnaire (Mather et al., 1975). This survey also mapped regional variation in Scottish Gaelic (O'Dochartaigh, 1994). Finally, both Welsh (Jones et al., 2000) and English (e.g., Parry, 1999) in Wales were mapped in the late twentieth century.

With the rise of sociolinguistics in the 1960s and 1970s, work on language variation and change in the UK shifted focus from regional patterns to social patterns, generally based on interviews with informants from a range of social backgrounds from a single location. Interest in regional dialects, however, began to re-emerge recently. Llamas (1999) developed the Survey of Regional English (SuRE) to collect data from across levels of linguistic analysis. A national survey was never conducted, but the SuRE method was adopted for research in individual locations, including by Llamas (2007) in Middlesbrough, Asprey (2007) in the Black Country, and Burbano-Elizondo (2008) in Sunderland. In addition, the lexical component of the SuRE system was adapted for a national survey conducted as part of the BBC Voices project (Elmes, 2013). BBC Voices was designed to provide a snapshot of modern language use in the UK and employed various methods for data collection, including group interviews (Robinson et al., 2013), an attitudinal questionnaire (Bishop et al., 2005), and a web-based survey to collect lexical data based on SuRE. This lexical data, discussed below, is the basis for the present study. It has previously been subjected to statistical analysis (Wieling et al., 2014), which found evidence for four dialect regions (Southern England, Northern England, Scotland, and Northeast Scotland) based on a multivariate analysis of the maps for the top 10 variants of each of the 38 alternations. In addition to the BBC Voices survey, three other UK dialect surveys have recently come online. In 2007, Bert Vaux initiated the Cambridge online survey of World Englishes, which collects data on 31 alternations of various types from across the world, including the UK. MacKenzie et al. (2015) collected data on 31 alternations of various types from across the UK, with the help of undergraduate Linguistics and English Language students at the University of Manchester. Finally Leemann et al. (2018) used a mobile phone app to collect data on 26 alternations, primarily related to pronunciation, from over 47,000 speakers from over 4,900 localities from across the UK.

There is also a long history of corpus-based research in British dialectology. Most research on Old and Middle British dialects is essentially corpus-based, as it relies on samples of historical writing (e.g., Brook, 1963), but more specifically dialect corpora were compiled to map regional patterns in contemporary British English in the 1970s and 1980s. The first was the 1 million word *Helsinki Corpus of British English Dialects* (Ihalainen et al., 1987), designed as a grammatical supplement to the SED. Informants were recorded in their home and encouraged to talk about any subject they pleased to elicit naturalistic speech. The second was the 2.5 million word *Freiburg Corpus of English Dialects*, which contains transcriptions of interviews with older informants telling their life stories to fieldworkers (see Anderwald, 2009; Szmrecsanyi, 2013). Because these datasets consist of transcriptions of interviews elicited from a small number of informants, they fall in between traditional dialect surveys and the large natural language corpora that are the focus of this study.

Despite this long tradition of research, relatively little is known about regional linguistic variation in contemporary British English, especially compared to American English and especially in regard to lexical and grammatical variation. In large part this is because so few researchers have yet to take advantage of the immense social media corpora that can now be compiled and whose popularity is driving dialectology around the world. In addition to comparing lexical variation in corpora and surveys, a secondary goal of this study is therefore to encourage the adoption of computational approaches in British dialectology.

## Materials and Methods

### BBC Voices Dataset

The regional dialect survey data we used for this study was drawn from the BBC Voices project (Upton, 2013)^1^. We chose this dataset, which was collected online between 2004 and 2007, not only because it is easily accessible, but because it is the most recent lexical dialect survey of British English and because it focuses on everyday concepts, whereas older surveys tended to focus on archaic words and rural concepts, which are rarely discussed on Twitter.

The BBC Voices survey collected ~734,000 responses from ~84,000 informants to 38 open-ended questions, each designed to elicit the variants of a lexical alternation. The criteria for the selection of these 38 questions is unclear. Some (e.g., what word do you use for *running water smaller than a stream*) had been included in previous surveys, whereas others (e.g., *young person in cheap trendy clothes and jewelery*) were seemingly intended to elicit emerging forms (i.e., *chav*). In addition, two questions (*male partner, female partner*) are associated with variants that are not generally interchangeable (e.g., *boyfriend/husband, girlfriend/wife*); we therefore excluded these questions from our final analysis. All informants did not respond to all questions. The most responses were provided for *drunk* (29,275) and the fewest for *to play (a game)* (9,897). Across all responses, 1,146 variants were provided, with the most for *drunk* (104) and the fewest for *mother* (10). For example, of the 18 variants supplied in the 11,272 responses to the *left-handed* question, *cack-handed* (4,101) and *left* (3,987) are most common, together accounting for 72% of responses.

The large number of variants associated with each alternation is problematic because if we considered the complete set, our comparison would be dominated by very uncommon forms, which cannot be mapped accurately. Consequently, we only considered the most common variants of each alternation. In doing so, however, we violated the *principle of accountability*, which requires all variants to be taken into consideration (Labov, 1972). Fortunately, this frequency distribution ensures that excluding less common variants, which contribute so few tokens, will have almost no effect on the proportions of the more common variants. We therefore only retained variants that were provided by at least 5% of respondents. We tested other cut-offs, but higher thresholds (e.g., 10%) resulted in variants with clear regional patterns being excluded, whereas lower thresholds (e.g., 1%) resulted in variants that are too infrequent to show patterns being included.

Not only is each alternation associated with multiple variants, but each variant is associated with multiple distinct orthographic forms. These are the specific answers provided by informants that were judged by the BBC Voices team to be closely related to that variant, including inflections, non-standard spellings, and multiword units. Across all responses, 45,573 distinct forms were provided (ignoring capitalization), with the most for *unattractive* (2,300) and the fewest for *a long seat* (285). For example, of the 4,101 *cack-handed* responses to the *left-handed* question, informants provided 142 distinct orthographic forms, including “cack handed” (1,833) and “cack-handed” (1,026), which account for 70% of all responses, with the 18 forms provided by at least 10 informants accounting for 95% of responses. Alternatively, there are 86 forms provided by one informant, including “kerhandit” and “cack handedEnter Word,” the latter form clearly representing a data entry error.

The large number of forms associated with each variant is also problematic, especially because many of the most uncommon forms are of unclear status. This includes not only data entry errors, but forms that are almost never used with the target meaning, such as “china” for *mate*, which comes from “china plate” in Cockney rhyming slang. Fortunately, the frequency distribution also allowed us to exclude less frequent forms from our analysis without affecting the regional patterns of more frequent variants. For each variant we only included forms that were returned by at least 50 informants.

At the end of this process, our final feature set includes 36 alternations (e.g., left-handed), associated with 139 variants (e.g., *cack-handed, left, cag-handed*), which in turn are associated with 291 distinct orthographic forms (e.g., *cack handed, cack-handed*, etc.). The complete set of alternations and variants is presented in Table 1. The complete set of forms are included in the Supplementary Materials. The number of variants per alternation ranges from 2 to 7, most with 4 variants; the number of forms per variant ranges from 1 to 12, most with 2 forms. Notably, there are 291 forms in our dataset, but only 288 unique forms, because 3 are linked to the variants of multiple alternations: “chuck” is associated with the *throw* and *heavy rain* alternations, “hot” with the *hot weather* and *attractive* alternations, and “pissed” with the *annoyed* and *drunk* alternations. This situation is problematic and points to a larger issue with polysemy (and homophony) in our feature set, which we return to later in this paper, but crucially because the proportional use of each variant is calculated relative to the frequency of the other variants of that alternation, the maps for these overlapping variants are distinct.

**Table 1:** Feature set.

	**Alternation**	**Total**	**Variants**
1	Hot	6	*Boiling, roasting, hot, baked, sweltered, sweating*
2	Cold	4	*Freezing, chilly, nippy, cold*
3	Tired	2	*Knackered, shattered*
4	Unwell	3	*Sick, poorly, ill*
5	Pleased	3	*Chuffed, happy, made up*
6	Annoyed	2	*Pissed off, angry*
7	To play a game	2	*Play, lake*
8	To play truant	5	*Skive, bunk, wag, play hookey, skip*
9	Throw	2	*Chuck, lob*
10	Hit hard	5	*Whack, smack, thump, wallop, belt*
11	Sleep	5	*Kip, sleep, snooze, nap, doze*
12	Drunk	2	*Pissed, wasted*
13	Pregnant	4	*Up the duff, pregnant, bun in the oven, expecting*
14	Left-handed	3	*Cack-handed, left, cag-handed*
15	Lacking money	4	*Skint, broke, poor, brassic*
16	Rich	5	*Loaded, minted, well off, rolling in it, rich*
17	Insane	5	*Mad, nuts, crazy, mental, bonkers*
18	Attractive	4	*Fit, gorgeous, pretty, hot*
19	Unattractive	2	*Ugly, minger*
20	Moody	4	*Mardy, grumpy, stroppy, moody*
21	Baby	7	*Baby, bairn, sprog, babby, kid, wean, little one*
22	Mother	5	*Mum, mam, mummy, ma, mom*
23	Grandmother	3	*Nanny, granny, grandma*
24	Grandfather	4	*Grandad, grandpa, grampa, pop*
25	Friend	4	*Mate, pal, friend, buddy*
26	Young person in cheap trendy clothes and jewelery	4	*Chav, townie, scally, ned*
27	Clothes	5	*Clothes, gear, clobber, togs, kit*
28	Trousers	5	*Trousers, pants, keks, jeans, trews*
29	Child's soft shoes worn for PE	4	*Plimsolls, pumps, daps, trainers*
30	Main room of house (with TV)	4	*Living room, lounge, sitting room, front room*
31	Long soft seat in the main room	3	*Sofa, settee, couch*
32	Toilet	4	*Loo, bog, toilet, lavatory*
33	Narrow walkway alongside buildings	4	*Alley, ginnel, pavement, path*
34	To rain lightly	3	*Drizzle, spit, shower*
35	To rain heavily	4	*Pour, piss, chuck, bucket*
36	Running water smaller than a river	4	*Stream, brook, burn beck*
		139	

After selecting these 139 variants, we extracted the regional data for each from the BBC Voices dataset, which provides the percentage of informants in 124 UK postal code areas who supplied each variant. For example, the *cack-handed* variant accounted for 4,101 out of the 11,272 responses for the *left-handed* alternation (36%), with a minimum of 0% of informants using this form in the Shetlands and a maximum of 100% of informants in Jersey. Notably, these two extreme postal code areas have the fewest respondents, leading to generally less reliable measurements for these areas. Most areas, however, are associated with far more informants and thus exhibit much more variability. For example, 96% of postal code areas are characterized by between 10 and 70% usage of this particular variant. There are also a very small number of missing data points in our BBC Voices dataset (48 out of 17,236 values), which occur in cases where no responses were provided by any informants in that postal code area for that question. Because this is a negligible amount of missing data and because it is distributed across many variants, we simply assigned the mean value for that variant across all locations to those locations. In addition, because the BBC Voices dataset provides percentages calculated based on the complete set of variants, whereas we are looking at only the most common variants, we recalculated the percentage for each variant in each postal code area based only on the variants selected for analysis. For example, in the Birmingham area, the overall percentages for *cack-handed* (32.3%), *left* (23.8%), and *cag-handed* (32%), which cumulatively account for 88.1% of responses, were recalculated as 36.7, 27, and 36.3%, respectively, which sum to 100%.

**Figure 1:**
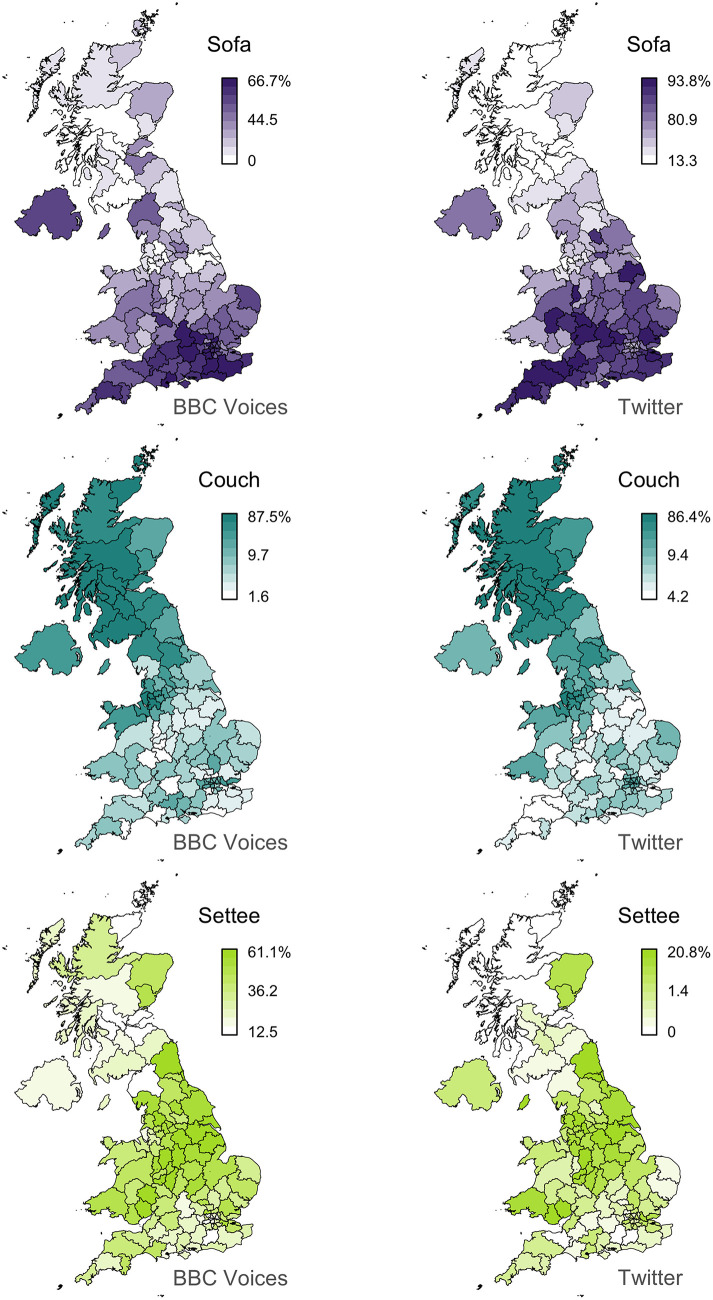
*Sofa/couch/settee* alternation.

Finally, we mapped each of the variants in this dataset. For example, the maps for the alternation between *sofa*/*couch*/*settee* is presented in the first column of Figure 1, where each map plots the percentage of one variant across the 124 postal code areas in the BBC Voices dataset. In this case, a clear regional pattern can be seen within and across variants, with *sofa* being relatively more common in the South, *couch* in Scotland, and *settee* in the Midlands and the North of England. The complete set of maps are presented in the Supplementary Materials.

### UK Twitter Dialect Corpus

The regional dialect corpus used for this study consists of a large collection of geolocated Twitter data from the UK that we downloaded between 2014-01-01 and 2014-12-31 using the Twitter API. This data was collected as part of a larger project that has explored lexical variation on Twitter (see also Huang et al., 2016; Grieve et al., 2017, 2018; Nini et al., 2017). In total, this corpus contains 1.8 billion words, consisting of 180 million Tweets, posted by 1.9 million unique accounts. The median number of Tweets per account is 10. The corpus contains data for 360 days, with data for 5 days missing due to technical issues. To analyse regional variation in the corpus, we formed regional sub-corpora by grouping all individual Tweets by postal code regions based on the provided longitude and latitude. Postal code regions were used to facilitate comparison with the BBC Voices data. Overall, the corpus contains 124 postal code regions, with on average 1.5 million Tweets per region, with the number of Tweets varying from between 5.5 million Tweets in Manchester to 54,000 Tweets in the Outer Hebrides, reflecting variation in population; London is not the largest region because it is subdivided into smaller areas.

Notably, we do not filter our corpus in any way, for example by excluding re-Tweets or spam or Tweets from prolific posters or bots. Tweets from one user may also appear in different regional sub-corpora if the user was in different postal code regions when those posts were made. The Twitter corpus analyzed in this study is an unbiased sample of geolocated Tweets, similar to what a user would see if they browsed Tweets from a region at random. We believe that modifying the corpus to make it more likely to show regional patterns is a highly subjective process that necessarily results in a less representative corpus. By including all Tweets from a given region in our corpus, we have taken a more conservative choice, allowing us to assess the base level of alignment between Twitter data and traditional dialect surveys. Removing Tweets from the corpus may lead to the identification of stronger regional patterns or better alignment with dialect survey maps, but this can only be tested once a baseline is established.

Next, we measured the frequency of each of the 139 lexical variants in our BBC Voices dataset across our 124 postal code area sub-corpora. We then summed the counts for all forms associated with each variant in each postal code area and computed a percentage for each variant for each alternation in each postal code area by dividing the frequency of that variant by the frequency of all variants of that alternation in that postal code area. In this way, we created a regional linguistic dataset based on our Twitter corpus that matches our BBC Voices dataset, consisting of percentages for all 139 variants, grouped into 36 alternations, measured across the 124 postal code areas, where the percentages for the variants for each alternation sum to 100% in each postal code area. We also mapped the percentages of all 139 variants across the 124 postal code areas. For example, the Twitter maps for the alternation between *sofa*/*couch*/*settee* are presented in the second column of Figure 1. The complete set of maps are presented in the Supplementary Materials.

Crucially, we counted all tokens of the variants in our corpus, making no attempt to disambiguate between word senses. For example, the variant *spit* in the alternation between *drizzle*/*spit* is used more often in the corpus to refer to the physical action as opposed to light rain, but we counted all tokens of *spit* regardless of the meaning it expressed. This is the simplest and most common approach in Twitter-based dialectology, although it is clearly problematic. Automatic word sense disambiguation systems are not commonly used in corpus-based dialectology because they are difficult to apply at scale and are fairly inaccurate, especially when working with uncommon dialect forms in highly informal data. We return to the issue of polysemy later in this paper, when we consider how variation in meaning affects the overall alignment between the two sets of maps and how much alignment can be improved through the application of techniques for word sense disambiguation.

Finally, it is important to acknowledge that Twitter corpora do not represent language in its entirety. Twitter corpora only represent Twitter, which is a very specific form of public, written, computer-mediated communication. The unique constellation of situational properties that define Twitter affect its form and differentiate it from other varieties of languages, as does the demographic background of Twitter users, who in the UK are more likely to be young, male, and well-educated compared to the general population (Longley et al., 2015; Mellon and Prosser, 2017). These are the social and situational patterns that define Twitter and they should be reflected in any corpus that attempts to represent this variety of language. The goal of this study is to evaluate the degree to which general patterns of regional variation persist in Twitter corpora despite its unique characteristics.

### Lee's *L*

To systematically assess the similarity of the Twitter maps and the survey maps we measured the degree of alignment between each pair of maps. There is, however, no standard method for bivariate map comparison in dialectology. Other than visually comparing dialect maps (e.g., Grieve et al., 2013), the simplest approach is to correlate the two maps by calculating a correlation coefficient (e.g., Pearson's *r*), essentially comparing the values of the two maps at every pair of locations. This was the approach taken in Grieve (2013), for example, where Pearson correlation coefficients were calculated to compare a small number of maps representing general regional patterns of grammatical and phonetic variation. This is also the general approach underlying many dialect studies that have used methods like factor analysis (e.g., Nerbonne, 2006) and principal components analysis (e.g., Shackleton, 2007) to identify common regional patterns in large sets of dialect maps based on correlation (or covariance) matrices. Although correlating dialect maps generally appears to yield consistent and meaningful results, this process ignores the spatial distribution of the values of each variable. Consequently, the similarity between two dialect maps can be estimated incorrectly and significance testing is unreliable, as it is based on the assumption that the values of a variable are independent across locations (see Lee, 2001).

Alternatively, methods in spatial analysis have been designed specifically for inferential bivariate map comparison (Wartenberg, 1985; Lee, 2001). Most notably, Lee (2001) proposed a spatial correlation coefficient (*L*) that measures the association between two geographically referenced variables, taking into account their spatial distribution. Lee's *L* is essentially a combination of Pearson's *r*, the standard bivariate measure of association, and Moran's *I*, the standard univariate measure of global spatial autocorrelation (see Grieve, 2018). On the one hand, Pearson's *r* correlates the values of two variables (*x* and *y*) by comparing the values of the variables at each pair of observations (i.e., locations) and can be expressed as

$$\begin{array}{l} r=\frac{\sum_{i}\left(x_{i}-\bar{x}\right)\left(y_{i}-\bar{y}\right)}{\sqrt{\sum_{i}{\left(x_{i}-\bar{x}\right)}^{2}}\sqrt{\sum_{i}{\left(y_{i}-\bar{y}\right)}^{2}}}\; \end{array}$$

On the other hand, Moran's *I* compares the values of a single variable (*x*) across all pairs of locations, with the spatial distribution of the variable used to define a spatial weights matrix (*w*), which specifies the weight assigned to the comparison of each pair of locations (*i, j*). For example, a spatial weights matrix is often set at 1 for neighboring locations and 0 for all other pairs of locations. When row standardized, Moran's *I* can be expressed as

$$\begin{array}{l} I=\frac{\sum_{i}\sum_{j}w_{ij}\left(x_{i}-\bar{x}\right)\left(x_{j}-\bar{x}\right)}{\sum_{i}{\left(x_{i}-\bar{x}\right)}^{2}}\; \end{array}$$

Combining these two measures, Lee defined his bivariate measure of spatial association *L* as

$$\begin{array}{l} L=\frac{\sum_{i}\left(\left(\sum_{j}w_{ij}\left(x_{j}-\bar{x}\right)\right)\left(\sum_{j}w_{ij}\left(y_{j}-\bar{y}\right)\right)\right)}{\sqrt{\sum_{i}{\left(x_{i}-\bar{x}\right)}^{2}\sum_{i}{\left(y_{i}-\bar{y}\right)}^{2}}}\; \end{array}$$

so that every pair of locations is compared within and across the two variables, taking into consideration the geographical distribution of the values. Like Pearson's *r*, Lee's *L* can range from −1 to +1, where stronger positive values indicate stronger matches. Lee's *L* is independent of scale, which is important as our maps can differ in terms of scale. In addition, pseudo-significance testing can be conducted for Lee's *L* through a randomization procedure, in much the same way as Moran's *I*. Lee's *L* is recalculated for a large number of random rearrangements of the locations over which the variable was measured. The set of values that results from this process represents the null distribution of Lee's *L*. The observed value of Lee's *L* is then compared to this null distribution to generate a pseudo *p*-value.

Finally, to calculate Lee's *L*, a spatial weights matrix must be defined. For this study, we used a nearest neighbor spatial weights matrix, where every location is compared to its nearest *n* neighbors, including itself, with each of these *n* neighbors assigned a weight of 1 and all other locations assigned a weight of 0. Following Grieve (2017), who suggests setting *n* at ~10% of the total locations, our main analysis is based on 10 nearest neighbors, calculated using postal code area centroids, but we also ran the analysis based on 2, 5, and 20 nearest neighbors, so as to judge how sensitive our results are to this setting.

## Results

### Map Comparison

We correlated all 139 pairs of Twitter and BBC Voices dialect maps using Lee's *L*, based on a 10 nearest neighbor spatial weights matrix. The 139 *L* values range from −0.28 to +0.74, with a median of +0.14, indicating a tendency for the maps to align. Overall, 109 of the 139 comparisons (78%) exhibit positive correlation coefficients, and 93 of these pairs (67%) exhibit significant correlations at the *p* < 0.05 level^2^. Computing Lee's *L* using 2, 5, and 20 nearest neighbors produced similar results, with all analyses finding that 78–80% of the map pairs exhibit positive correlations, and with the Lee's *L* values across all 139 pairs of maps exhibiting strong correlations (*r* > 0.89), indicating the choice of spatial weights matrix does not have a large effect on our results. We also computed Pearson correlation coefficients for all 139 pairs of maps, which yielded similar results (median *r* = 0.22, 82% of comparisons with positive correlations). Finally, there is a strong correlation between Pearson's *r* and Lee's *L* (*r* = 0.90), indicating that Lee's spatial adjustment does not have a large effect on our results.

These results demonstrate that the regional patterns in the BBC Voices survey data and our Twitter corpus are broadly comparable. It is unclear, however, just how similar these maps really are. Significant alignment, at any level, is not a guarantee of meaningful alignment. Furthermore, given standard rules of thumb for Pearson's *r*, a median Lee's *L* of 0.14 does not seem especially strong. We do not know, however, how exactly to interpret Lee's *L* within the context of this study. Ultimately, the question we are interested in answering is whether two sets of maps under comparison tend to align in a meaningful way for dialectologists. It is therefore crucial that we compare the two sets of maps visually to assess the degree of alignment, especially those map pairs that show seemingly low-to-middling correlations. In other words, we believe it is important to calibrate our interpretation of Lee's *L* for dialectological inquiry, rather than simply noting that a certain percentage of map pairs show a significant or substantial spatial correlation.

For example, we believe it is clear that the maps for *sofa, couch* and *settee* presented in Figure 1 broadly align. Lee's correlation coefficients here range between *L* = 0.63 for *couch*, which is the eighth best match in our dataset, to *L* = 0.27 for *settee*, which is the 40th best match. Crucially, the result for *settee* suggests that what appears to be low-to-middling values for Lee's *L* might represent very meaningful alignments in the context of dialectology. To investigate this issue further, we examined how the visual similarity between the 139 pairs of maps degrades as Lee's *L* falls.

**Figure 2:**
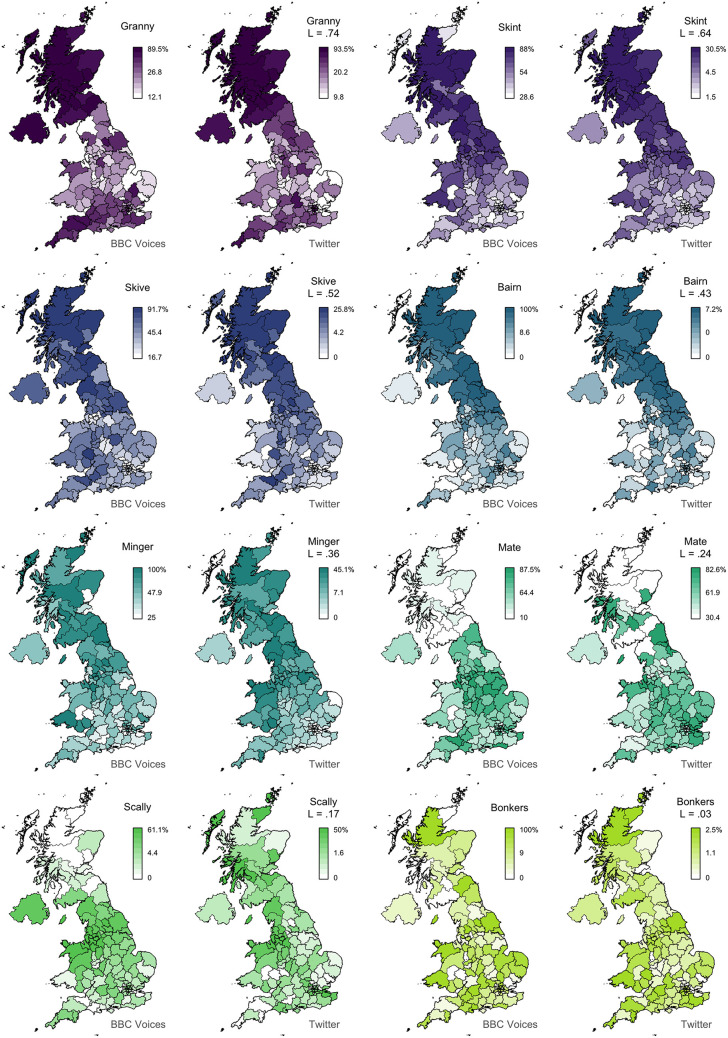
Map comparisons (part 1).

**Figure 3:**
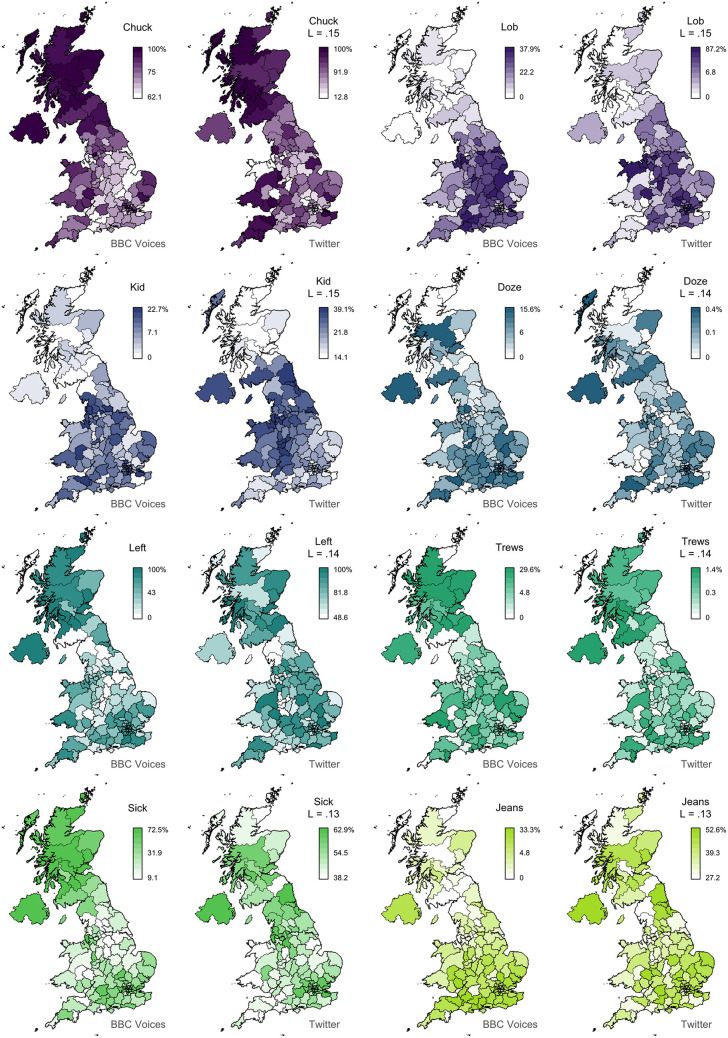
Map comparisons (part 2).

**Figure 4:**
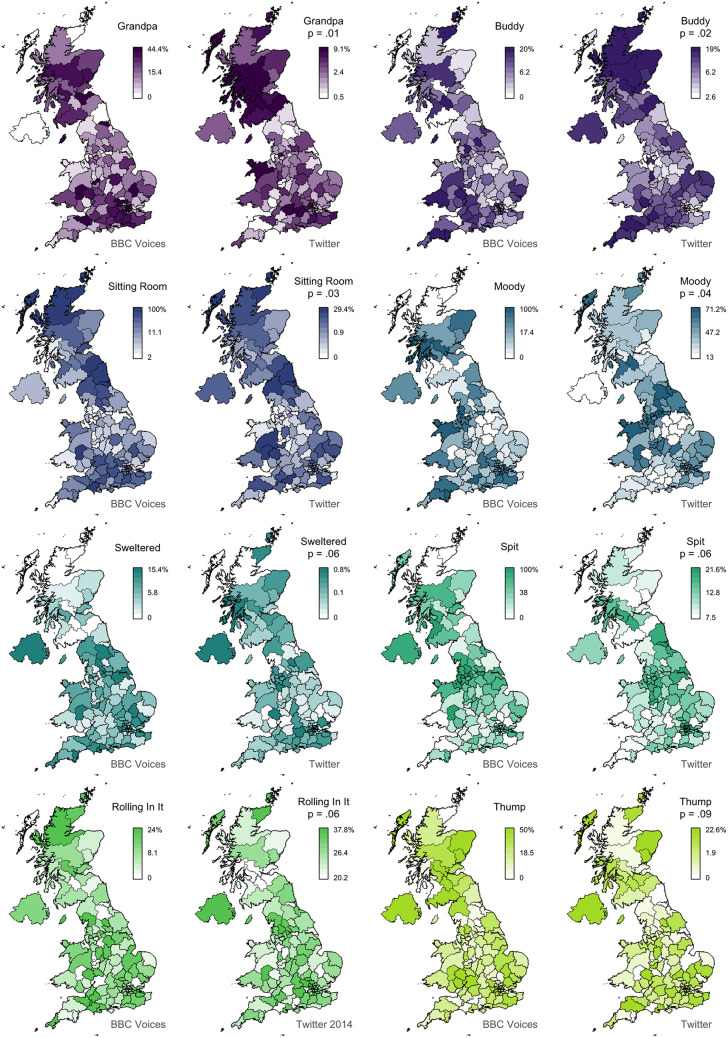
Map comparisons (part 3).

In Figure 2, we present 8 pairs of maps with *L* values ranging from 0.74 to 0.03. We can clearly see that the alignment between the two sets of maps falls with Lee's *L*, as expected. For example, the maps for *granny* (*L* = 0.74) show very similar patterns, identifying Scotland, Northern Ireland, and the Southwest as hotspots for this variant. The other three pairs of maps with *L* > 0.4 also appear to be very good matches. Below this level, we still find clear broad alignment between the maps, including for *mate* (*L* = 0.24), which is more common in England especially in the Midlands, and *scally* (*L* = 0.17), which is more common in the North, especially around Liverpool. Only *bonkers* (*L* = 0.04) shows no obvious alignment, but the two maps both show relatively little spatial clustering in the first place, and even these maps are not obviously inconsistent with each other. In Figure 3, we present 8 pairs of maps with *L* values around 0.14—the median Lee's *L* across all 139 maps. Once again, we see broad alignment across the maps, although there is considerably more local variation than most of the pairs of maps presented in Figure 2. For example, *chuck* (*L* = 0.15) is identified as occurring primarily outside England in both maps, but the Twitter map is less definitive and also identifies a hotspot in the Southwest. *Sick* (*L* = 0.13) probably shows the worst overall match across these 8 examples: both maps show the form is relatively common in Northern Ireland and the Southeast, but only the BBC Voices map also identifies Scotland as a hotspot. Finally, in Figure 4, we present 8 pairs of maps with *p* values around 0.05, all of which are associated with *L* values of <0.1. There is at least partial alignment between all pairs of maps associated with *p* < 0.05. For example, both maps identify *grandpa* (*L* = 0.06, *p* = 0.01) as occurring relatively more often in Scotland and the Home Counties, although the status of Northern Ireland and Wales is inconsistent. Even the maps for *spit* (*L* = 0.06, *p* = 0.06) align to some degree, with both identifying hotspots around Liverpool.

Overall, we therefore find considerable alignment between the BBC Voices and the Twitter lexical dialect maps. The matches are far from perfect, but in our opinion a clear majority of the map pairs analyzed in this study show real correspondence, with the nations of the UK and the major regions of England being generally classified similarly in both sets of maps. The maps do not appear to be suitable in most cases for more fine-grained interpretations, except at higher levels of correlation, but given that these maps are defined at the level of postal code areas, which in most cases are fairly large regions, this seems like a reasonable degree of alignment, suggesting that these two approaches to data collection in dialectology allow for similar broad underlying patterns of regional lexical variation to be identified in British English.

### Understanding Misalignments

Although the Twitter maps and the survey maps broadly correspond, the degree of alignment varies considerably across the 139 map pairs. To understand why some Twitter maps match the survey maps better than others, we considered how well alignment is predicted by three factors: the frequency of each variant in the Twitter corpus, the amount of spatial clustering in each Twitter map, and the likelihood of each variant occurring with the target meaning in the Twitter corpus. Knowing how these three characteristics of Twitter maps predict their alignment with survey maps not only offers guidance for improving the accuracy of Twitter maps, but it provides a basis for judging if new Twitter maps are likely to generalize, without comparison to survey maps, which are unavailable for most lexical alternations.

First, we included the frequency of each of the 139 variants in the complete Twitter corpus as a predictor in our model based on the assumption that measures of relatively frequency become better estimates of their true values as the number of tokens seen increases. Our intent was to assess how much misalignment can be explained by Twitter maps being based on too few observations. Second, we included the strength of the regional pattern exhibited by each of the 139 Twitter maps as a predictor in our model by computing the global spatial autocorrelation statistic Moran's *I* for each Twitter map using a 10 nearest neighbor spatial weights matrix. Our intent was to assess how much map misalignment can be explained by Twitter maps failing to exhibit clear regional patterns. It is important to acknowledge, however, that if the survey maps also fail to show regional patterns, misalignment should not be interpreted as evidence that the Twitter maps are inaccurate, as two random maps should not be expected to align. Furthermore, in general we expect these two measures to be correlated, as we know that Moran's *I* forms part of the foundation for Lee's *L*. Nevertheless, we wanted to assess how strong this relationship is, how much alignment increases with spatial clustering, and how much variation is left to be explained by other factors. Finally, we included an estimate of the percentage of tokens that were used with the target meaning in the corpus for each of the 139 variants as a predictor in our model by extracting 50 random concordance lines for each variant and coding them as target or non-target uses. Although polysemy is not an issue in surveys, where informants are asked to name concepts, variation in meaning should affect the accuracy of our Twitter maps, which were based on counts for all tokens of a variant regardless of their meaning. Our intent was to assess how much map misalignment is due to variation in the meaning of variants in the Twitter corpus.

We fit a linear mixed-effects regression model to Lee's *L*, measured across the 139 map pairs, with log-transformed frequency, Moran's *I*, and the percentage of target meaning as predictors, including alternation as a random intercept to account for the fact that the 139 variants are grouped into 36 alternations. Parameters were estimated using restricted maximum likelihood. Although Lee's *L* can range from −1 to +1, we used a linear model because the observed values range from −0.28 to +0.74 and because we are not focusing on the behavior of the model at extreme values. We log-transformed the frequency predictor because it is positively skewed, resulting in a clearer linear relationship with Lee's *L*.

The model is summarized in Table 2. All individual predictors in the fixed-effects component of our model are significant, while the variance component of our model indicates that a substantial amount of variability in Lee's *L* is attributable to variation across the 36 alternations. As expected, Moran's *I* and the percentage of target meanings are positively correlated with Lee's *L*, indicating that Twitter maps tend to be better matches when they show clear regional patterns and when they are primarily based on occurrences of word tokens with the target meaning. Frequency, however, is negatively associated with Lee's *L*, indicating that Twitter maps tend to be better matches when they are based on fewer tokens. This result is surprising. Although it suggests that our corpus is large enough to investigate this set of alternations, we believe that it also likely points to a fundamental issue with the ability of dialect surveys, as opposed to Twitter corpora, to map common words that are in use across the region of interest, often in alternation with less common regional words in the language of individuals. The relative usage of such words can still show continuous regional patterns, but it is difficult for such patterns to be mapped using surveys, where informants generally report one word per question. The drop in alignment as frequency rises may therefore reflect inaccuracies in the survey maps for common words, as opposed to the Twitter maps.

**Table 2:** Summary of the mixed-effects model fitted to Lee's *L*.

	**Parameter**	**Estimate**	**SE**	**Standardized estimate**
Fixed effects	Intercept	0.0412	0.0796	0.1648
	Moran's I	0.8172^***^	0.0845	0.1579
	Log-transformed frequency	−0.0250^**^	0.0075	−0.0532
	Target meaning ratio	0.0010^*^	0.0004	0.0357
Random effects	SD of random intercepts	0.1220		

* p < 0.05,

** p < 0.01,

*** *p < 0.001, p-values calculated using Satterthwaite's approximation*.

**Figure 5:**
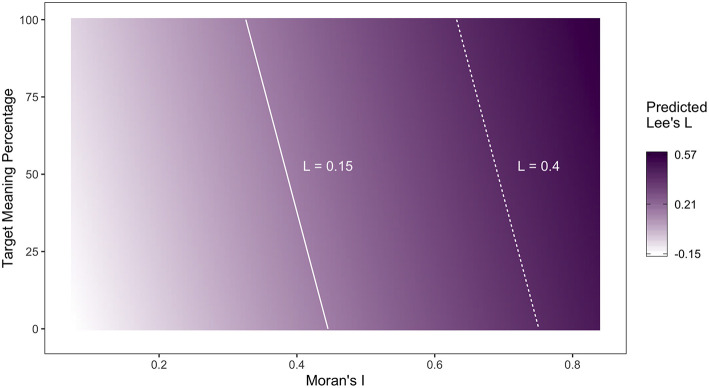
Expected value of Lee's *L* as a function of Moran's I and target meaning ratio.

Finally, we can use our model to propose some guidelines about how likely new Twitter maps are to generalize—without taking survey data, which is rarely available, into consideration. These guidelines are useful because they allow dialectologists who map regional lexical variation using Twitter corpora to assess how confident they should be that their maps identify general patterns. For example, if one is interested in mapping general dialect regions through the multivariate analysis of Twitter lexical alternation maps, these guidelines could be used to filter out maps that are less likely to generalize, prior to aggregation. Figure 5 illustrates how the expected value of Lee's *L* for map pairs changes as a function of the Moran's *I* and target token percentage, when log-transformed frequency takes its mean value. The solid and dashed lines represent cut-off values for Lee's *L* of 0.15 and 0.40 and were drawn to facilitate the assessment of the reliability of the alignment with a given combination of predictor values. For example, if we take a Lee's *L* value of 0.15 as being indicative of alignment, Twitter maps that have a Moran's *I* of at least 0.35 and are based on at least 50% target meanings can be expected to generalize.

### Dealing With Polysemy

As is common in Twitter dialect studies, we did not control for polysemy (and homophony). We found, however, that high levels of polysemy do affect the generalizability and presumably by extension the accuracy of these maps. To deal with this issue, methods for word sense disambiguation can be applied. At the most basic level, all the tokens of the relevant forms can be hand-coded. This is most accurate, but it is an extremely time-consuming task and thus usually impractical when working with large corpora or feature sets. Alternatively, various more advanced approaches could be applied. For example, a sample of tokens can be hand-coded and then a machine learning classifier can be trained on this data and used to code other tokens (Austen, 2017), or a token-based semantic vector space model could be applied (Hilpert and Saavedra, 2017). A simpler and more transparent approach is to only count tokens that occur in contexts where the target meaning is especially likely.

For example, as summarized in the first half of Table 3, the *playing truant* alternation, which includes 5 variants, shows considerable polysemy in our Twitter corpus, based on our hand coding of 50 random tokens of the form drawn from our corpus. Only *skive*, which is the variant with the best alignment, occurs with its target meaning over 50% of the time. The only other variant with a strong alignment is *bunk*, which remarkably occurs with its target meaning only 28% of the time, illustrating how a regional signal can be detected even when the target meaning is relatively rare. The other three variants, however, occur with their target meanings at most 10% of the time and show negative alignments, making them three of the worst matches in the feature set. Notably, the strength of alignment is clearly associated with the amount of spatial clustering, but there is no clear relationship with frequency. For example, *hookey*, which is the most infrequent variant, shows poor alignment, but so does *skip*, which is by far the most frequent variant.

**Table 3:** Descriptive statistics for the *playing truant* variants before and after filtering.

	**Variant**	***Corpus frequency***	***Spatial clustering*: Moran's *I***	***Polysemy*: percentage of target uses**	***Map alignment*: Lee's *L***
All tokens	*Bunk*	4757	0.39	28	0.47
	*Hookey*	808	0.10	10	−0.04
	*Skip*	28272	0.19	2	−0.13
	*Skive*	2666	0.54	82	0.52
	*Wag*	7549	0.21	0	−0.06
Filtered tokens	*Bunk*	559	0.49	100	0.57
	*Hookey*	41	0.11	100	0.07
	*Skip*	985	0.13	100	0.00
	*Skive*	547	0.38	100	0.39
	*Wag*	49	0.20	100	0.33

**Figure 6:**
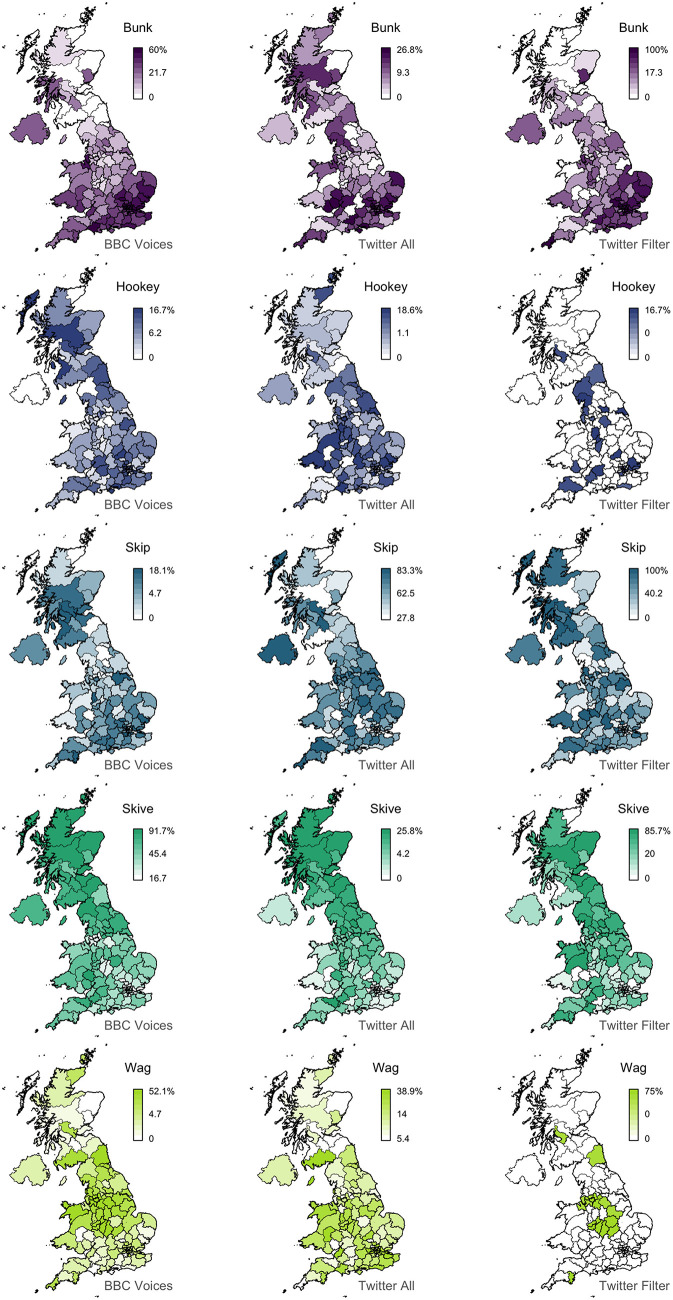
*Bunk/hookey/skip/skive/wag* alternation comparison.

To test whether we can improve the maps for this alternation through simple word-sense disambiguation we recounted these variants in the Twitter corpus in restricted contexts, identified based on concordance line analysis. Specifically, we only counted tokens of *skip* when it was immediately followed by *class, classes, college, lecture, school, uni, university*, or *work; bunk, skive*, and *wag* when followed by these words or *off*; and *hookey* when preceded by a form of the verb *play*. We then recomputed the variant percentages, as well as the three map characteristics used as predictors of our model. The results are presented in the second half of Table 3, while the variants in all three datasets are mapped in Figure 6.

Overall, there is a substantial rise in alignment after filtering: all three variants with negative correlations now show positive correlations, most notably *wag*. We also see a clear improvement in the alignment for *bunk*. Alternatively, although the alignment is still strong, we see a decrease for *skive*, presumably because the number of tokens examined has been drastically reduced, even though the vast majority of these tokens were used with the target meaning. This highlights the main limitation with word sense disambiguation: in most cases it will greatly reduce token counts, potentially down to problematic levels. For example, consider the maps for the rarest of these words: after filtering there are very few tokens left for *hookey* and *wag*, resulting in maps where most areas have no attestation at all of the form, suggesting that the corpus is too small to map these variants. Nevertheless, as the map for *wag* illustrates, such maps can still represent improvements over the unfiltered versions in terms of alignment with the survey data.

## Discussion

Although Twitter corpora are increasingly being used as the basis for dialect maps, their generalizability had not been established. Do these maps tell us anything about general patterns of regional variation, including in the spoken vernacular? Can these maps extend our general understanding of language variation and change? These are important questions because currently Twitter is the only data source from which precisely geolocated texts can be sampled at scale. Twitter maps have the potential to answer a range of basic questions in regional dialectology, but only if they are generalizable. In this study, we therefore set out to systematically test if Twitter maps, based on a 1.8 billion word corpus of geolocated Tweets collected in 2014 from across the UK, align with traditional survey maps, based on an unbiased sample of 139 lexical dialect maps taken from the BBC Voices dialect survey. Overall, we found broad correspondence between the two datasets, with a majority of the 139 map pairs showing meaningful levels of alignment in our opinion. In most cases, these two sets of maps agree across the four nations of the UK and within England between the North, the Midlands, and the South, although a substantial number of map pairs show more precise correspondence, for example identifying specific cities as hotspots for certain words. Given how different these two approaches to data collection are, we believe the alignment between these maps is strong evidence that Twitter maps are able to identify general dialect patterns.

The main outcome of this study is therefore validating the use of Twitter corpora for the analysis of general patterns of regional lexical variation, at least in British English. This is an important result for regional dialectology, because there are many advantages to working with dialect corpora as opposed to dialect surveys. Not only is it far easier to build corpora than conduct surveys, but dialect corpora allow for the open-ended analysis of a far wider range of features than surveys, which can only be used to collect data on a limited number of pre-selected features. Corpora also generally improve the resolution of dialect maps, allowing for more informants to be sampled over more locations. For example, our Twitter corpus contains posts from 1.9 million accounts, whereas the BBC Voices dataset contains responses from 84,000 informants. Finally, the fundamental reason to prefer dialect corpora is that they allow patterns of regional variation to be observed in natural language, whereas surveys only provide the opportunity to observe the linguistic opinion of informants, elicited in a single and very artificial communicative context.

**Figure 7:**
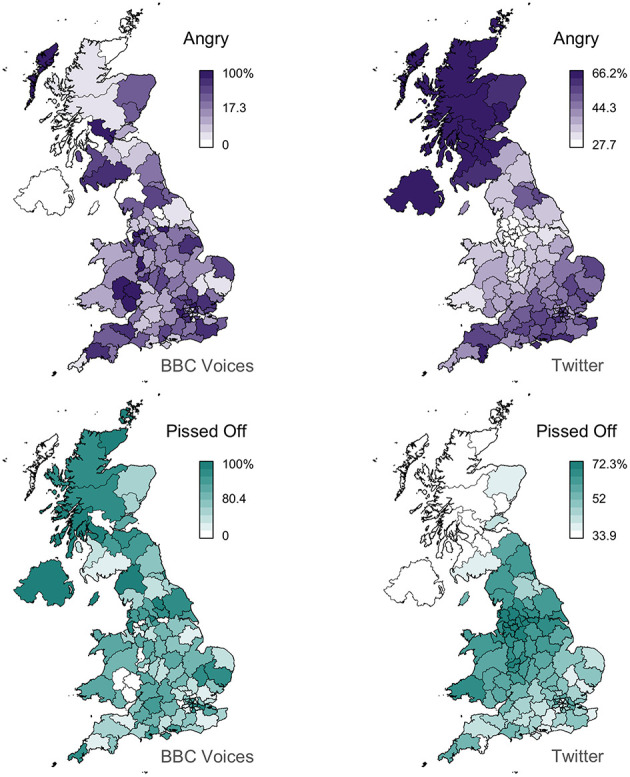
*Angry*/*pissed off* alternation.

For all these reasons, we believe that Twitter corpora can be the basis for general inquiry into regional lexical variation. However, we also believe that our analysis suggests that Twitter maps may generally provide a better foundation for dialectology than survey data, allowing for regional patterns to be identified more accurately in many cases. Perhaps the most striking example is the alternation between *angry* and *pissed off*, which is mapped in Figure 7. The Twitter maps identify much stronger regional patterns than the survey maps for these two variants, especially for *angry*, which shows limited spatial clustering in the survey data (Moran's *I* = 0.10), but a clear pattern in the Twitter data (Moran's *I* = 0.80). This example not only demonstrates how common words like *angry*, which are in usage across the UK, can show regional patterns and how these patterns can be identified through corpus analysis, but that such patterns can be difficult to access through surveys. This is reflected by the fact that the BBC Voices data for *angry* range from 0 to 100%, indicating that in some postal code areas no informant provided *angry*, whereas the Twitter analysis finds that in no postal code is either variant used <28% of the time. This result appears to expose a major limitation with standard survey-based approached to data collection in dialectology: individual informants can usually only supply a single variant per question, even when the informant uses multiple variants in their daily lives. In such cases, the maps for these variants, especially standard forms like *angry* that are clearly commonly used across the entire region of interest, may not accurately reflect patterns of regional linguistic variation in the population. The Twitter maps therefore seem to be more realistic than the survey maps, and by extension more reliable, although further research is necessary to directly test this hypothesis.

In addition to offering important validation for corpus-based approaches to regional dialectology, this study makes several other methodological contributions to the field. Perhaps of greatest value, we provide general quantitative guidelines for judging if Twitter-based maps are likely to generalize. We also introduce a new method for map comparison, Lee's *L*, which we borrowed from spatial analysis and which provides a more principled method for map correlation than approaches currently used in dialectology. We also show, however, that map comparison based on non-spatial correlation analysis yields similar results, offering support for the long tradition in dialectometry of using what are essentially correlation-based methods for aggregation (like Factor Analysis and Principal Components Analysis). Although we found Twitter maps to be remarkably robust in the face of polysemy, we also began to explore the use of techniques for word sense disambiguation to improve the reliability of lexical dialect maps; there is considerably more work to be done in this area. In addition, while we believe our results show that corpus-based approaches to dialectology are at least as powerful as survey-based approaches, our results also offer support for the generalisability of dialect surveys, whose validity has long been questioned, especially from outside the field (e.g., Pickford, 1956).

Descriptively, this study also presents one of the few corpus-based analyses of regional variation on the national level in modern British English. British dialectologists have not fully engaged with methods from computational sociolinguistics, and research has thus progressed slowly in recent years compared to American English. Consequently, there is much less agreement on issues such as the modern dialect regions of the UK than in the US, or how these regions are changing over time. These are the types of basic questions that British dialectologists can now pursue through the analysis of Twitter corpora, confident their results can provide insights about general patterns of regional linguistic variation in the UK.

Furthermore, our results not only offer evidence of the general value of Twitter corpora for theoretical research in dialectology, but they are themselves of direct relevance to our understanding of regional linguistic variation and change. Our main finding in this regard is that patterns of regional lexical variation are relatively stable across data sources—at least sufficiently stable for broad patterns of regional lexical variation to align. This result implies that patterns of regional lexical variation are relatively stable across communicative contexts. In fact, we find considerable evidence that the alternations behave quite differently in these two datasets: the median absolute difference in the maximum percentage of the 139 variants in the two datasets is 27%. In part, this is because of differences in how lexical alternation was measured, but the differences are so dramatic that it seems reasonable to assume that context matters in this regard. For example, the map for *bairn* (see Figure 2) shows that the variant is returned by up to 100% of informants in some areas the BBC Voices survey, but never accounts for more than 7% of the tokens of this alternation in any area in our Twitter corpus. Despite such differences in scale, these two maps show good alignment overall (*L* = 0.43). This result presumably obtains because the effect of situational variation is relatively consistent across the region: the percentage of *bairn* in the Twitter corpus drops dramatically, but the magnitude of this drop is relatively similar across the map, resulting in the same basic regional pattern being found in both datasets.

We believe this is an important result that sheds light on the relationship between the regional and situational determinants of language variation and change—an area that has been largely overlooked in dialectology and sociolinguistics, at least in part because dialect surveys and sociolinguistic interviews do not allow for situational variation to be analyzed in detail, as they involve eliciting data in one very specific and artificial context. Of course, there is still considerable disagreement between the two sets of maps, and our analysis of various characteristics of the Twitter maps only accounted for a proportion of this misalignment. Some of this variation may well be due to the interplay between region and situation. For example, it may be the case that people in different regions are using Twitter for a quantitatively different range of communicative purposes. Further research is necessary to explore these relationships, including analyzing and comparing regional variation in corpora representing other varieties of natural language, which will increasingly become possible as more and more language data comes online. However, much of this misalignment may also be due to social factors, which we have not considered in this study. In particular, we know that the demographics of our Twitter corpora do not match the demographics of the general population or presumably of the informants who responded to the BBC Voices survey. Similarly, some of this misalignment may be explained by our choice not to filter our Twitter dataset, for example by removing re-tweets. Our goal here was to evaluate the baseline level of alignment between Twitter dialect corpora and dialect surveys. How this alignment can be improved through more complex approaches to corpus construction could be the focus of future research now that we have set a baseline level of alignment.

Unfortunately, the analysis of social variation in Twitter is nowhere near as straightforward as the analysis of regional variation at this time, as the requisite metadata is not recorded or provided by Twitter or other social media platforms. Increasingly, however, researchers are developing powerful methods for estimating the demographics of Twitter users, based on a wide range of factors (e.g., Wang et al., 2019). Furthermore, there can be little doubt that as more and more of our lives are played out online increasing amounts of detailed social metadata will become available to researchers, as well as increasing amount of language data from across a wide range of registers, including the spoken vernacular. This will transform how we conduct sociolinguistic research. To truly understand how language variation and change functions as a system, across region, society, and communicative contexts, we must adopt a corpus-based approach to data collection. This is the only way that variation can be observed in a wide range of linguistic variables across a wide range of social and situational contexts. This is the promise of computational sociolinguistics and the future of our field.

## Data Availability

All datasets and code used for this study are included in the manuscript and/or the Supplementary Files.

## Author Contributions

JG, CM, and AN contributed conception and design of the study and wrote the first draft of the manuscript. DG, CM, JG, and AN organized the database. JG, AM, and AN conducted statistical analysis. JG, CM, AN, and AM wrote sections of the manuscript. All authors contributed to manuscript revision, read, and approved the submitted version.

### Conflict of Interest Statement

The authors declare that the research was conducted in the absence of any commercial or financial relationships that could be construed as a potential conflict of interest.

## Footnotes

^1^We downloaded the BBC Voices survey dataset, specifically the ‘RADAR 1' component of the dataset, from the project website, which is available at http://routledgetextbooks.com/textbooks/_author/upton-9780415694438

^2^We did not adjust the *p*-value for multiple comparisons because our goal is not to identify individual pairs of maps that show significant correlations. Rather, we are interested in reporting the proportion of the 139 map pairs that show a meaningful level of correlation in the context of dialectological inquiry, which is a much stricter test of robustness.

## References

[B1] Anderwald L. (2009). The Morphology of English Dialects: Verb-Formation in Non-standard English. Cambridge, UK: Cambridge University Press.

[B2] Asprey E. (2007). Black Country English and Black Country Identity (Unpublished PhD thesis). University of Leeds.

[B3] Austen M. (2017). Put the groceries up”: comparing black and white regional variation. Am. Speech 92, 298–320. 10.1215/00031283-4312064

[B4] Bailey G. (2015). Orthographic reflections of (ing): a Twitter-based corpus study, Paper Presented at Manchester Forum in Linguistics (Manchester: University of Manchester).

[B5] Bailey G. (2016). Regional variation in 140 characters: mapping geospatial tweets, Paper Presented at Workshop on Using Twitter for Linguistic Research (Canterbury: University of Kent).

[B6] Bishop H. Coupland N. Garrett P. (2005). Conceptual accent evaluation: thirty years of accent prejudice in the UK. Acta Linguist. Hafniensia 37, 131–154. 10.1080/03740463.2005.10416087

[B7] Brook G. L. (1963). English Dialects. London: Deutsch.

[B8] Burbano-Elizondo L. (2008). Language variation and identity in Sunderland (Unpublished PhD thesis). University of Sheffield.

[B9] Cook P. Han B. Baldwin T. (2014). Statistical methods for identifying local dialectal terms from GPS-tagged documents. Dictionaries 35, 248–271. 10.1353/dic.2014.0020

[B10] Doyle G. (2014). Mapping dialectal variation by querying social media, in Proceedings of the 14th Conference of the European Chapter of the Association for Computational Linguistics, eds Wintner S. Goldwater S. Riezler S. (Gothenburg), 98–106.

[B11] Durham M. (2016). Changing attitudes towards the welsh english accent: a view from Twitter, in Sociolinguistics in Wales, eds Durham M. Morris J. (Basingstoke: Palgrave, 181–205.

[B12] Eisenstein J. O'Connor B. Smith N. A. Xing E. P. (2012). Mapping the geographical diffusion of new words. PLOS ONE 9.

[B13] Eisenstein J. O'Connor B. Smith N. A. Xing E. P. (2014). Diffusion of lexical change in social media. PLoS ONE 9:e113114. 10.1371/journal.pone.0113114 25409166 PMC4237389

[B14] Elmes S. (2013). Voices: a unique BBC adventure, in Analysing 21st Century British English: Conceptual and Methodological Aspects of the “Voices” Project, eds Upton C. Davies B. (London: Routledge, 1–11.

[B15] Grieve J. (2009). A Corpus-Based Regional Dialect Survey of Grammatical Variation in Written Standard American English (Ph.D. dissertation). Northern Arizona University.

[B16] Grieve J. (2013). A statistical comparison of regional phonetic and lexical variation in American English. Lit. Linguist. Comput. 28, 82–107. 10.1093/llc/fqs051

[B17] Grieve J. (2016). Regional Variation in Written American English. Cambridge, UK: Cambridge University Press.

[B18] Grieve J. (2017). Assessing smoothing parameters in dialectometry, in From Semantics to Dialectometry: Festschrift in Honor of John Nerbonne, eds Wieling M. Kroon M. van Noord G. Bouma G. (Tributes 32, College Publications), 119–126.

[B19] Grieve J. (2018). Spatial statistics for dialectology, in The Handbook of Dialectology, eds Boberg C. Nerbonne J. Watt D. (Oxford: Wiley-Blackwell), 415–433.

[B20] Grieve J. Asnaghi C. Ruette T. (2013). Site-restricted web searches for data collection in regional dialectology. Am. Speech 88, 413–440. 10.1215/00031283-2691424

[B21] Grieve J. Nini A. Guo D. (2017). Analyzing lexical emergence in Modern American English online. Engl. Lang. Linguist. 21, 99–127. 10.1017/S1360674316000113

[B22] Grieve J. Nini A. Guo D. (2018). Mapping lexical innovation on American social media. J. Engl. Linguist. 46, 293–319. 10.1177/0075424218793191

[B23] Hilpert M. Saavedra D. C. (2017). Using token-based semantic vector spaces for corpus-linguistic analyses: from practical applications to tests of theoretical. Corpus Linguist. Linguist. Theory. 1–32. 10.1515/cllt-2017-0009

[B24] Huang Y. Guo D. Kasakoff A. Grieve J. (2016). Understanding U.S. regional linguistic variation with Twitter data analysis. Comput. Environ. Urban Syst. 59, 244–255. 10.1016/j.compenvurbsys.2015.12.003

[B25] Ihalainen O. Kyto M. Rissanen M. (1987). The Helsinki corpus of english texts: diachronic and dialectal report on work in progress, in Corpus Linguistics and Beyond, Proceedings of the Seventh International Conference on English Language Research on Computerized Corpora. Amsterdam, 21–32.

[B26] Jones G. E. Robert O. J. Alan R. T. David T. (2000). The Welsh Dialect Survey. Cardiff: University of Wales Press.

[B27] Jones T. (2015). Toward a description of African American vernacular english dialect regions using “Black Twitter”. Am. Speech 90, 403–440. 10.1215/00031283-3442117

[B28] Kulkarni V. Perozzi B. Skiena S. (2016). Freshman or fresher? Quantifying the geographic variation of internet language, in Proceedings of the Tenth International AAAI Conference on Web and Social Media (ICWSM 2016), eds Strohmaier M. Gummadi K. P. (Palo Alto, CA: The AAAI Press, 615–618.

[B29] Labov W. (1972). Sociolinguistic Patterns. Philadelphia, PA: University of Philadelphia Press.

[B30] Lee S.-I. L. (2001). Developing a bivariate spatial association measure: an integration of Pearson's *r* and Moran's *I*. J. Geogr. Syst. 3, 369–385. 10.1007/s101090100064

[B31] Leemann A. Marie-José K. David B. (2018). The English Dialects App: the creation of a crowdsourced dialect corpus. Ampersand 5, 1–17. 10.1016/j.amper.2017.11.001

[B32] Llamas C. (1999). A new methodology: data elicitation for social and regional language variation studies. Leeds Work. Pap. Linguist. Phon. 7, 95–118.

[B33] Llamas C. (2007). A place between places”: language and identities in a border town. Lang. Soc. 36, 579–604. 10.1017/S0047404507070455

[B34] Longley P. A. Adnan M. Lansley G. (2015). The geotemporal demographics of Twitter usage. Environ. Plann. A 47, 465–484. 10.1068/a130122p

[B35] MacKenzie L. Bailey G. Danielle T. (2015). Our Dialects: Mapping Variation in English in the UK. Available online at: http://tiny.cc/OurDialects

[B36] Maguire W. (2012). Mapping the existing phonology of english dialects. Dialectol. Geolinguist. 20, 84–107. 10.1515/dialect-2012-0006

[B37] Mather J. Y. Speitel H. H. Leslie G. W. (1975). (eds.). *The Linguistic Atlas of Scotland, Scots Section, Vol. 1* Hamden, CT: Archon Books.

[B38] Mellon J. Prosser C. (2017). Twitter and Facebook are not representative of the general population: Political attitudes and demographics of British social media users. Res Polit. 4, 1–9. 10.1177/2053168017720008

[B39] Nerbonne J. (2006). Identifying linguistic structure in aggregate comparison. Lit. Linguist. Comput. 21, 463–475. 10.1093/llc/fql041

[B40] Nguyen D. Dogruöz A. S. Ros,é C. P. De Jong F. (2016). Computational sociolinguistics: a survey. Comput. Linguist. 42, 537–593. 10.1162/COLI_a_00258

[B41] Nini A. Corradini C. Guo D. Grieve J. (2017). The application of growth curve modeling for the analysis of diachronic corpora. Lang. Dyn. Change. 7, 102–125. 10.1163/22105832-00701001

[B42] O'Dochartaigh C. (1994). Survey of the Gaelic Dialects of Scotland: Questionnaire Materials Collected for the Linguistic Survey of Scotland. Dublin: Dublin Institute for Advanced Studies; School of Celtic Studies.

[B43] Orton H. (1962). Survey of English dialects: Introduction. Leeds: Arnold.

[B44] Parry D. (1999). A Grammar and Glossary of the Conservative Anglo-Welsh Dialects of Rural Wales. Sheffield: NATCECT.

[B45] Pickford G. R. (1956). American linguistic geography: a sociological appraisal. Word 12, 211–233. 10.1080/00437956.1956.11659600

[B46] Rahimi A. Cohn T. Baldwin T. (2017). A Neural model for user geolocation and lexical dialectology. arXiv. 209–216. 10.18653/v1/P17-2033

[B47] Robinson J. Herring J. Gilbert H. (2013). The British library description of the BBC voices recordings collection, in Analysing 21st Century British English: Conceptual and Methodological Aspects of the “Voices” Project, 1st Edn, eds Upton C. Davies B. (London; New York, NY: Routledge, 136–161.

[B48] Shackleton R. (2007). Phonetic variation in the traditional English dialects: a computational analysis. J. Engl. Linguist. 35, 30–102. 10.1177/0075424206297857

[B49] Sheidlower J. (2018). The Closing of a Great American Dialect Project. The New Yorker. Available online at: https://www.newyorker.com/culture/cultural-comment/the-closing-of-a-great-american-dialect-project (accessed September 22, 2017).

[B50] Shoemark P. Sur D. Shrimpton L. Murray I. Goldwater S. (2017). Aye or naw, whit dae ye hink? Scottish independence and linguistic identity on social media, in Proceedings of the 15th Conference of the European Chapter of the Association for Computational Linguistics: Volume 1, Long Papers, 1239–1248. (Valencia: Association for Computational Linguistics). 10.18653/v1/E17-1116

[B51] Szmrecsanyi B. (2013). Grammatical Variation in British English Dialects: A Study in Corpus-Based Dialectometry. Cambridge: Cambridge University Press.

[B52] Trevisa J (1495) Policronicon. Westminster: Wynkyn Theworde.

[B53] Upton C. (2013). Blurred boundaries: the dialect word from the BBC, in Analysing 21st Century British English: Conceptual and Methodological Aspects of the “Voices” Project, eds Upton C. Davies B. (London: Routledge, 180–197.

[B54] Wang Z. Hale S. Adelani D. I. Grabowicz P. Hartman T. Flack F. (2019). Demographic inference and representative population estimates from multilingual social media data, in Proceeding of WWW '19 The World Wide Web Conference (San Francisco, CA: ACM), 2056–2067.

[B55] Wartenberg D. (1985). Multivariate spatial correlation: a method for exploratory geographical analysis. Geogr. Anal. 17, 263–283. 10.1111/j.1538-4632.1985.tb00849.x

[B56] Wieling M. Upton C. Thompson A. (2014). Analyzing the BBC voices data: contemporary english dialect areas and their characteristic lexical variants. Lit. Linguist. Comput. 29, 107–117. 10.1093/llc/fqt009

[B57] Willis D. Leemann A. Gopal D. Blaxter T. (2018). Localising morphosyntactic variation in Welsh Twitter data, Presented at NWAV 47 (New York, NY).

[B58] Wright J. (ed.). (1898). The English Dialect Dictionary: A-C, Vol. 1. Oxford: Oxford University Press.

