# Supplementary figures and images for: Mapping Lexical Dialect Variation in British English Using Twitter
Source: Front Artif Intell. 2019 Jul 12;2:11. doi: 10.3389/frai.2019.00011 (PMC7861259; doi:10.3389/frai.2019.00011)

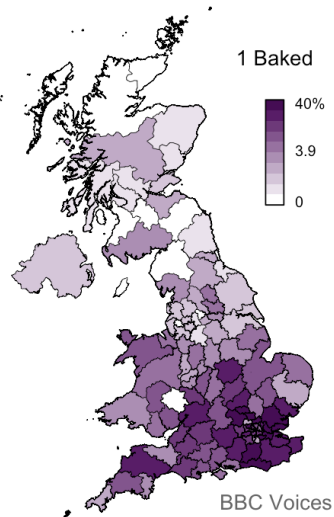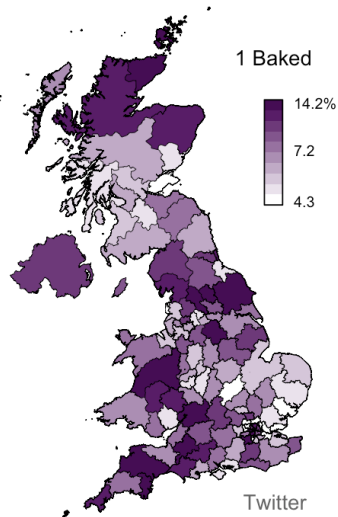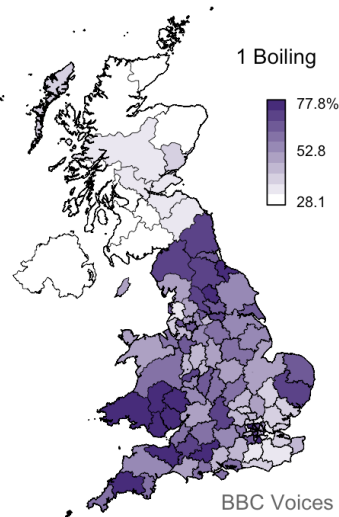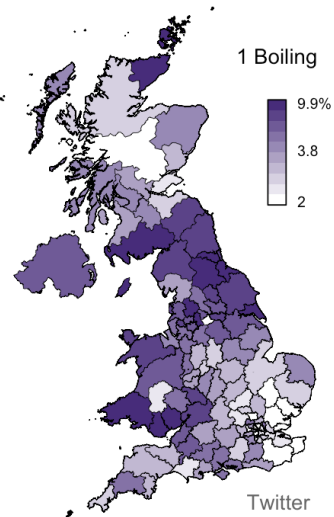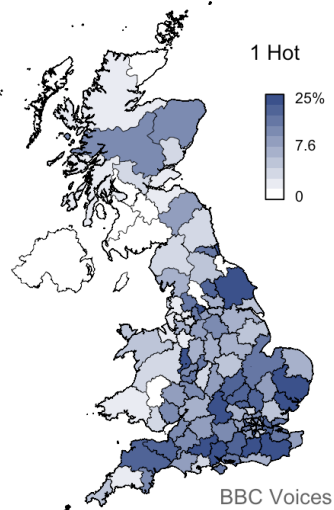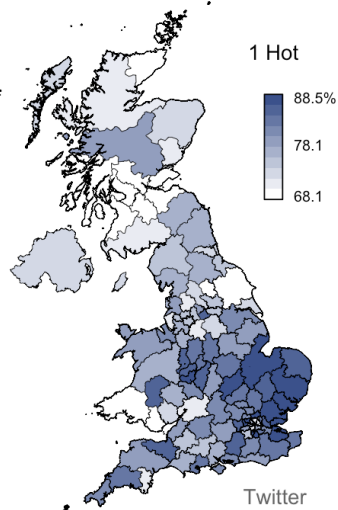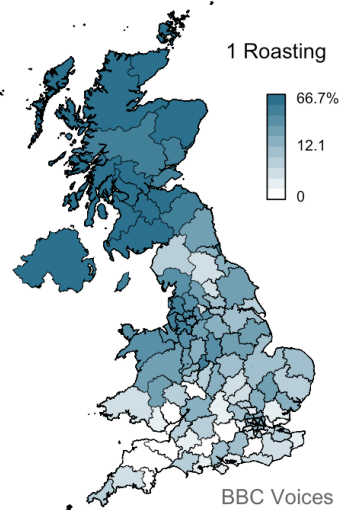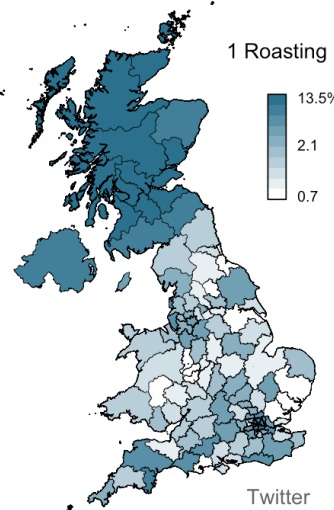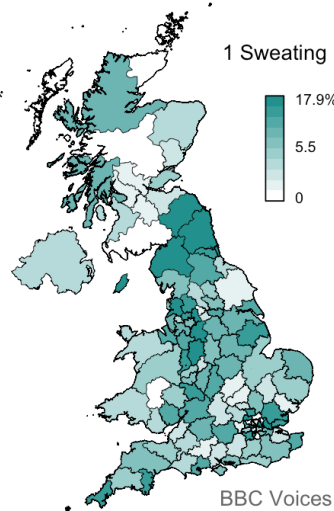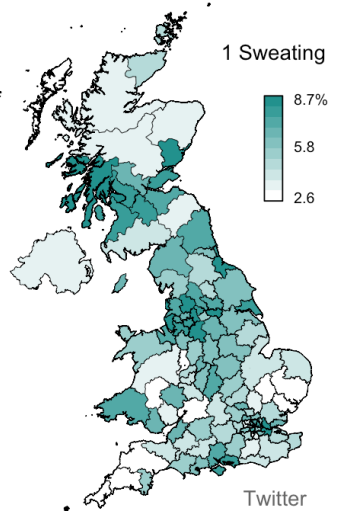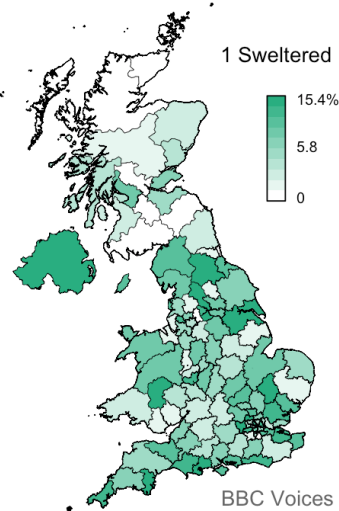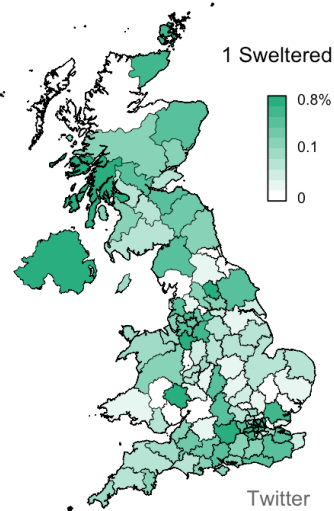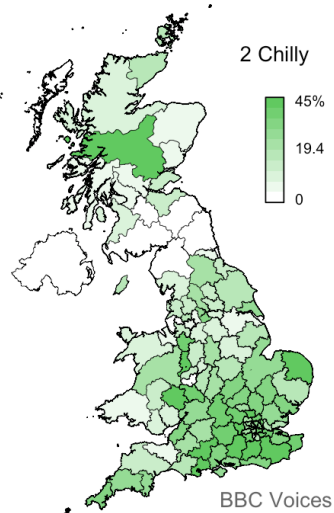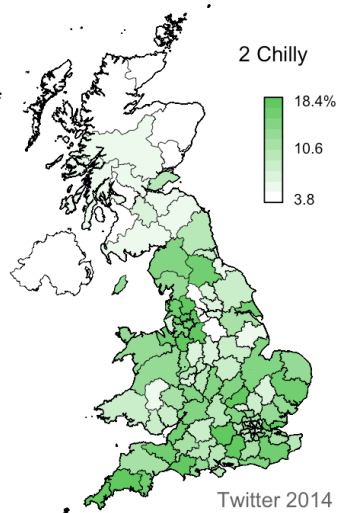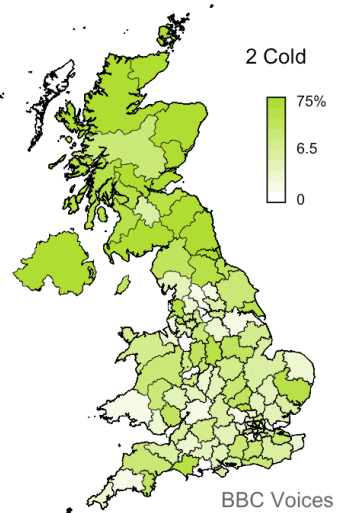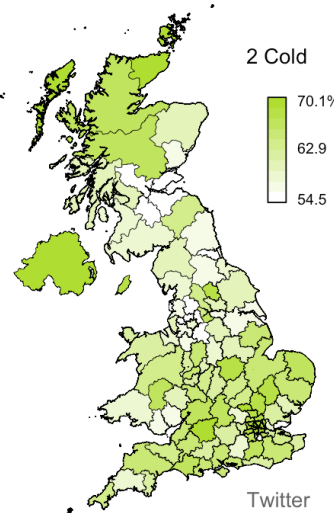

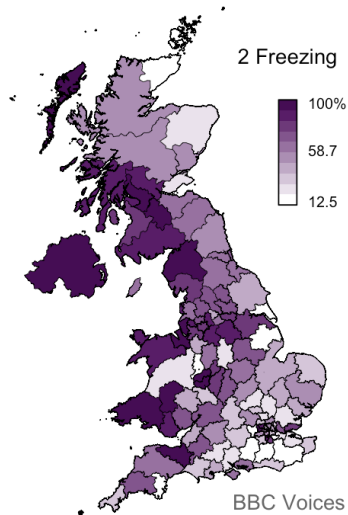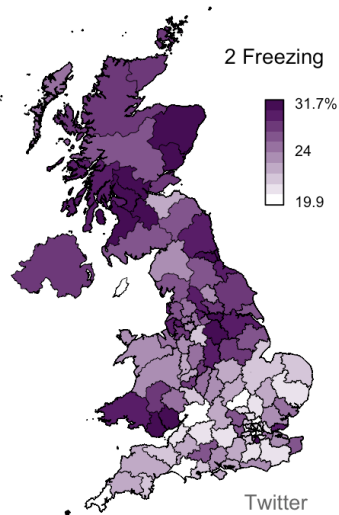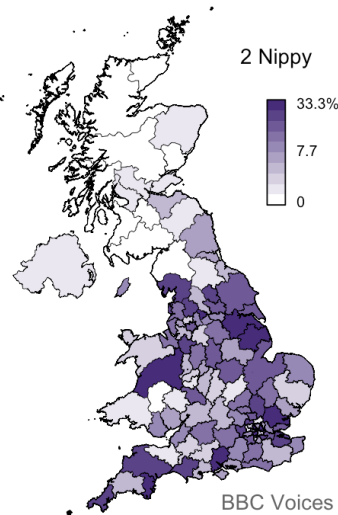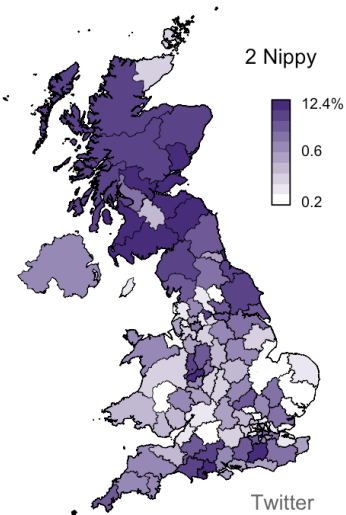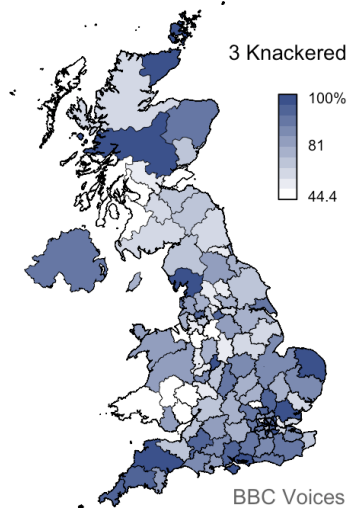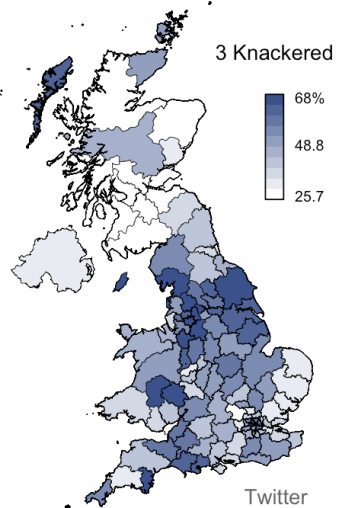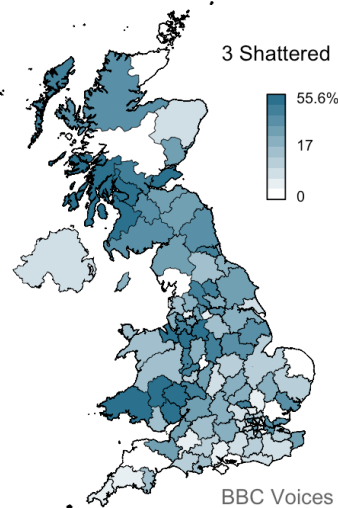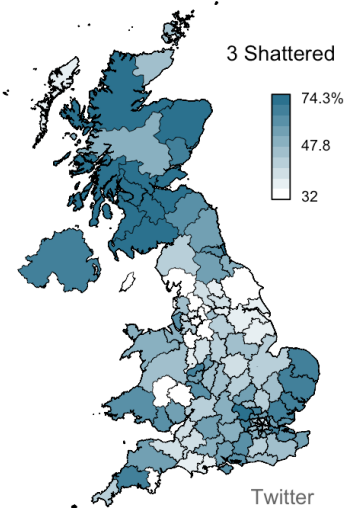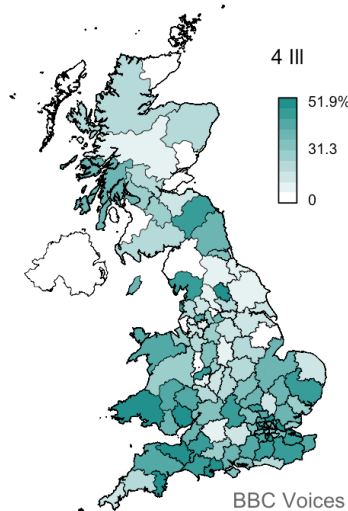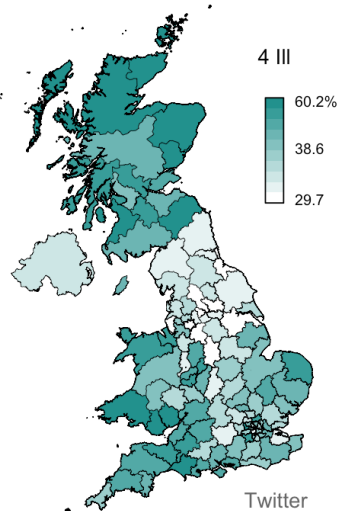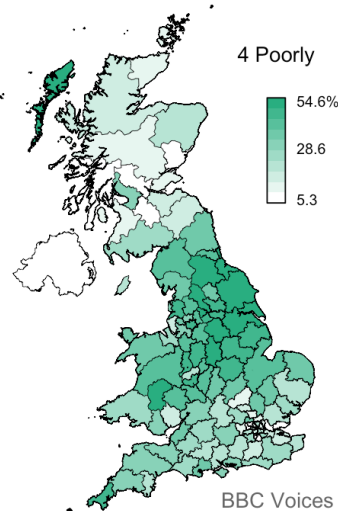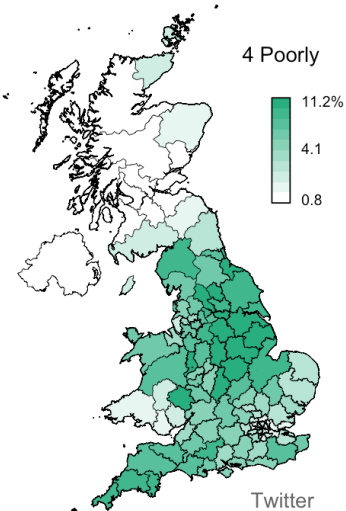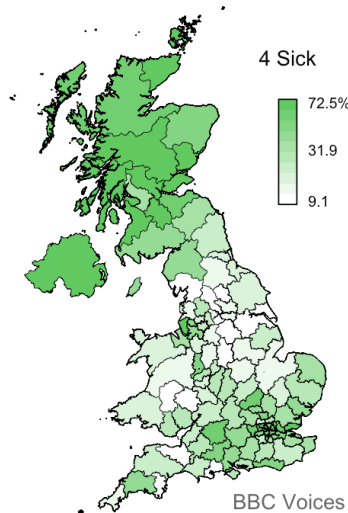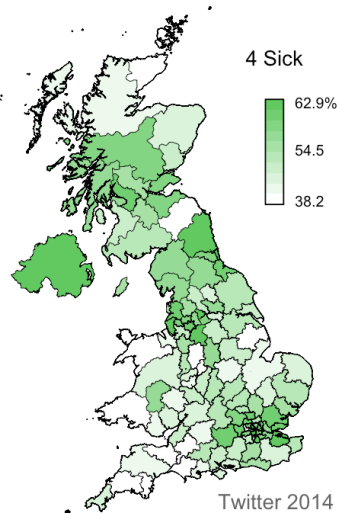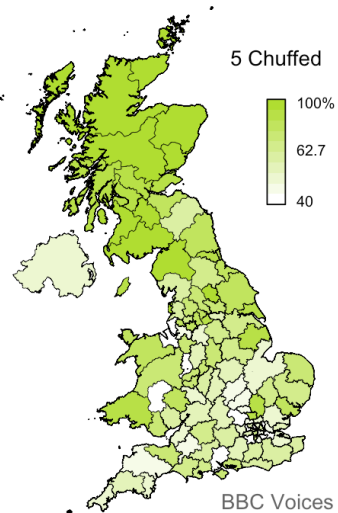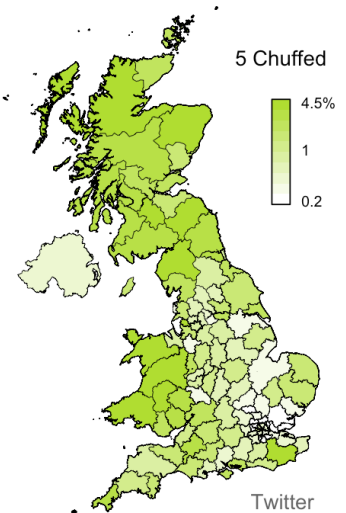

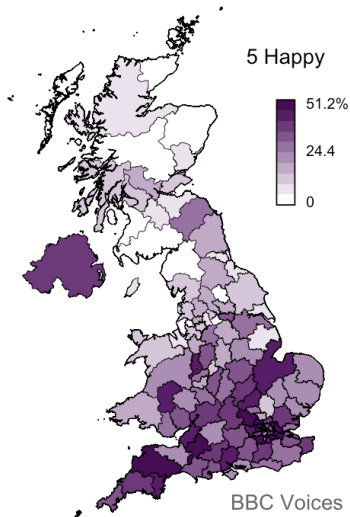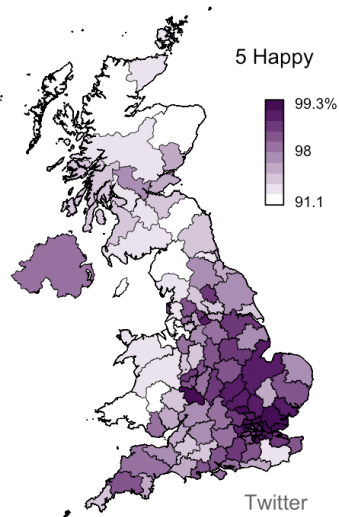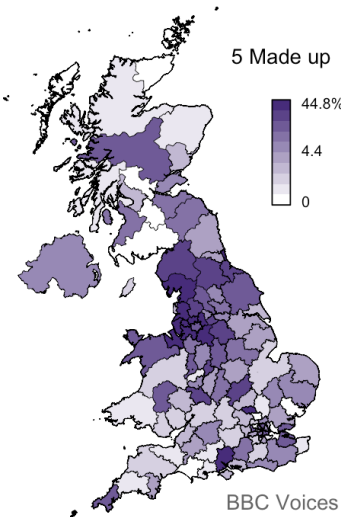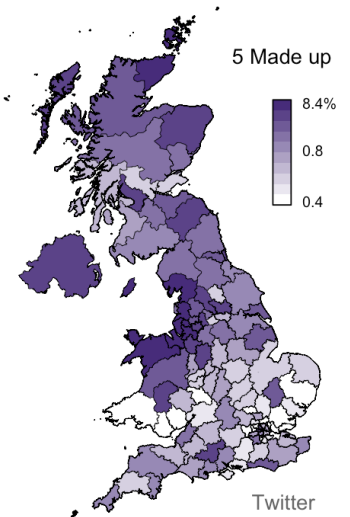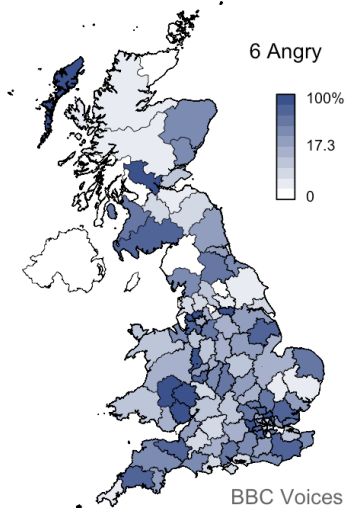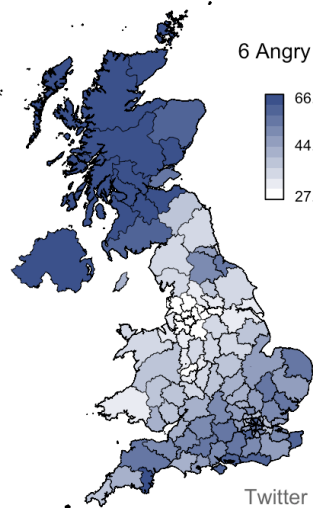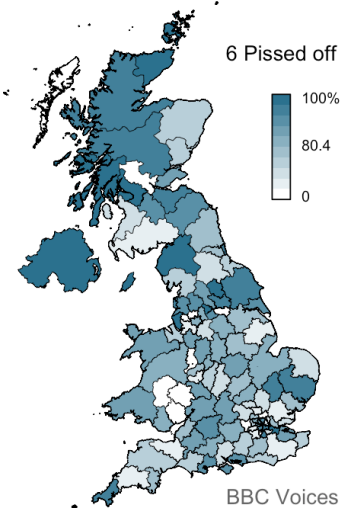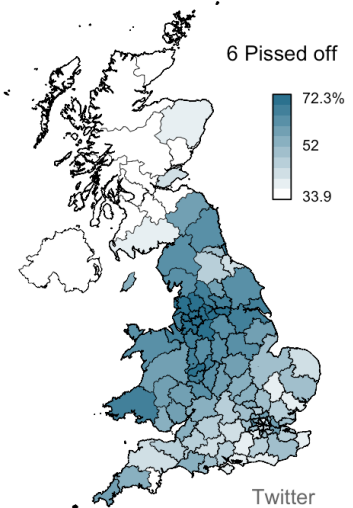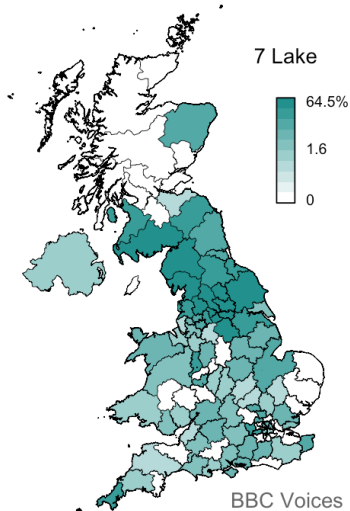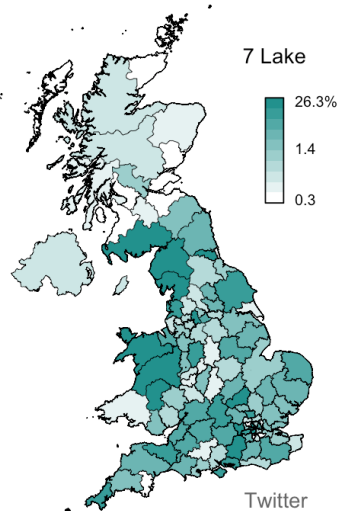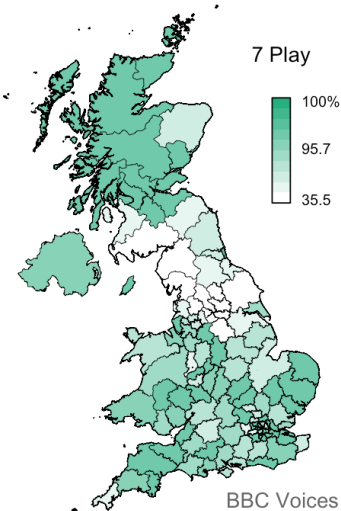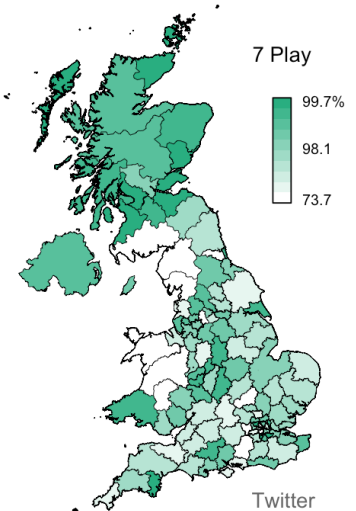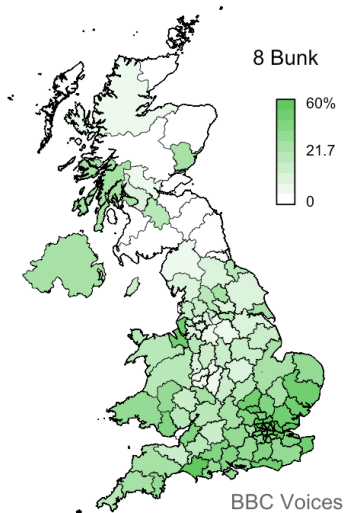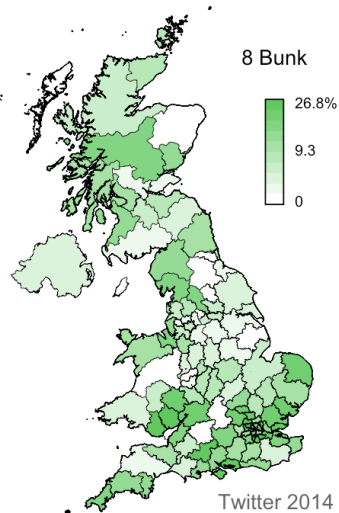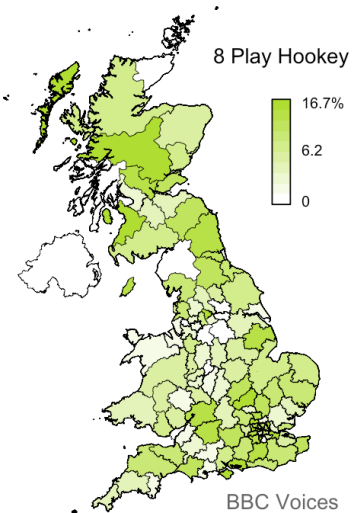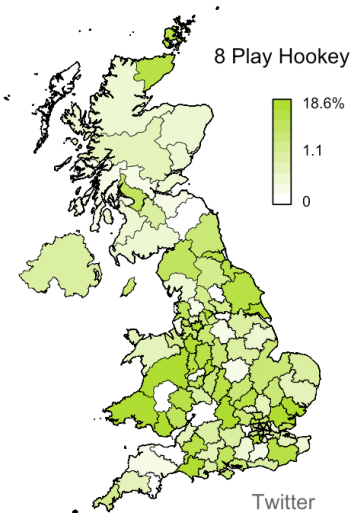

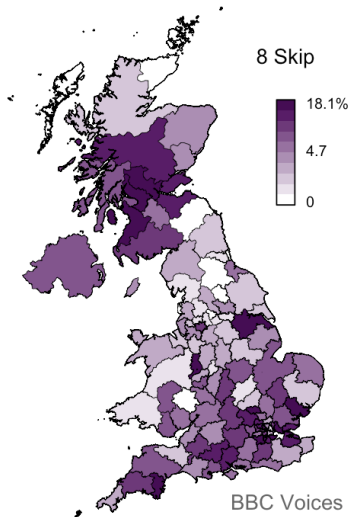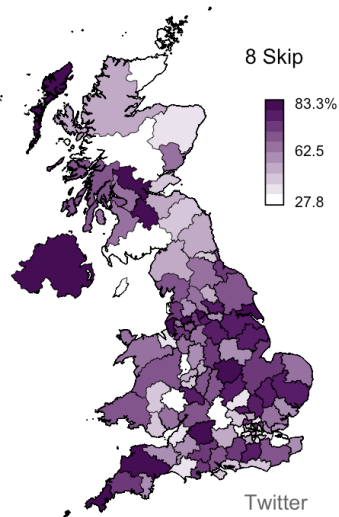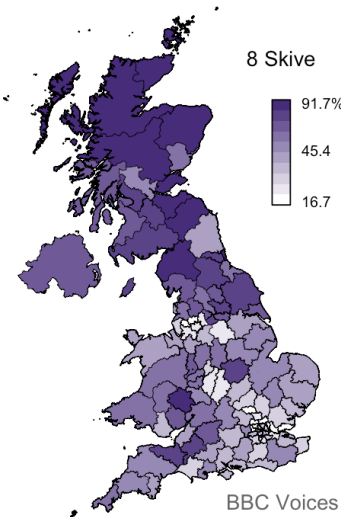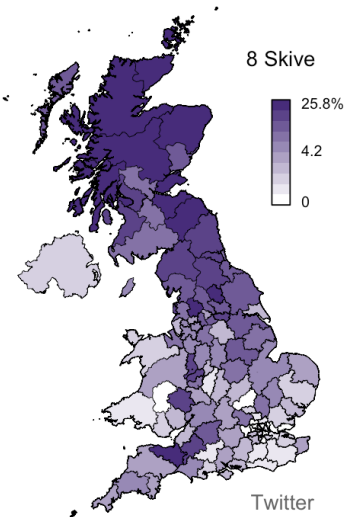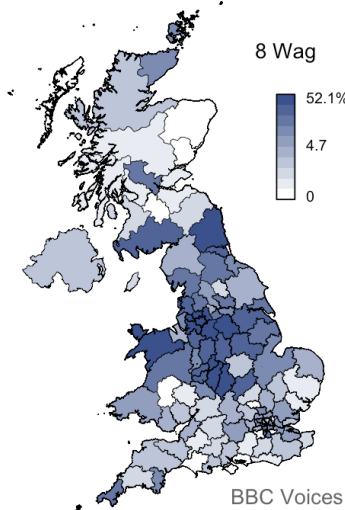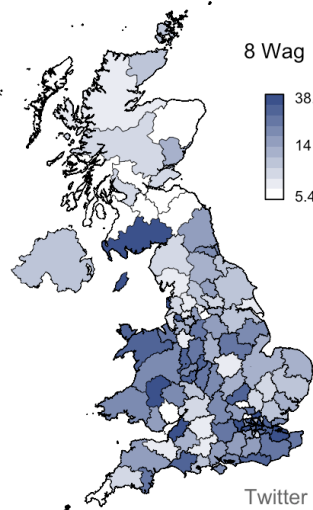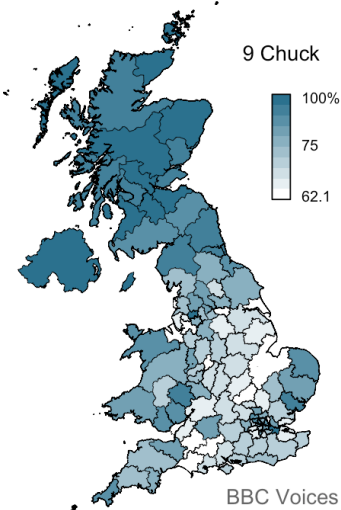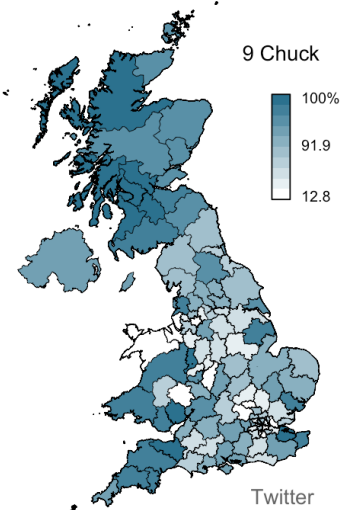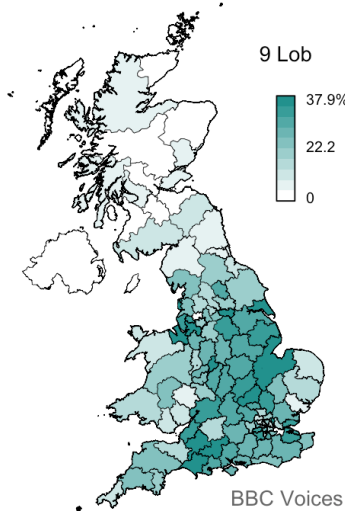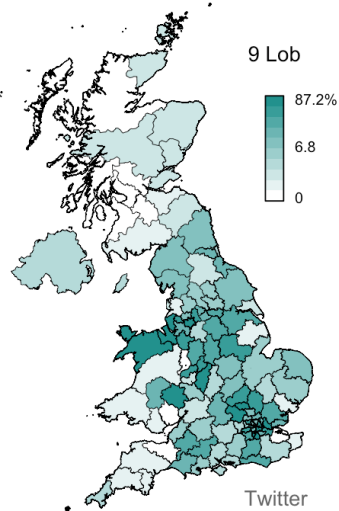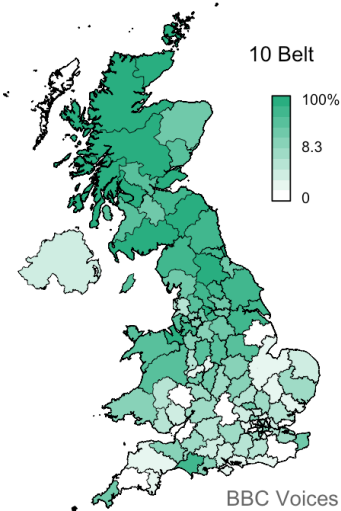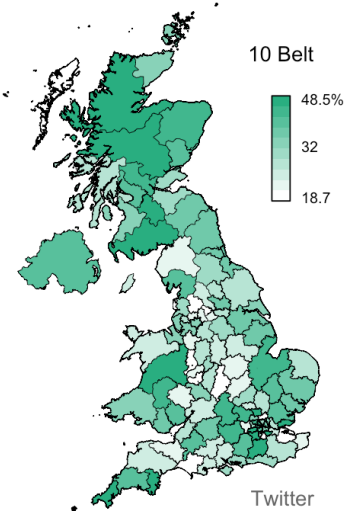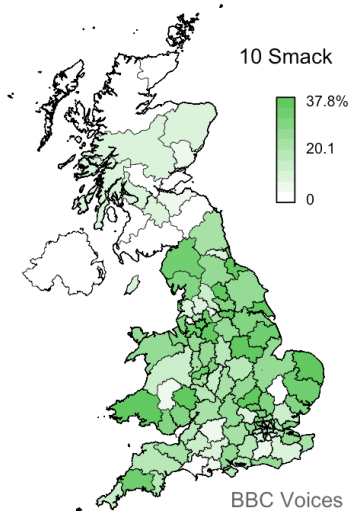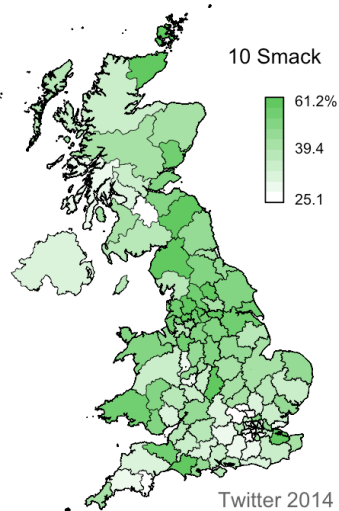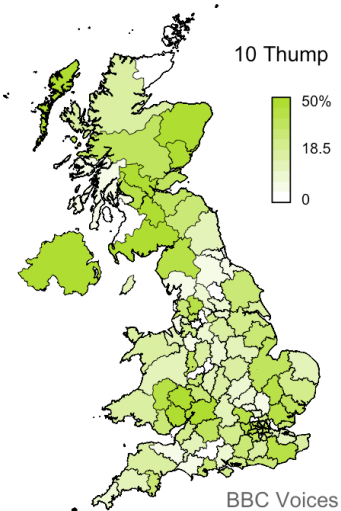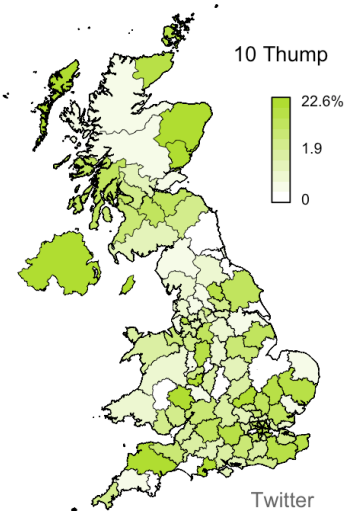

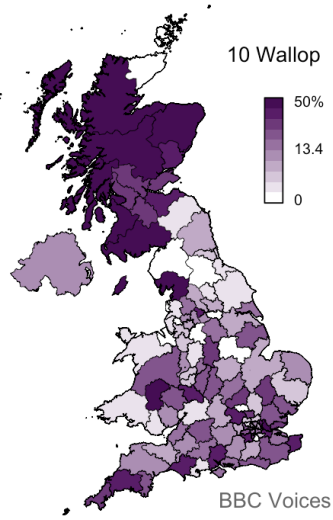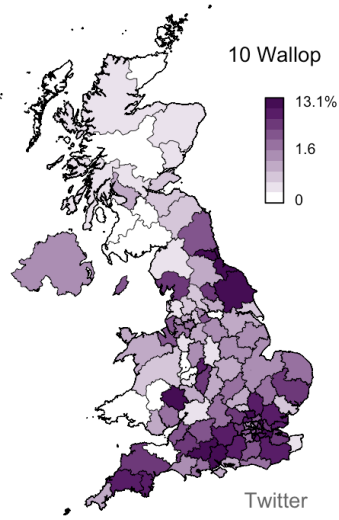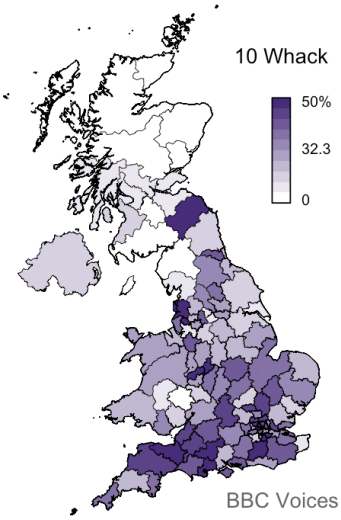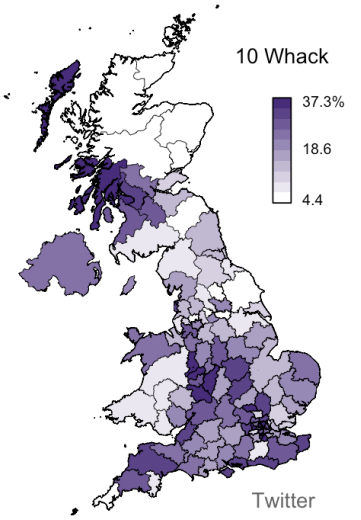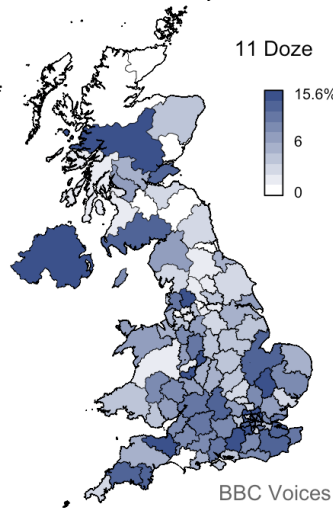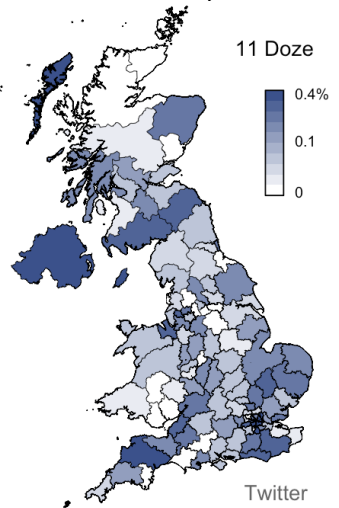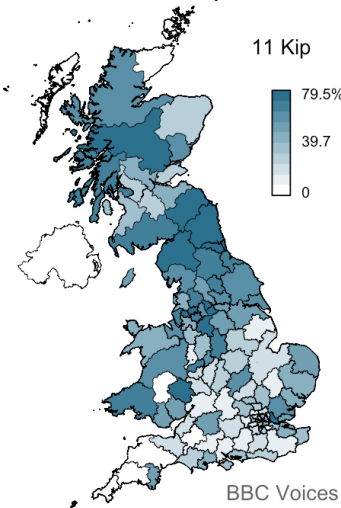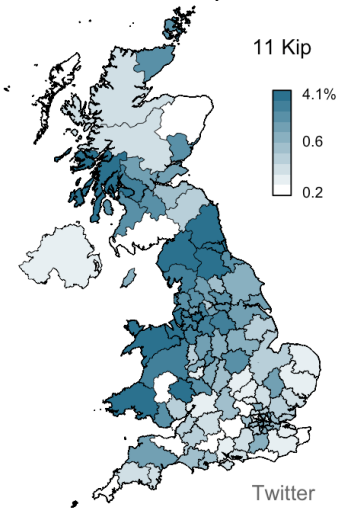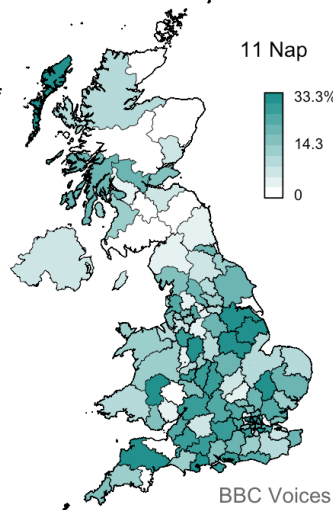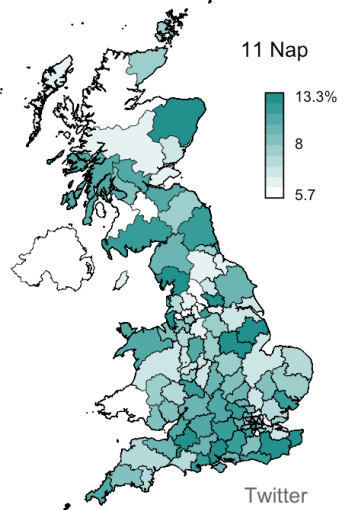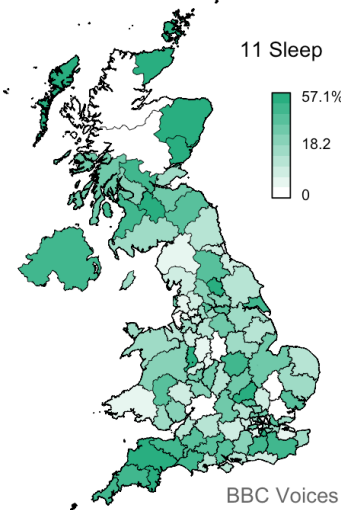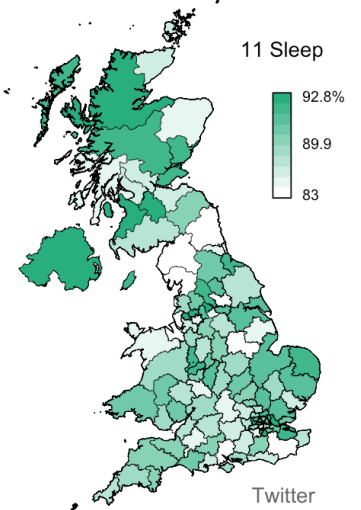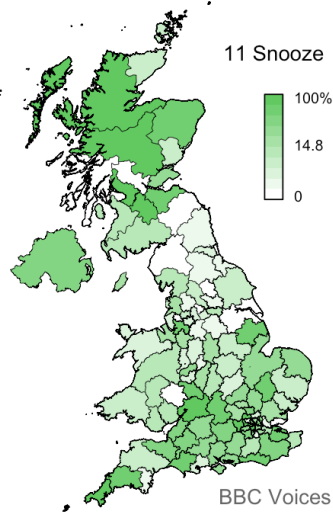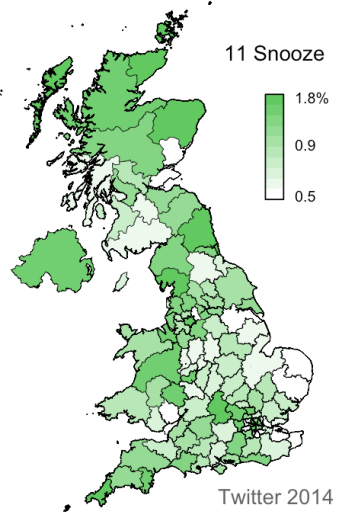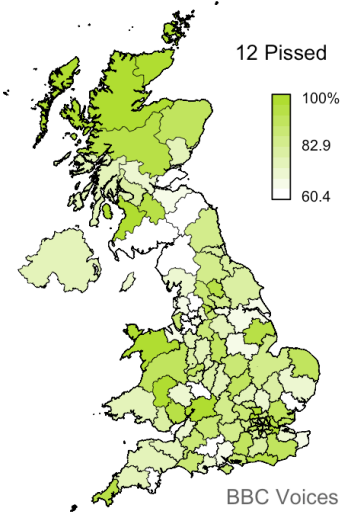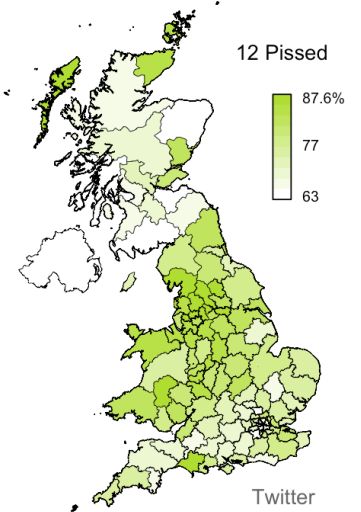

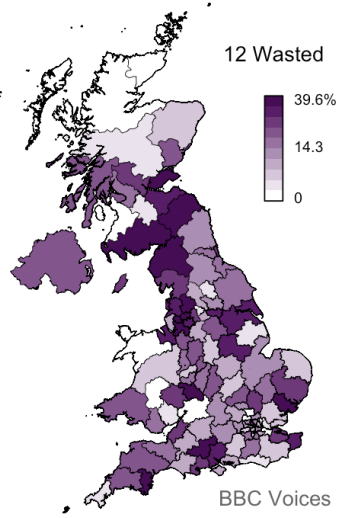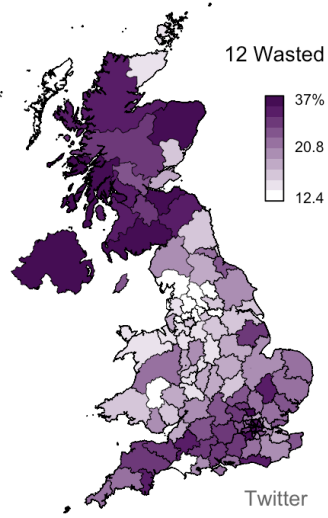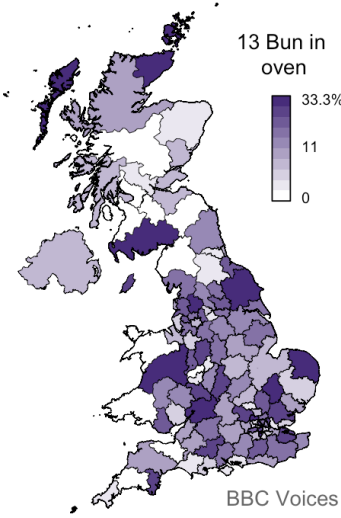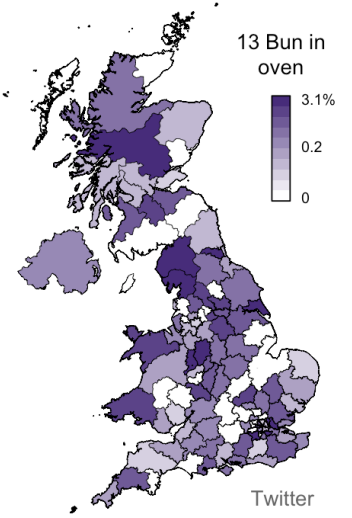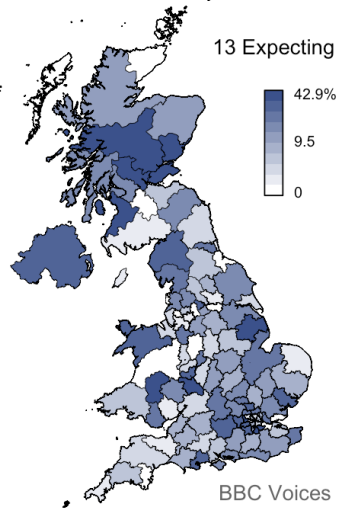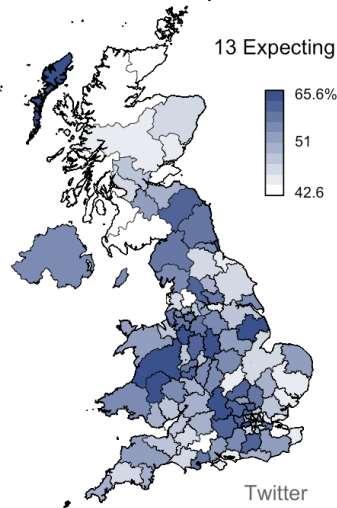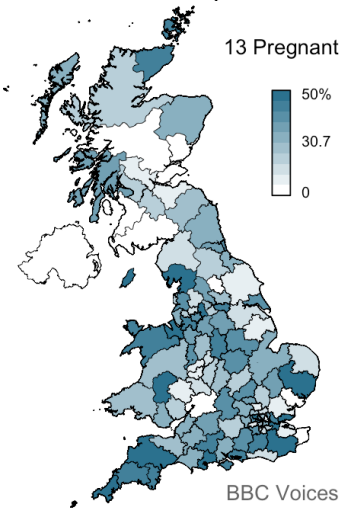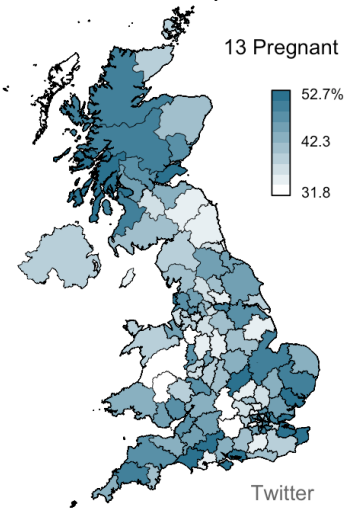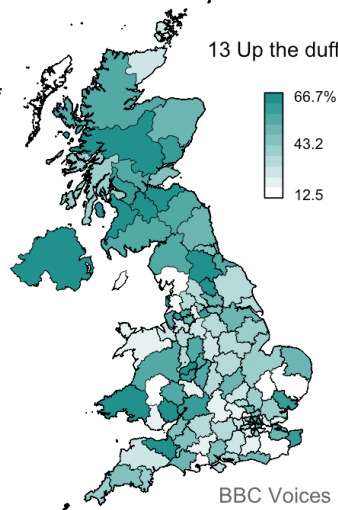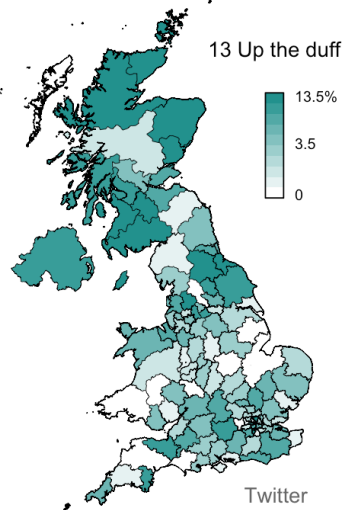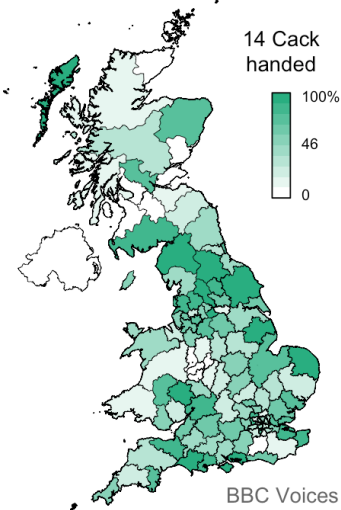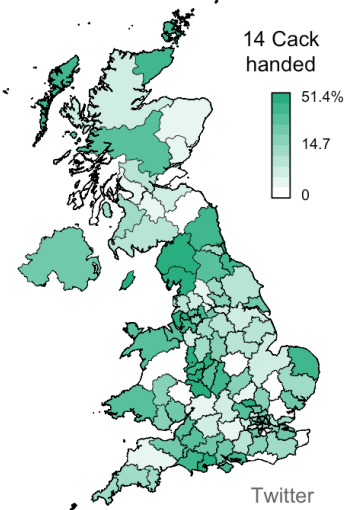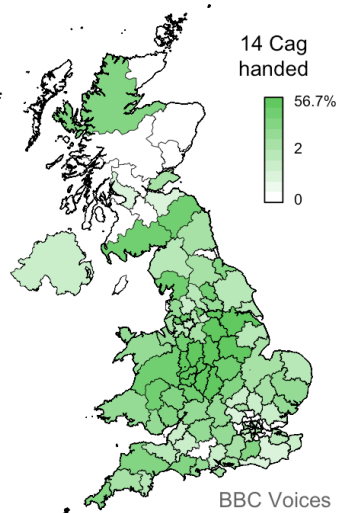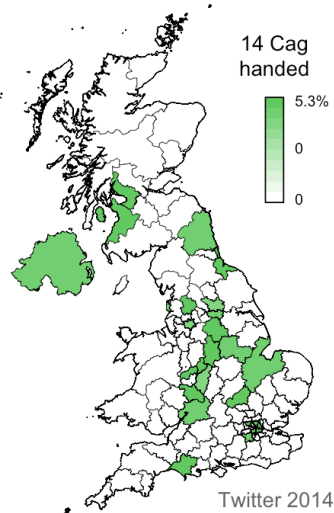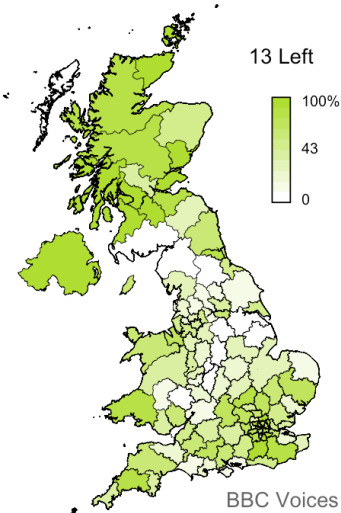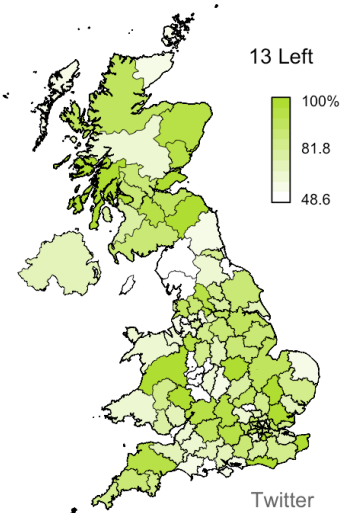

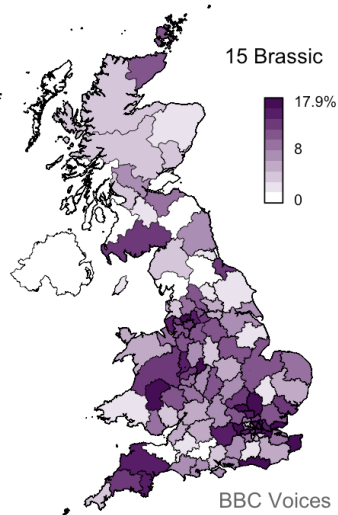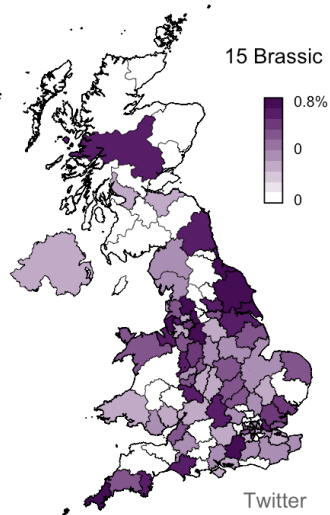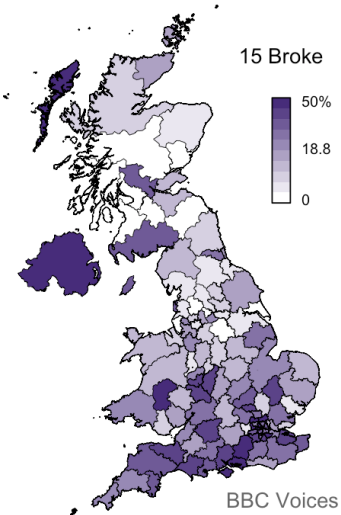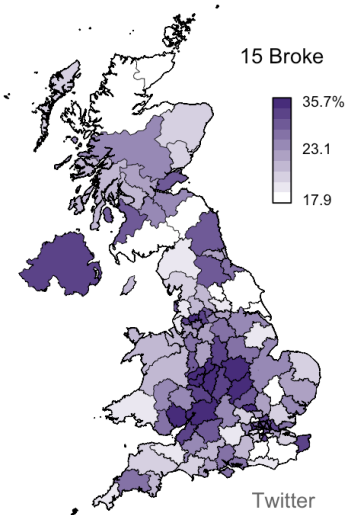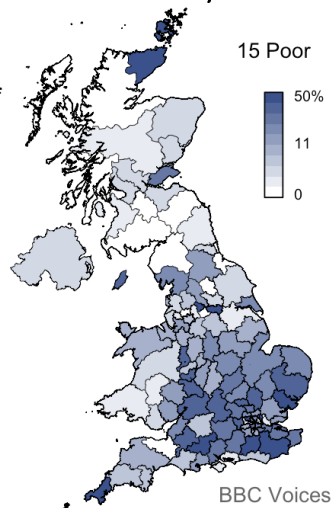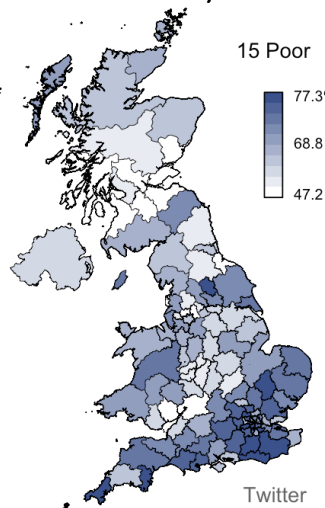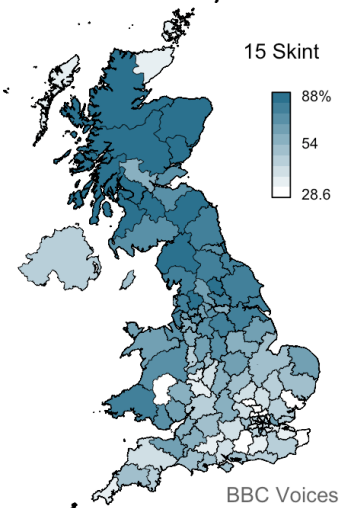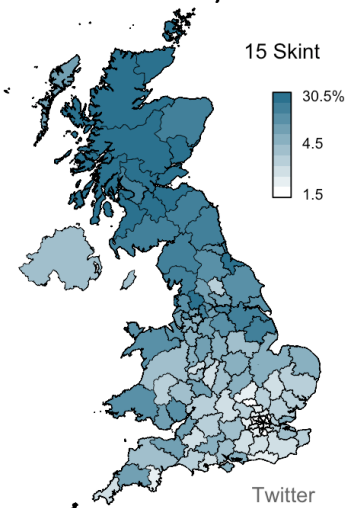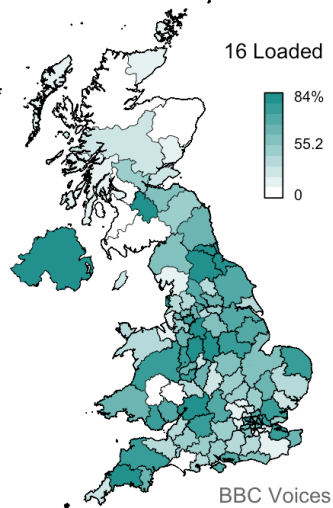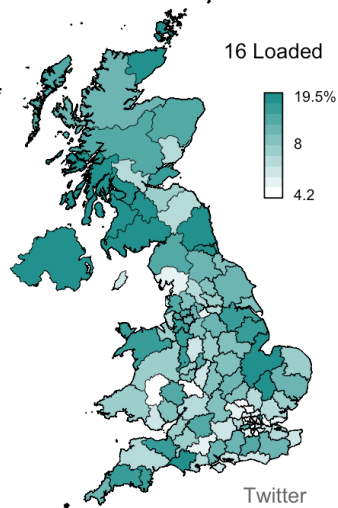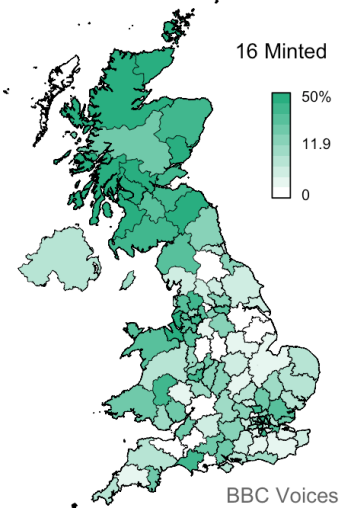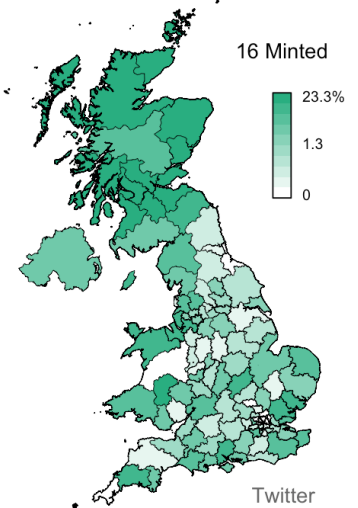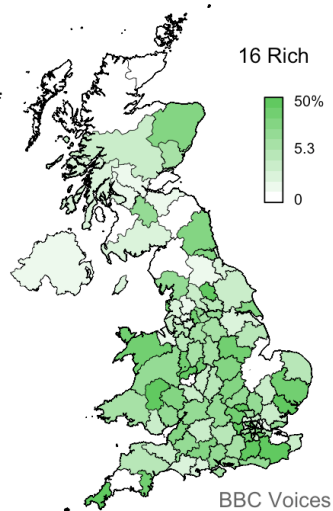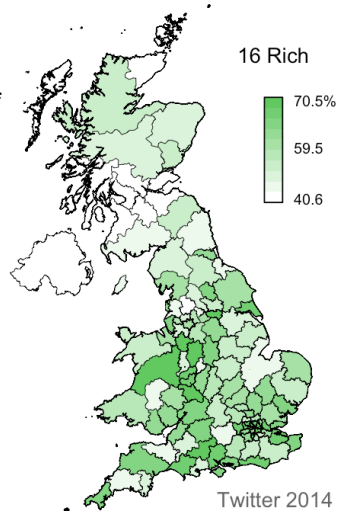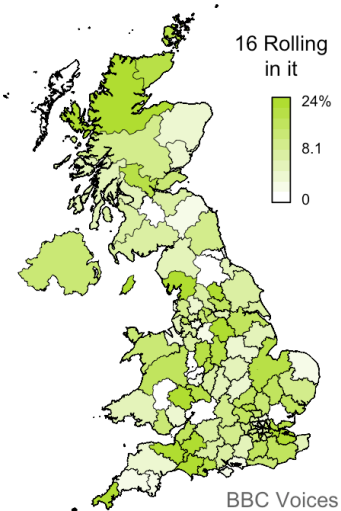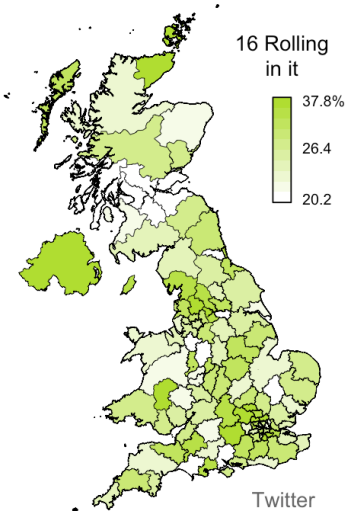

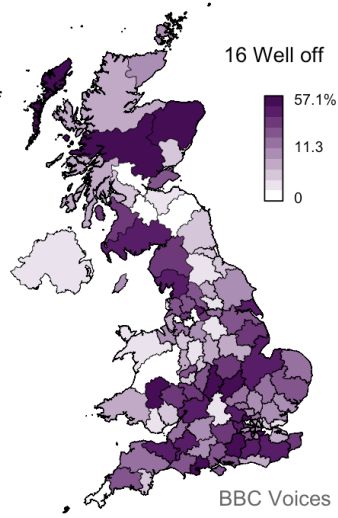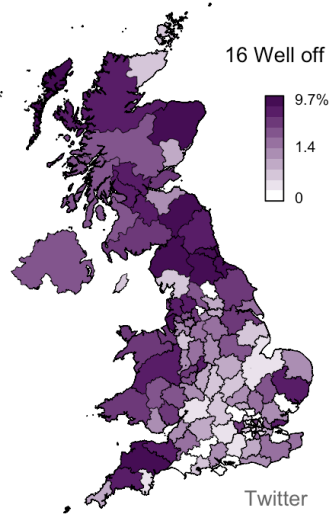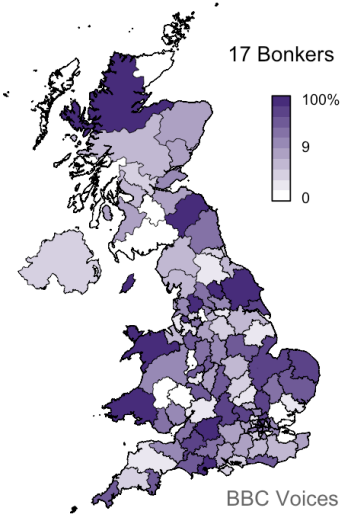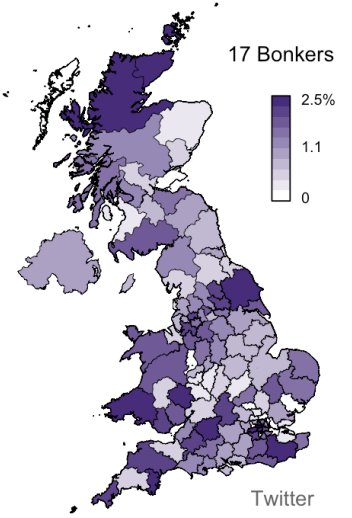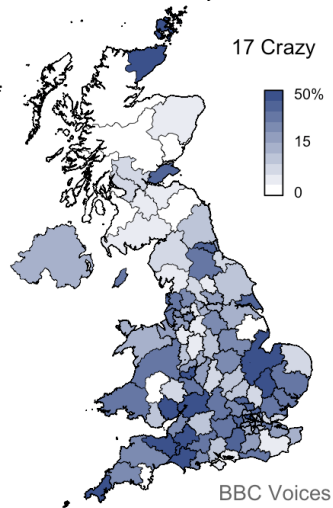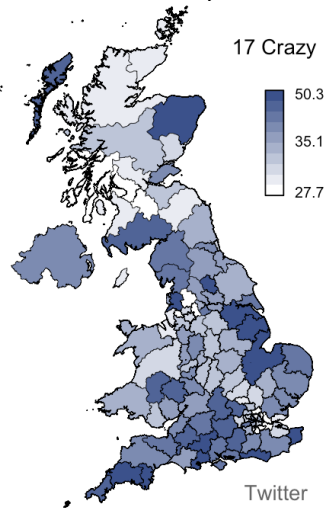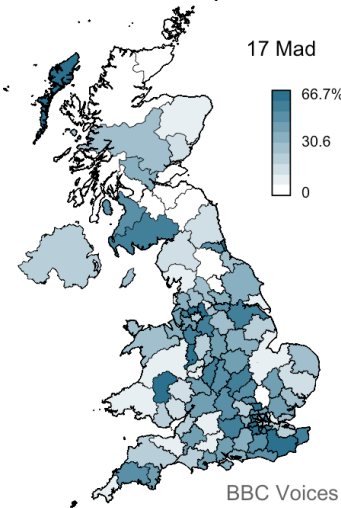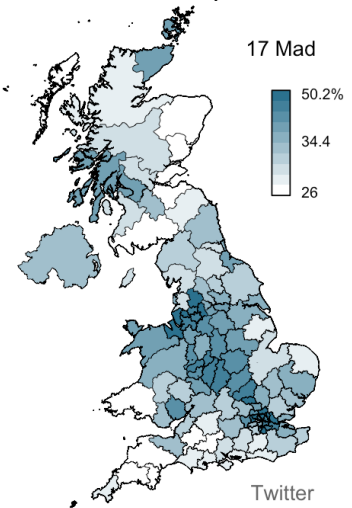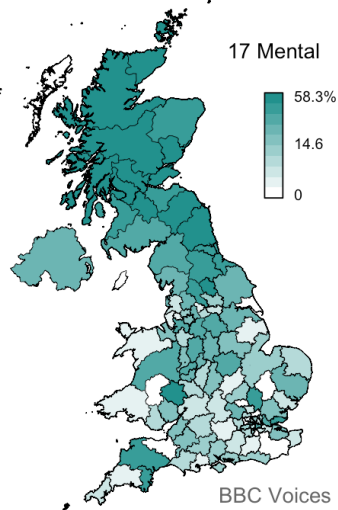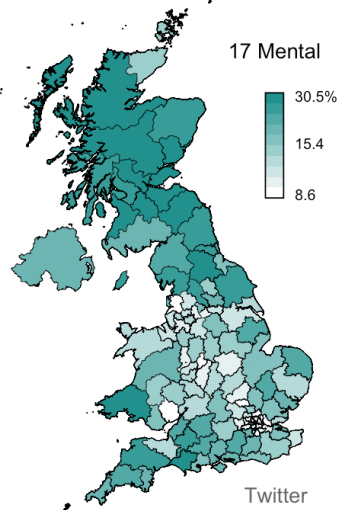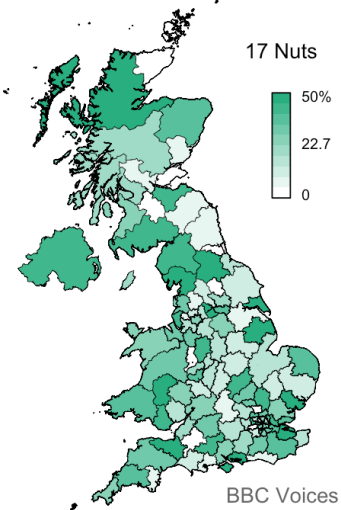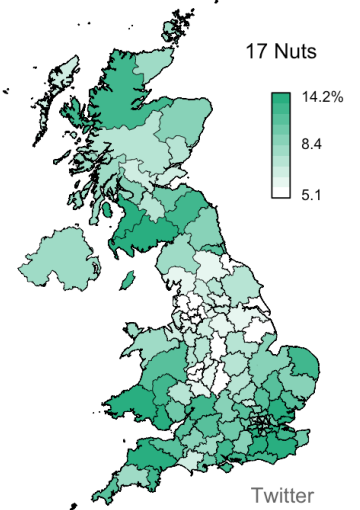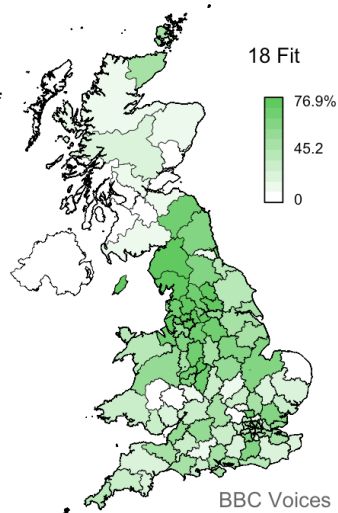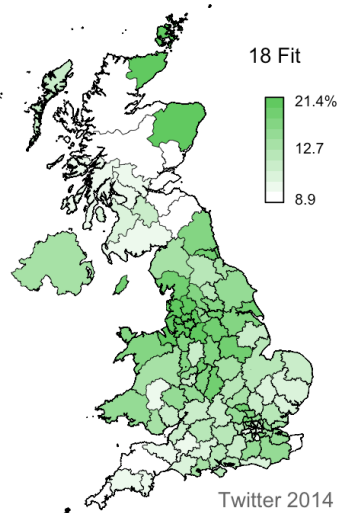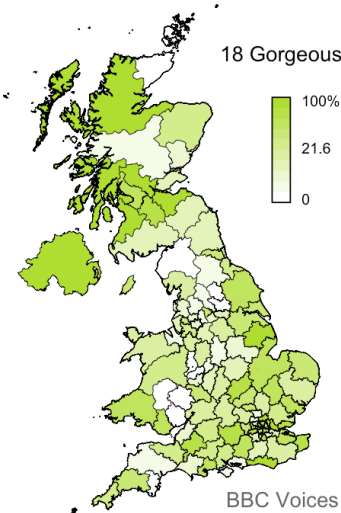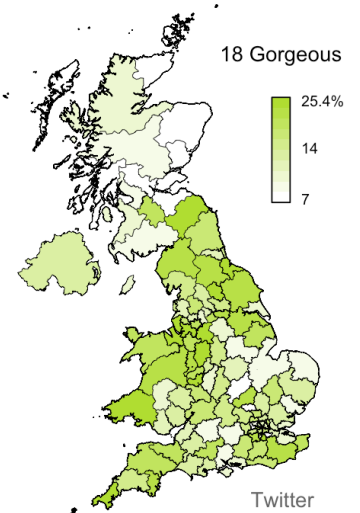

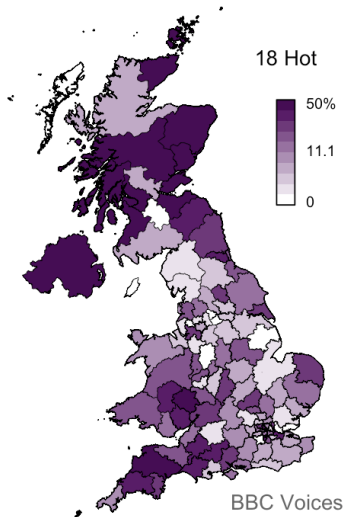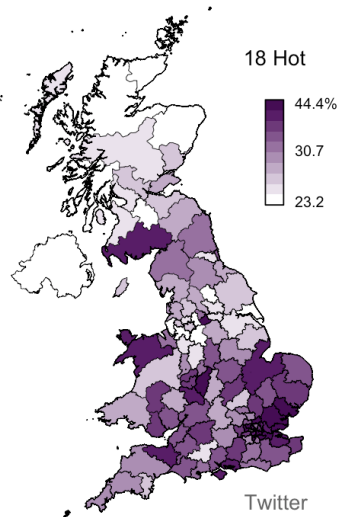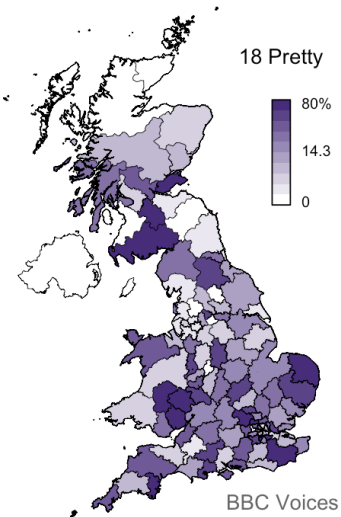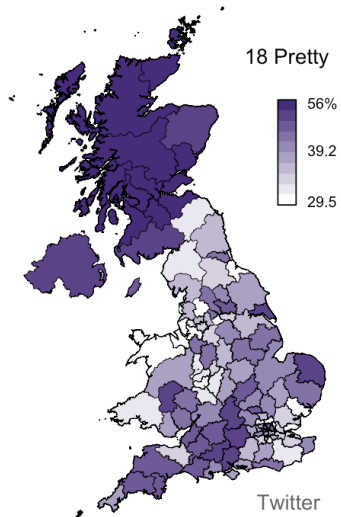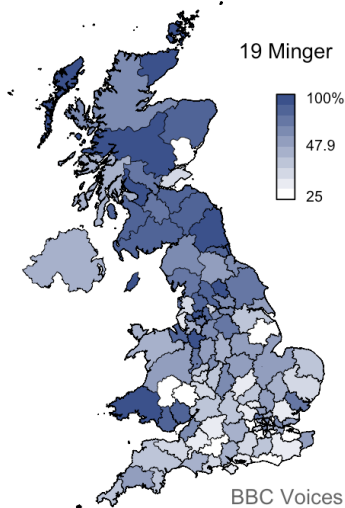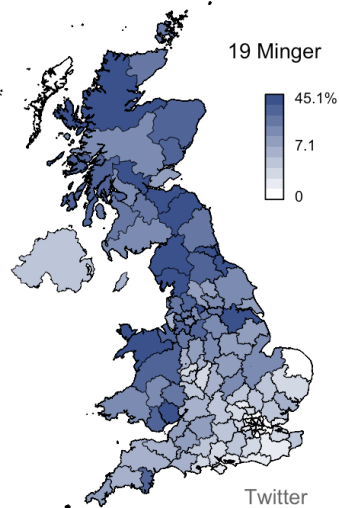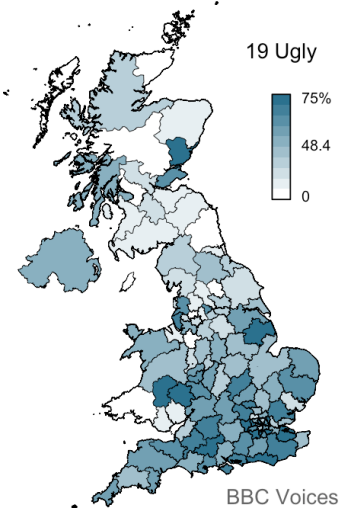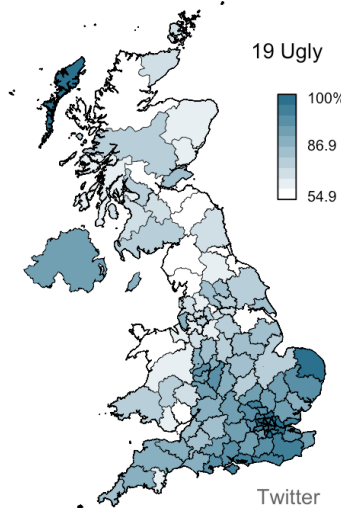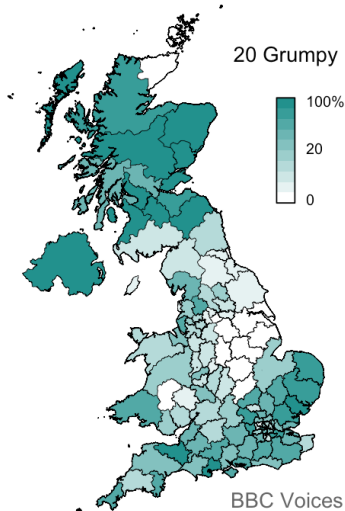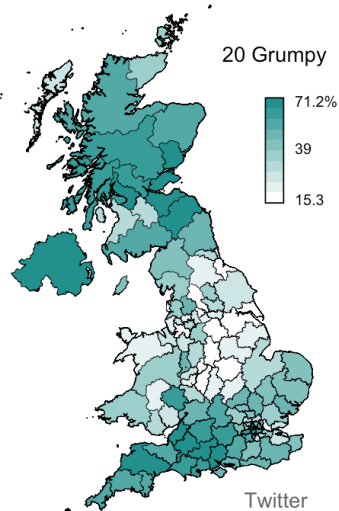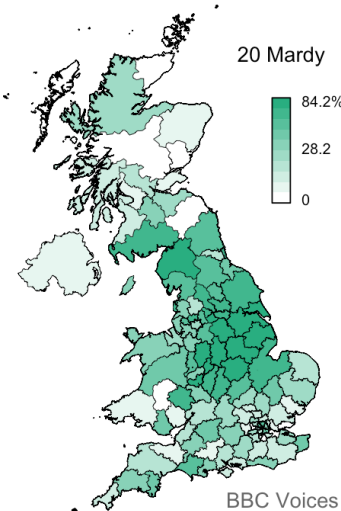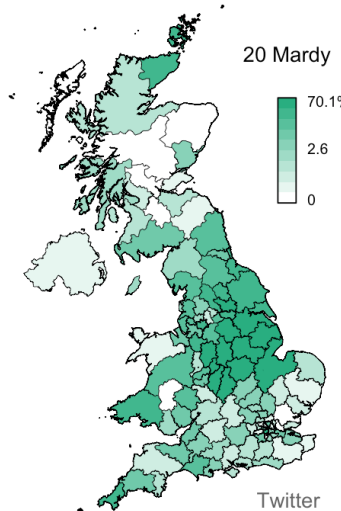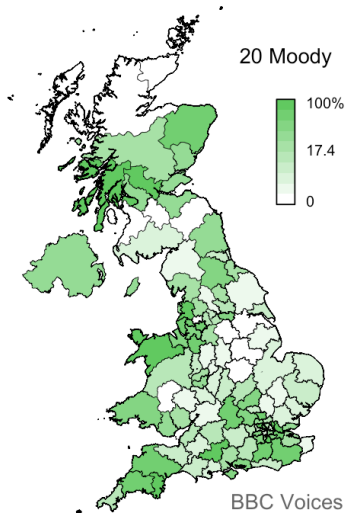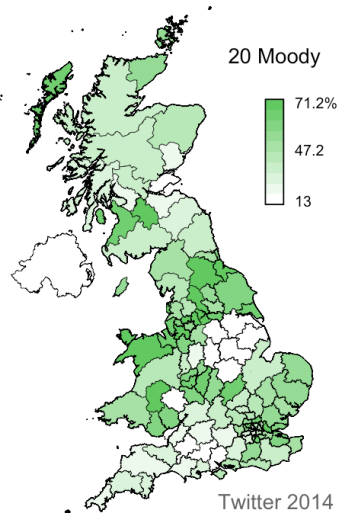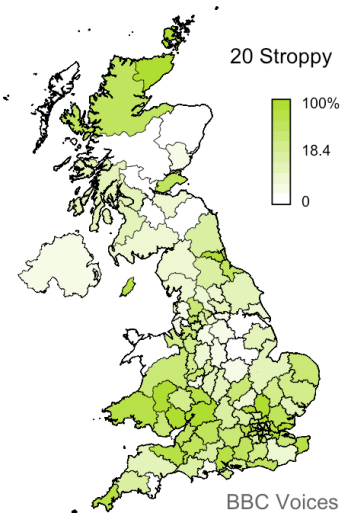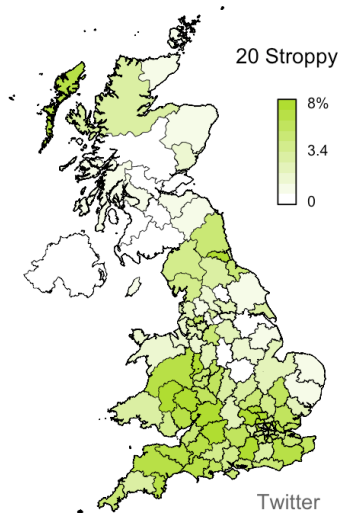

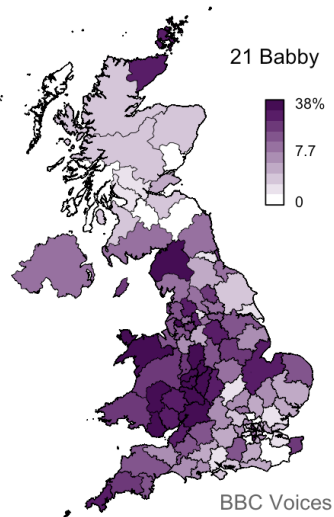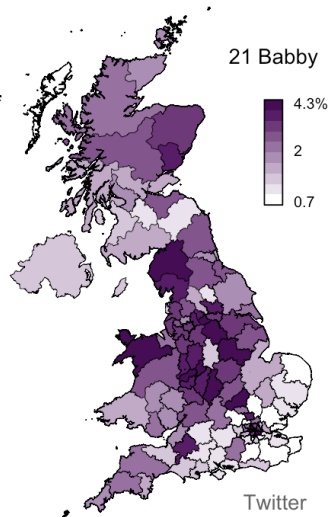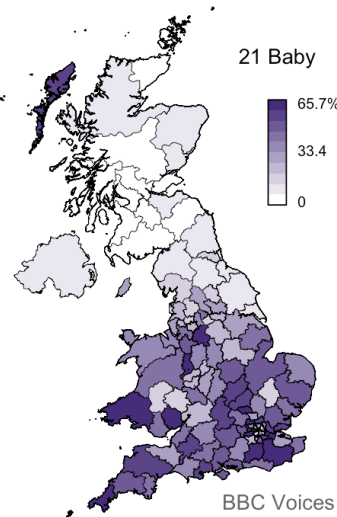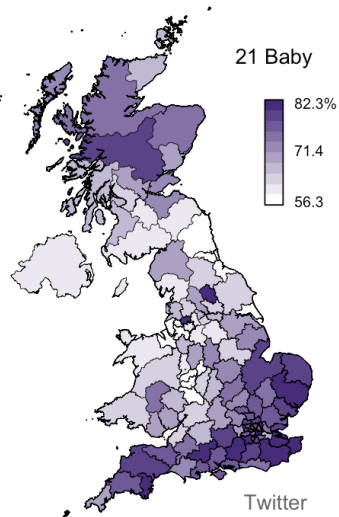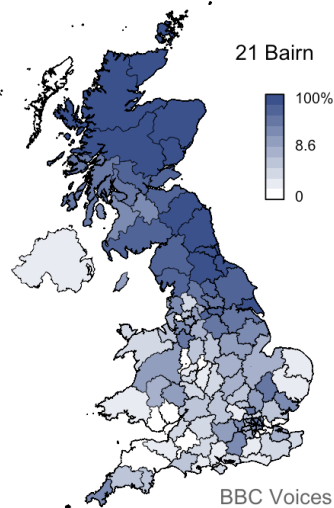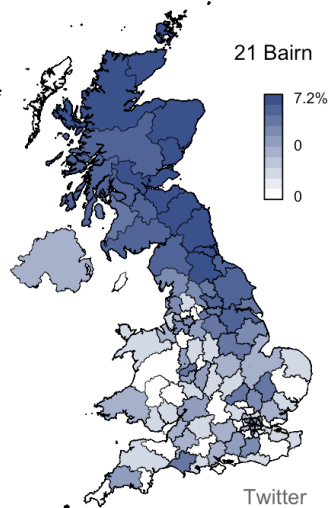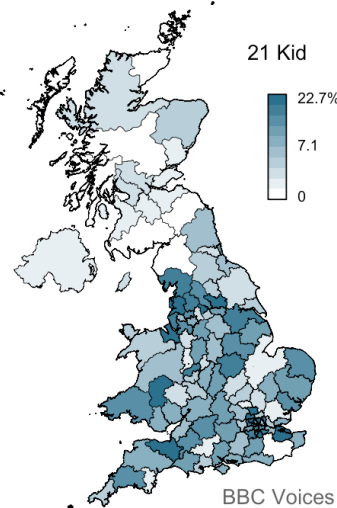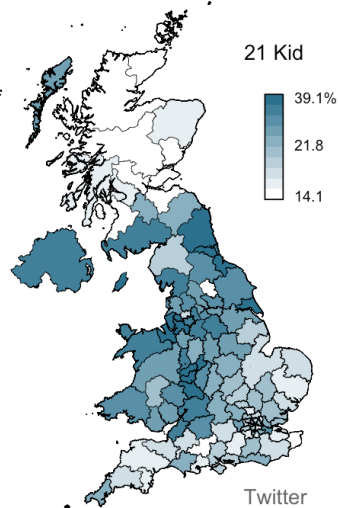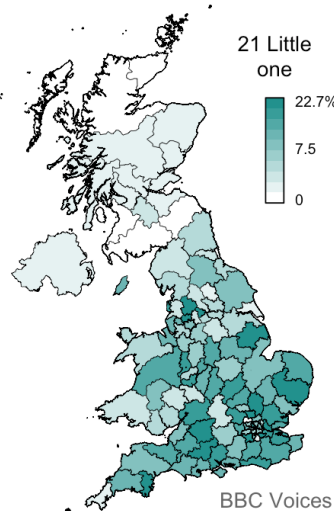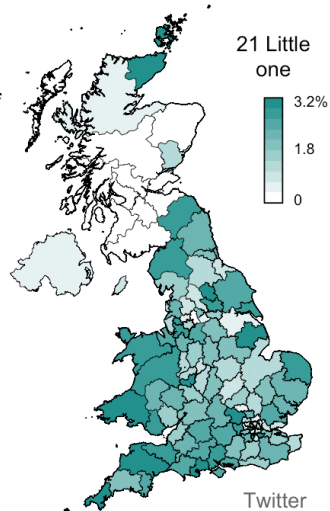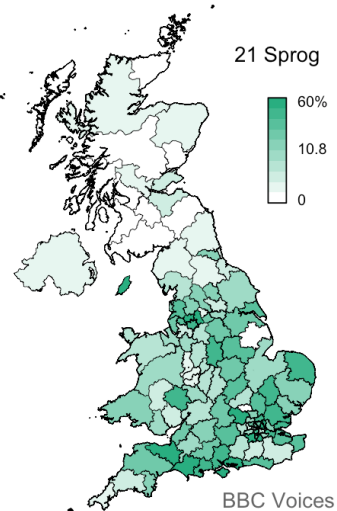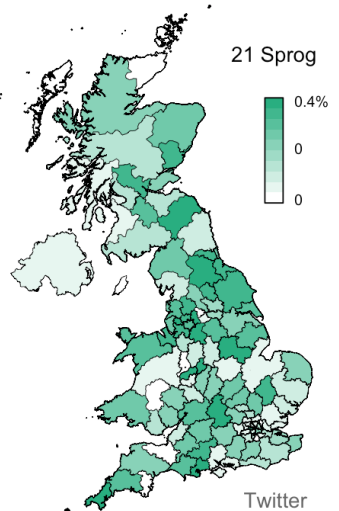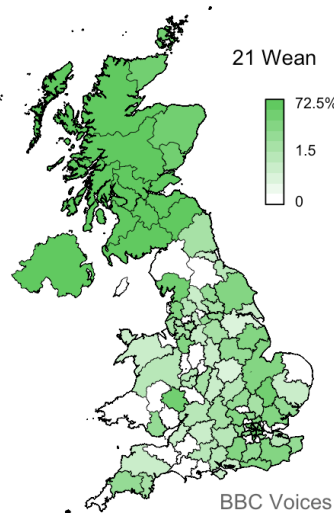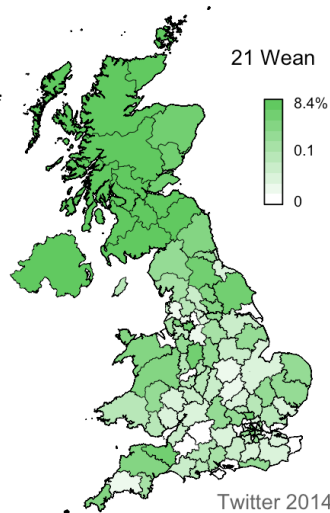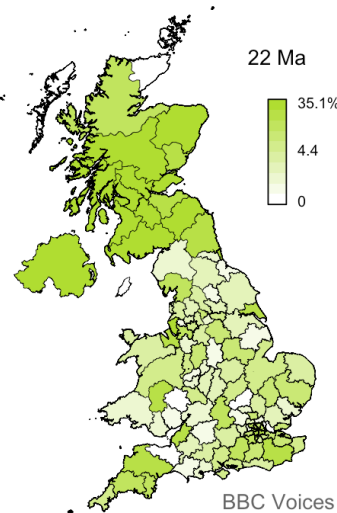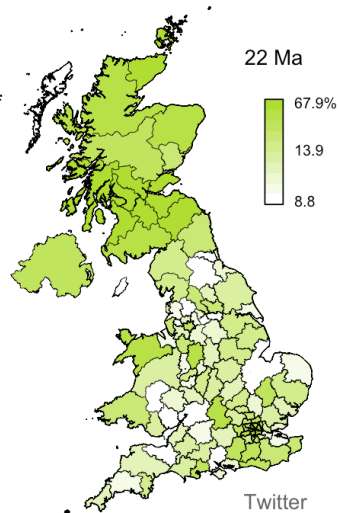

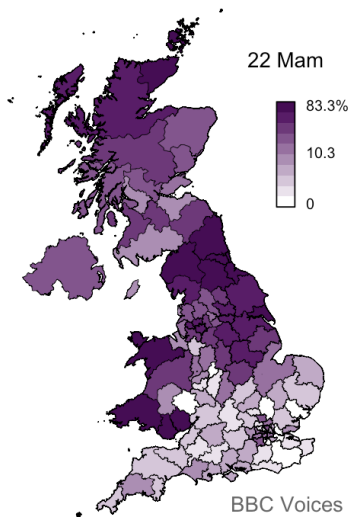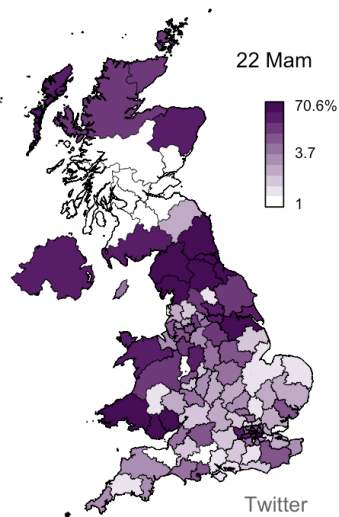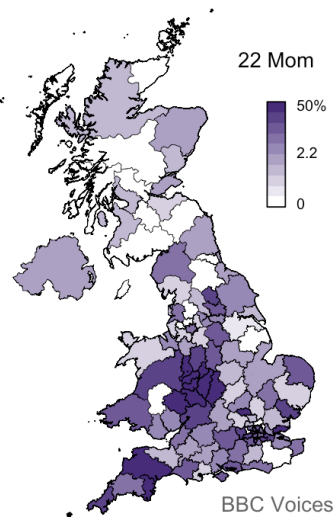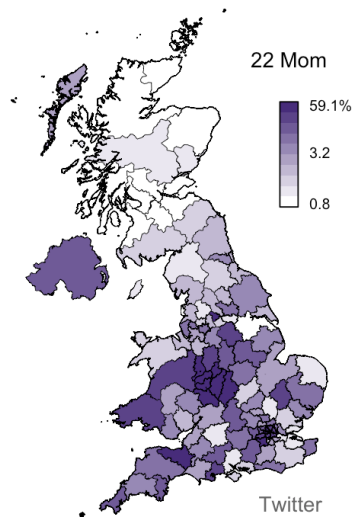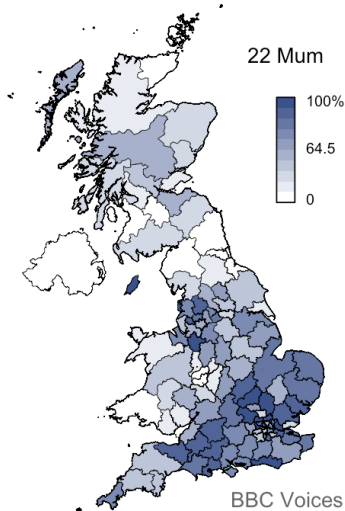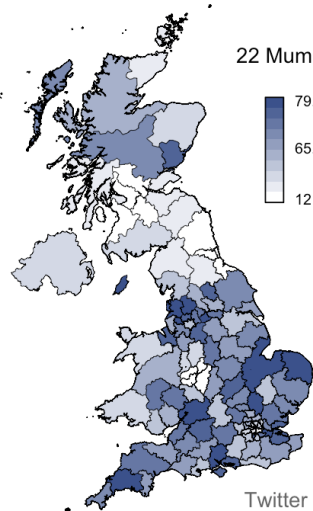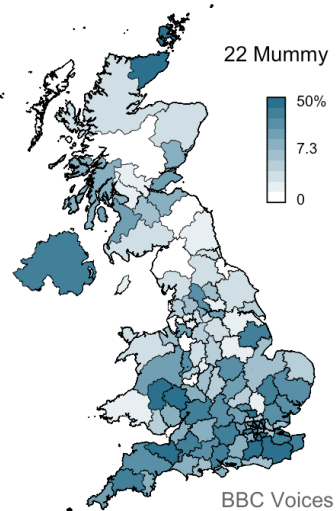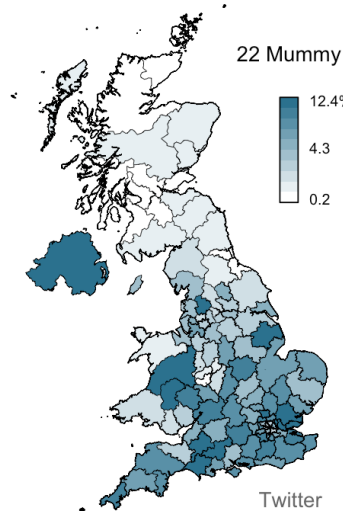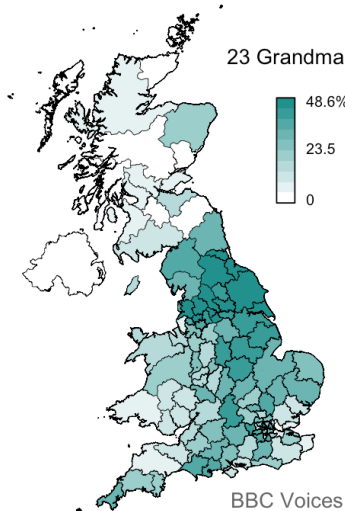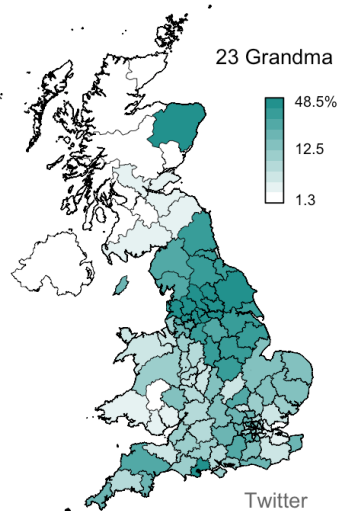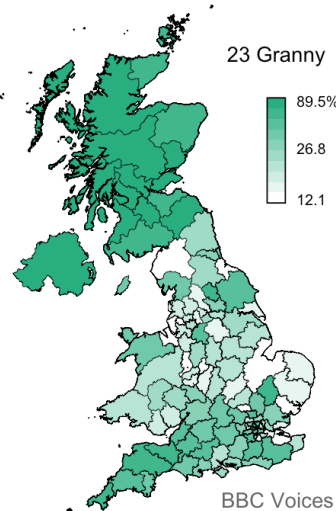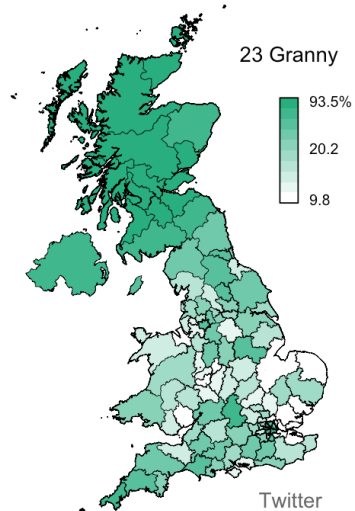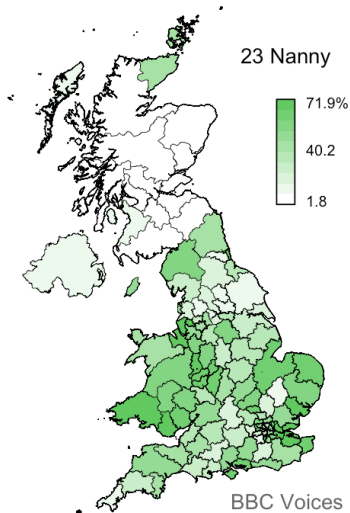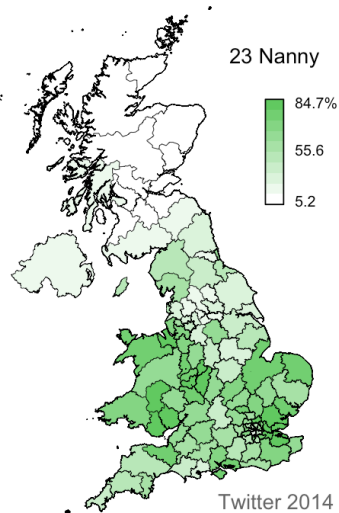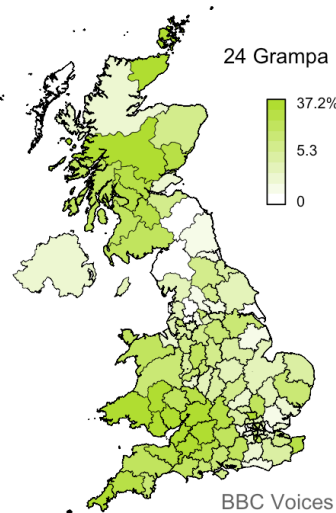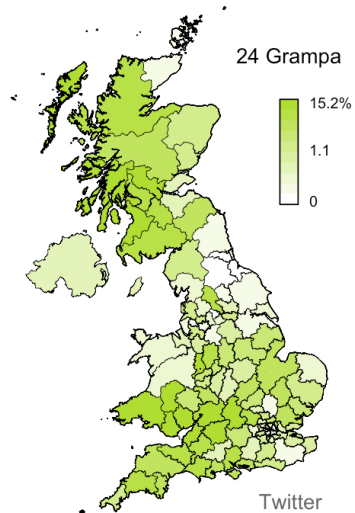

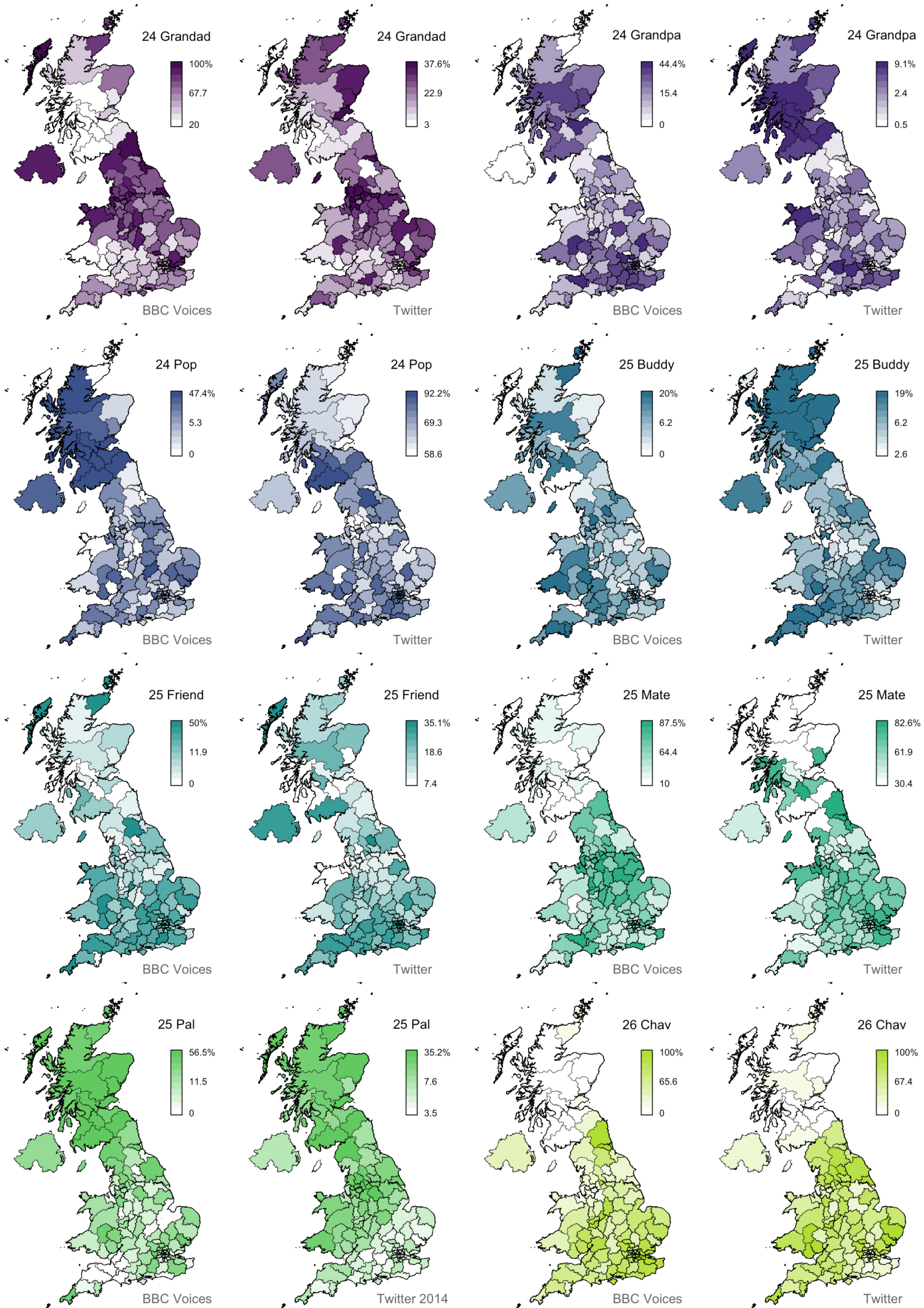

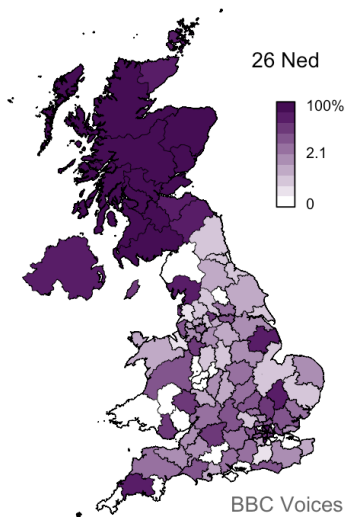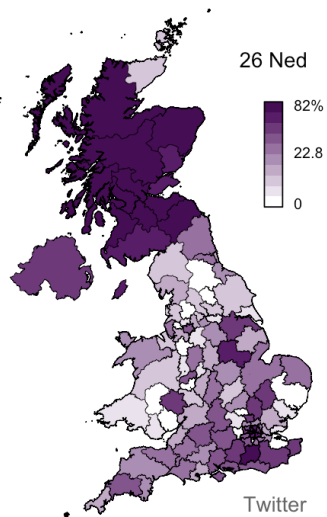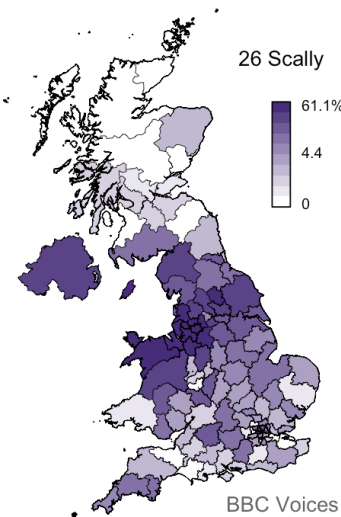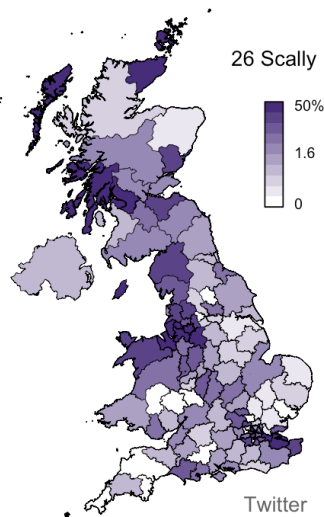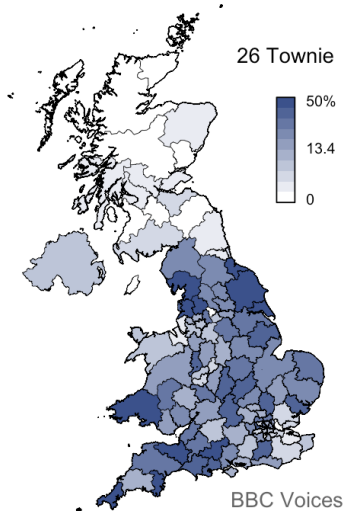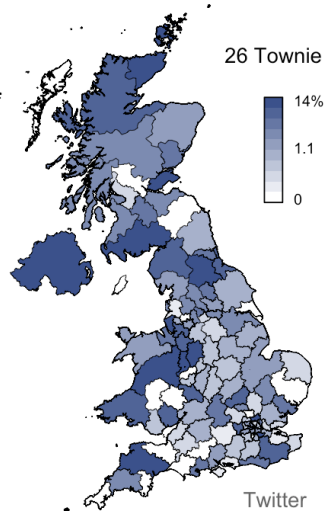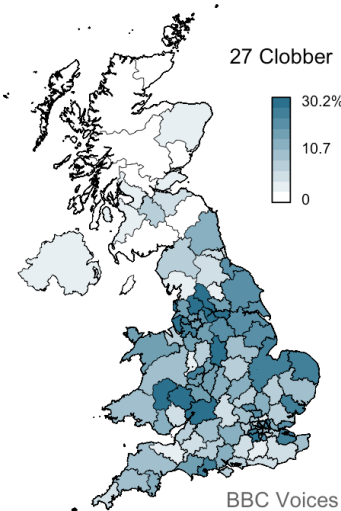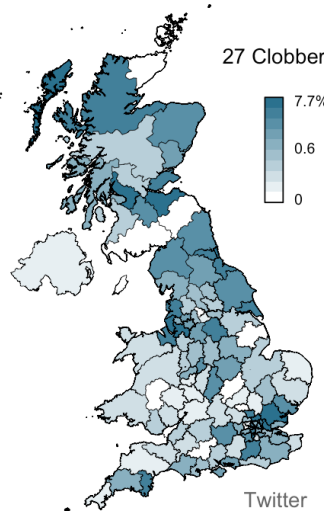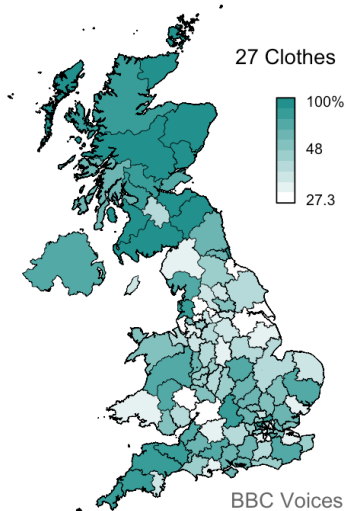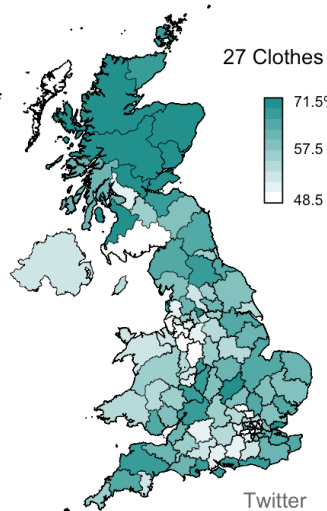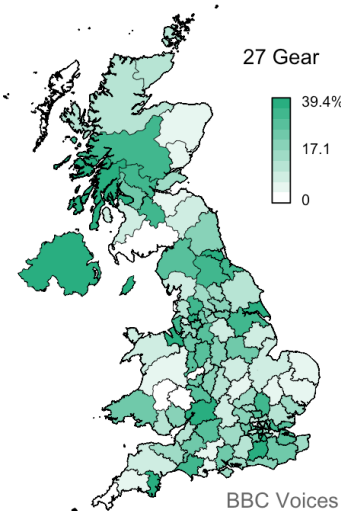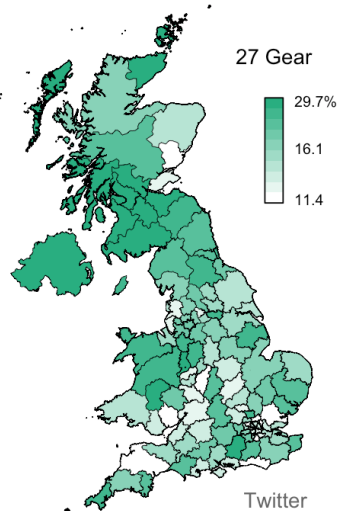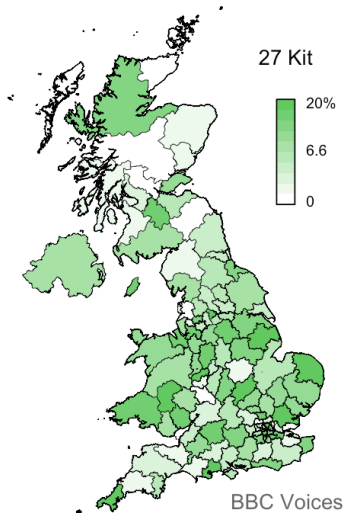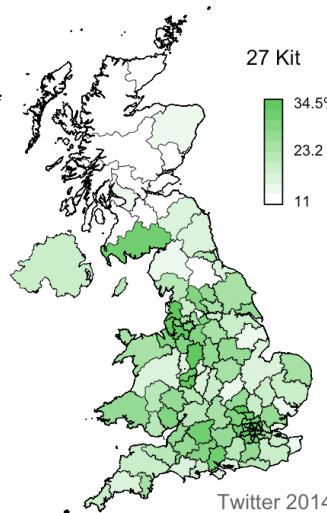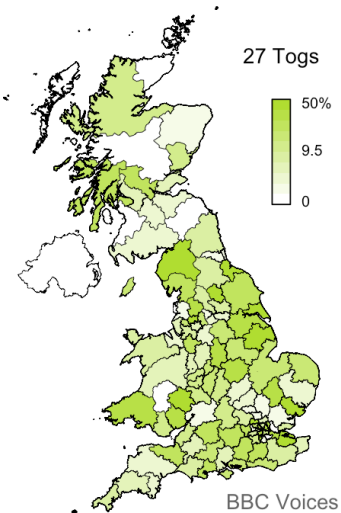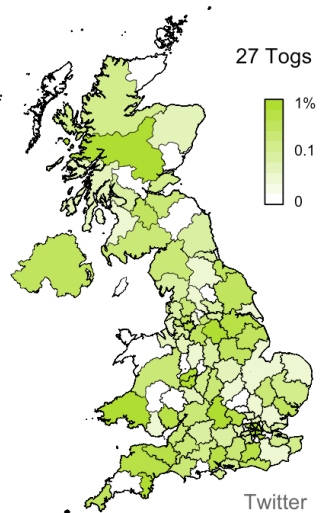

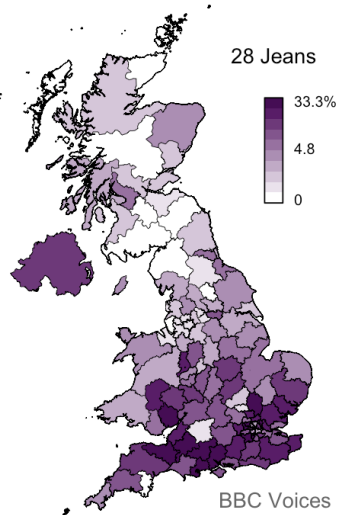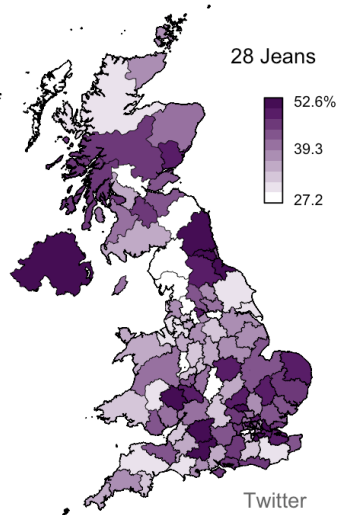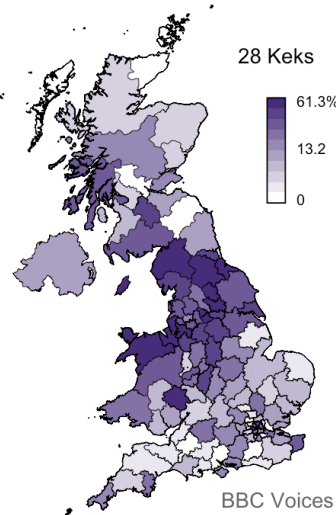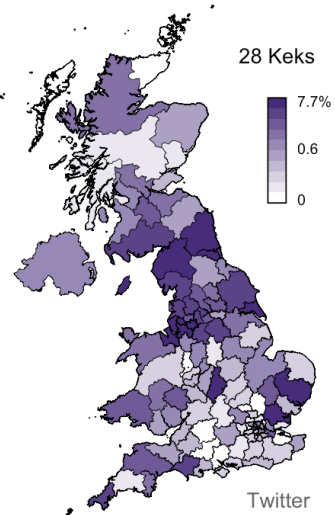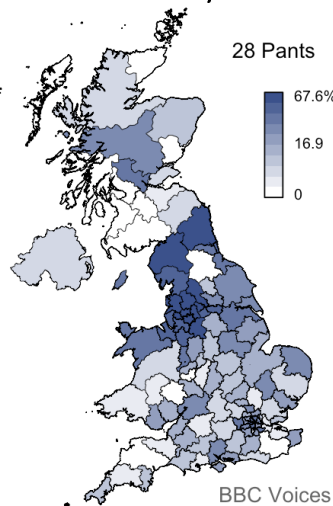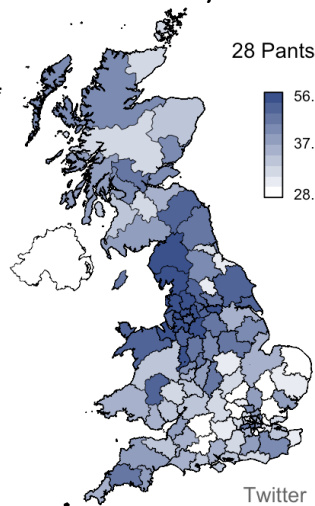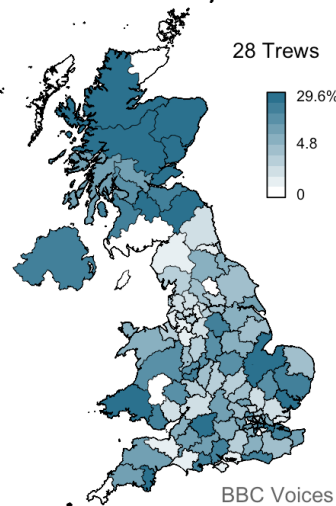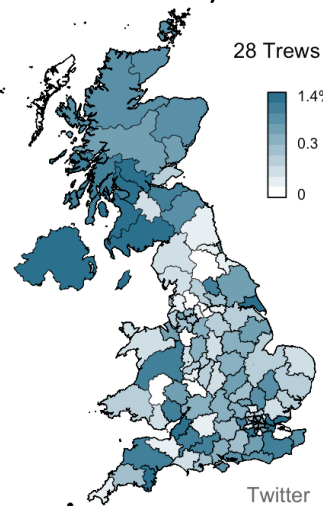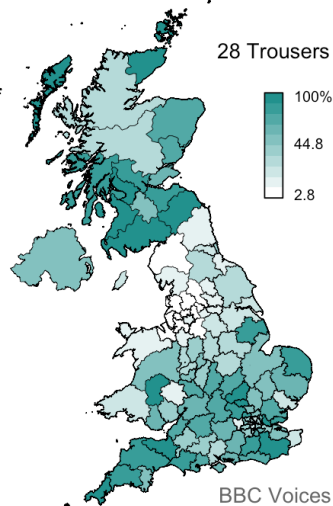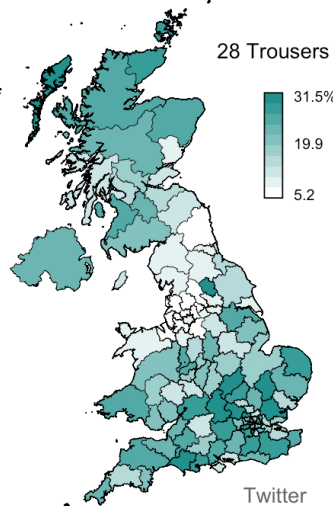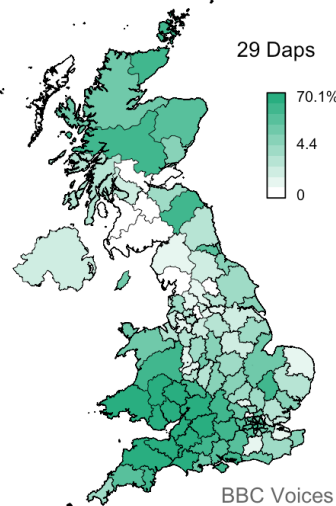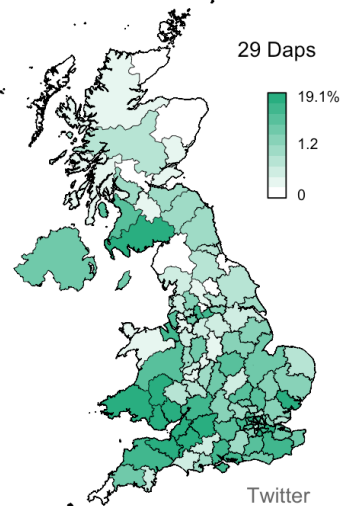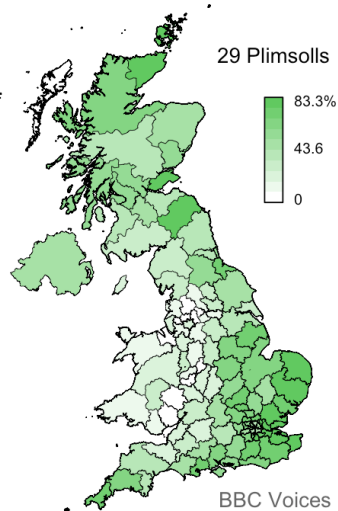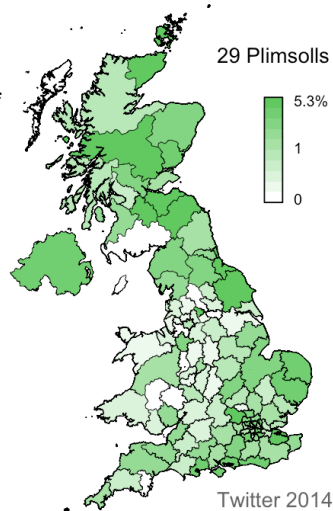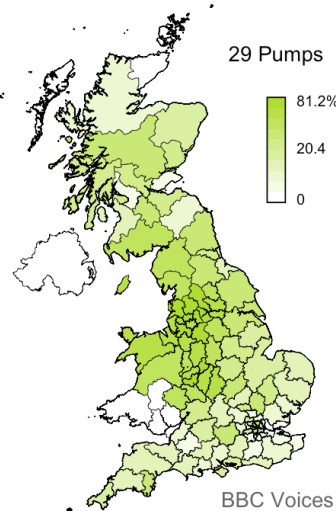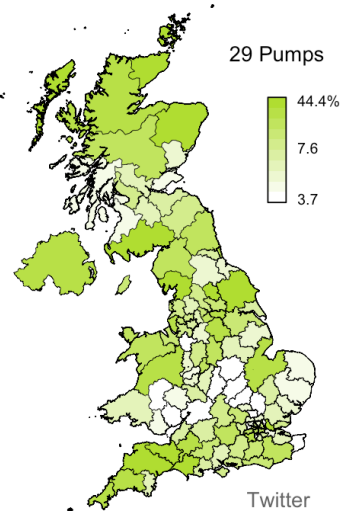

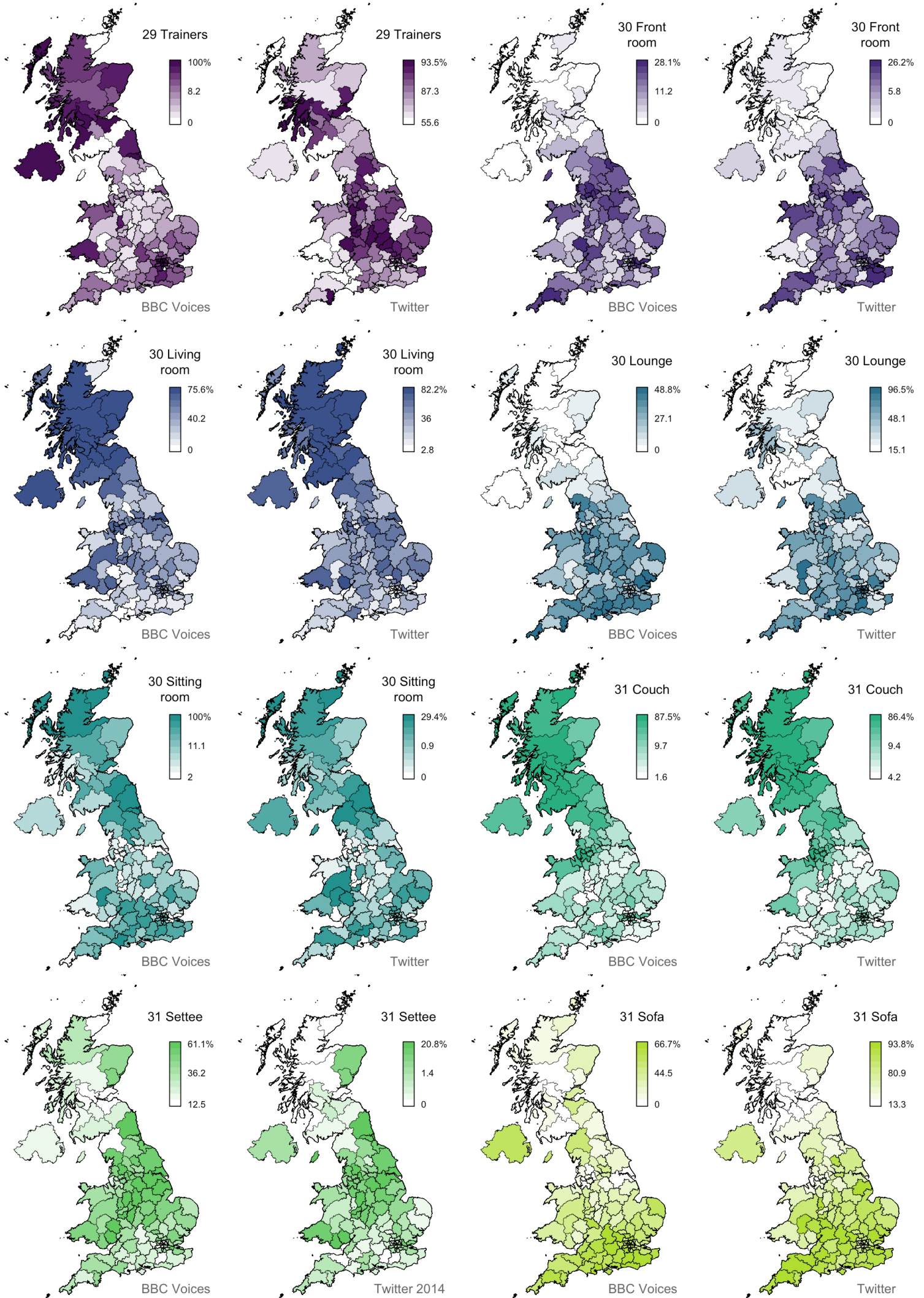

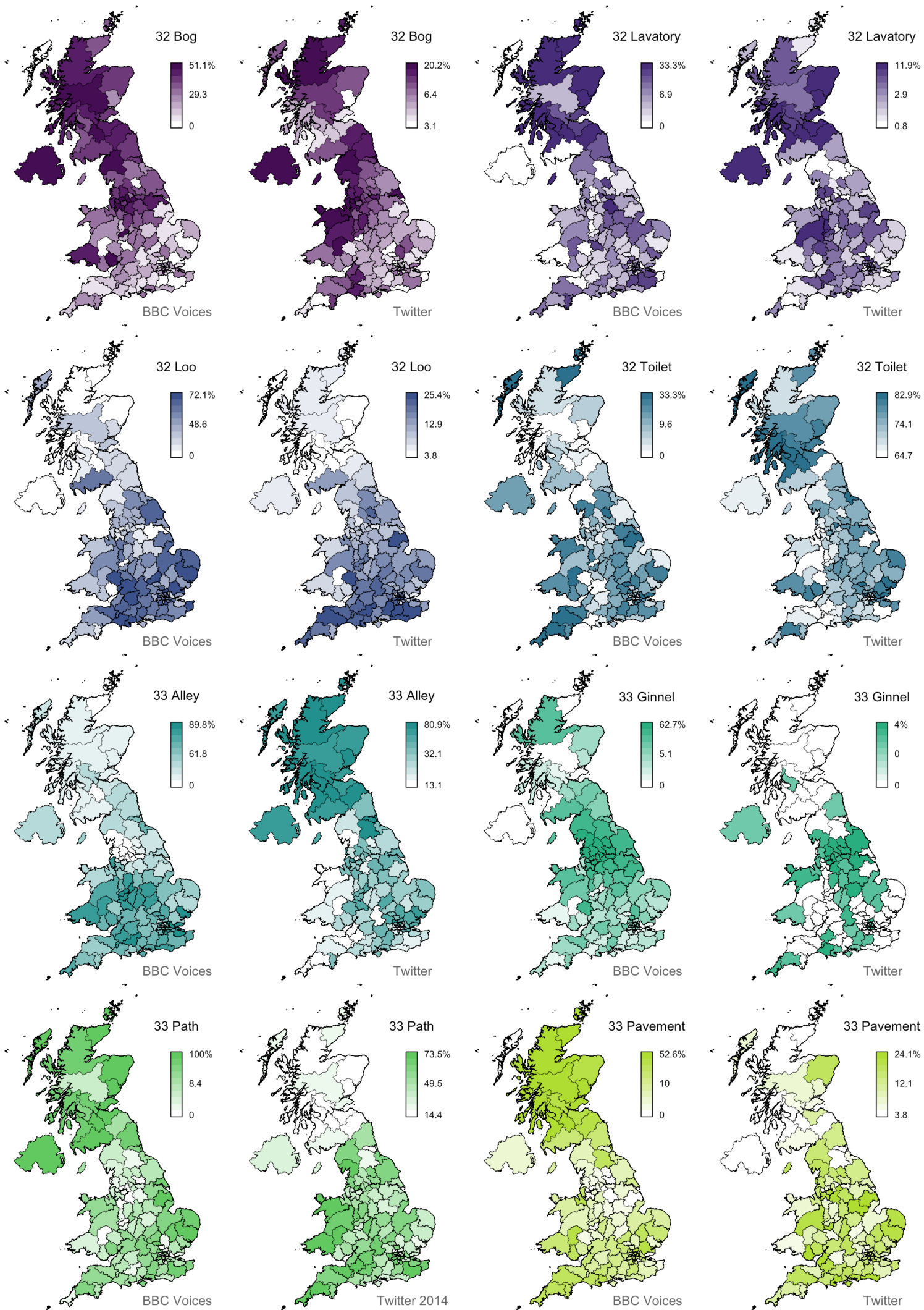

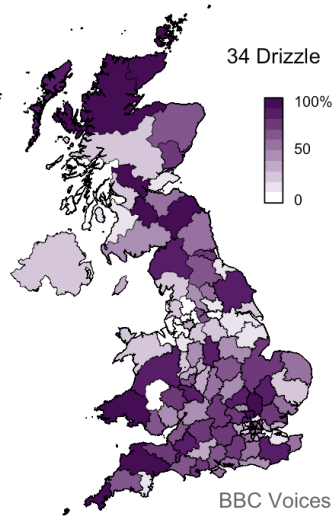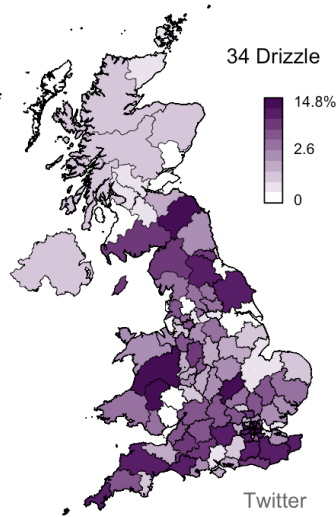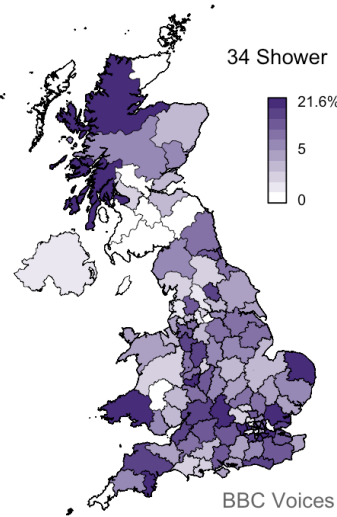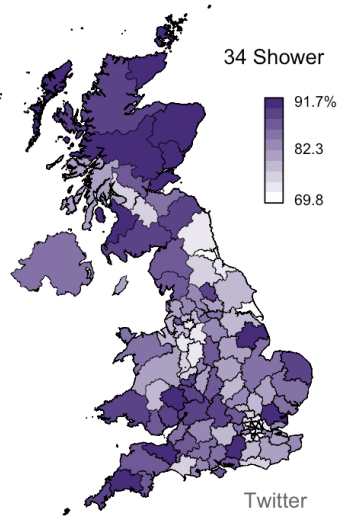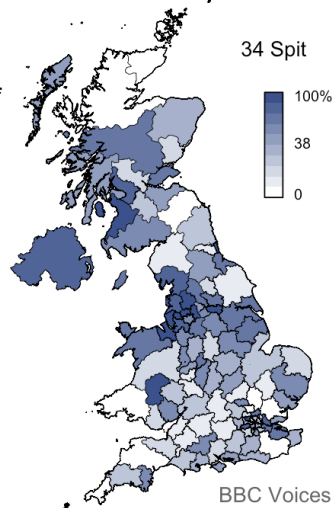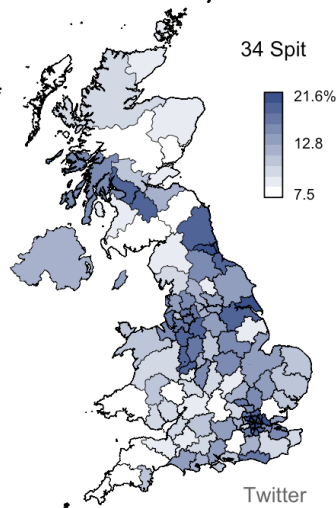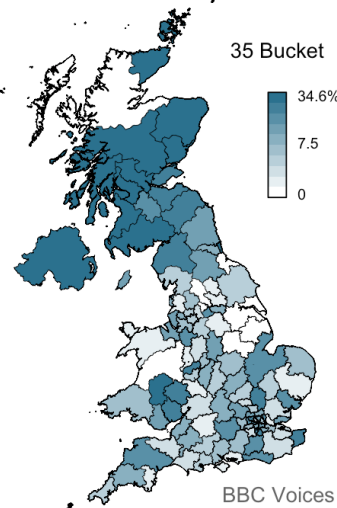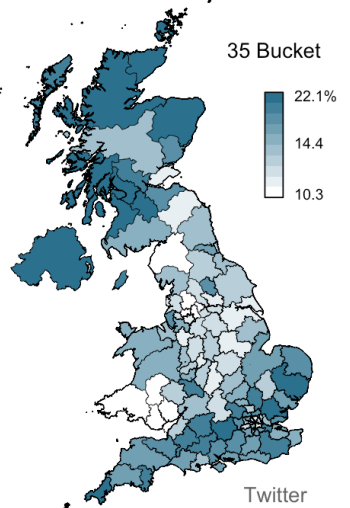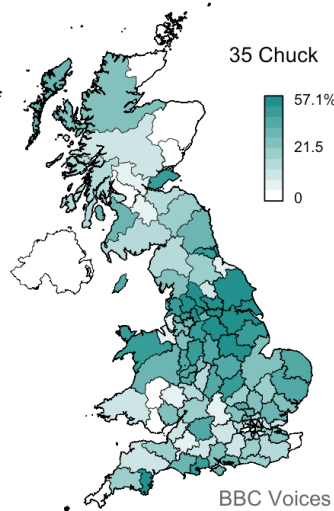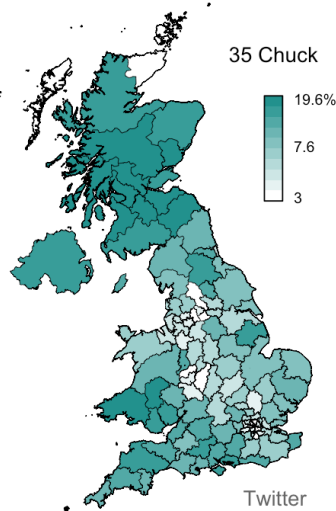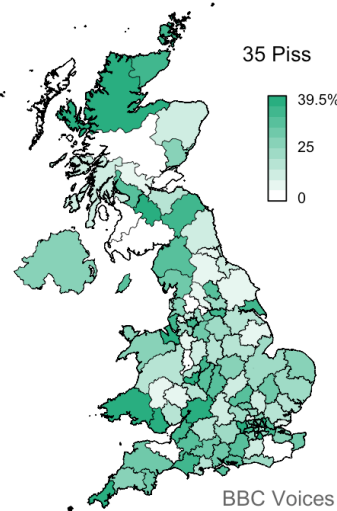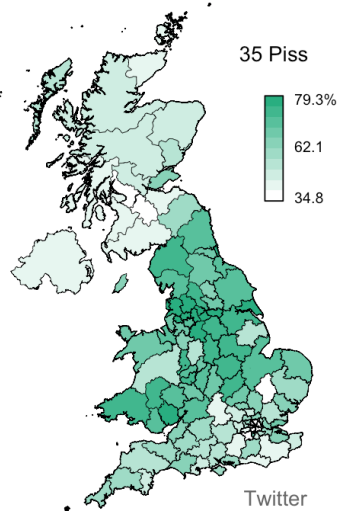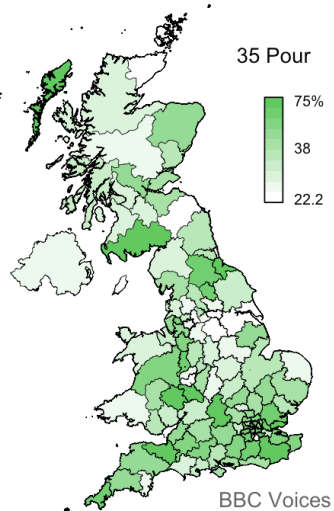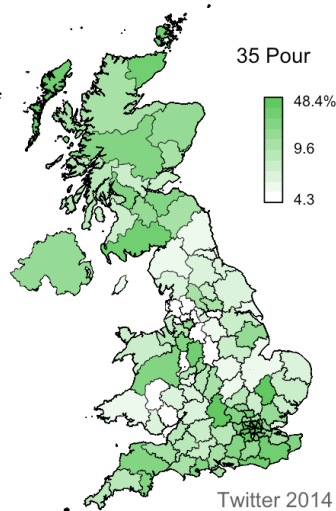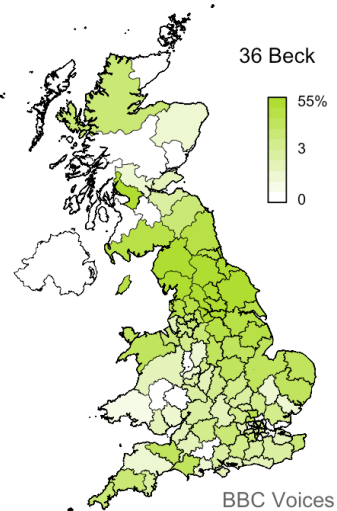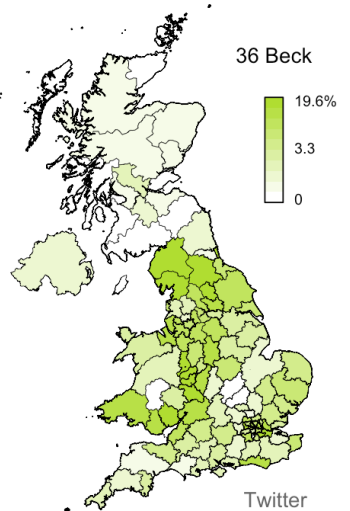

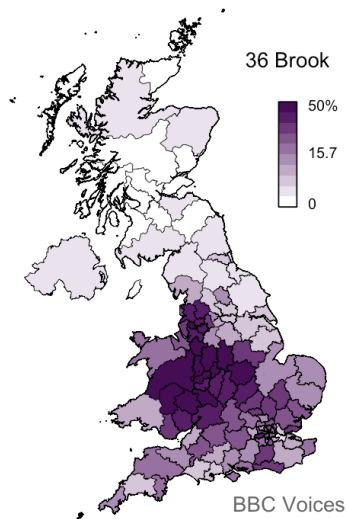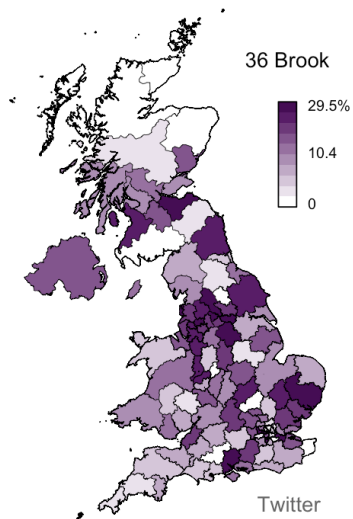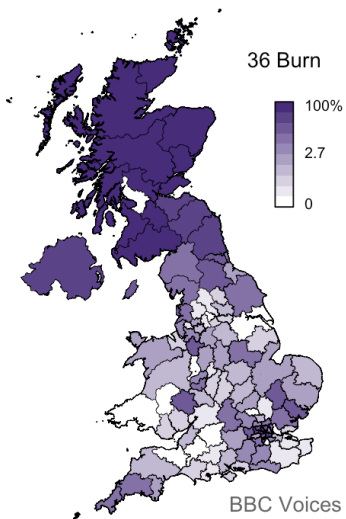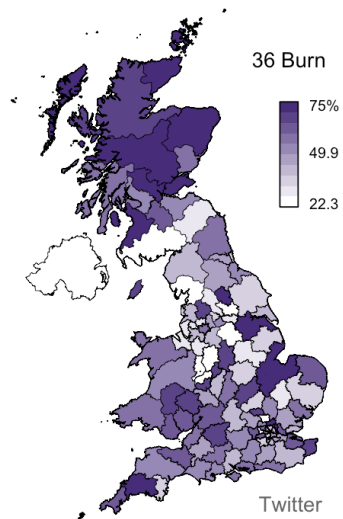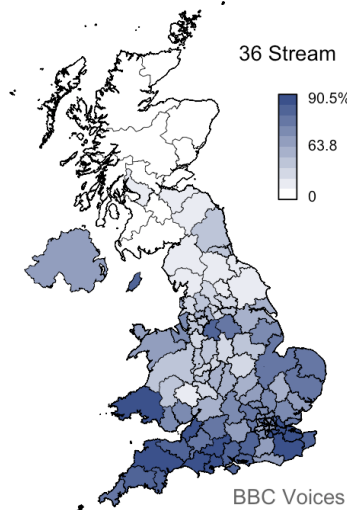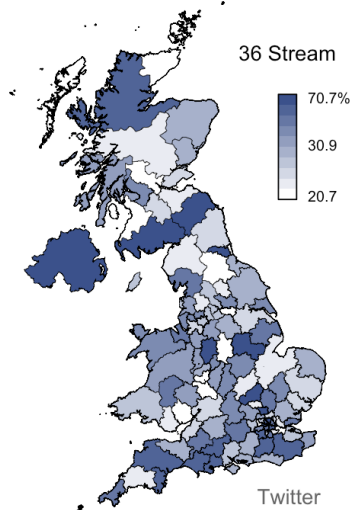

Supplement: Supplementary file 1 [file Presentation_1.zip › FRONTIERS_REPO/MAPS.pdf]
